# Time for change is now: Experiences of participants in a community-based approach for iron and folic acid supplementation in a rural county in Kenya, a qualitative study

**DOI:** 10.1371/journal.pone.0227332

**Published:** 2020-01-16

**Authors:** Mary Wanjira Kamau

**Affiliations:** School of Nursing Sciences, College of Health Sciences, University of Nairobi, Nairobi, Kenya; African Population and Health Research Center, KENYA

## Abstract

**Background:**

Iron and Folic Acid Supplementation (IFAS) is recommended by World Health Organization as part of antenatal care to prevent anaemia in pregnancy. In 2010, Kenya adopted this recommendation and the current policy is to provide one combined IFAS tablet for daily use throughout pregnancy, free of charge, in all public health facilities. However, adherence remains low over the years though anaemia in pregnancy remains high. Integration of IFAS into community-based interventions has been recommended because of its excellent outcome. Using Community Health Volunteers (CHVs) to distribute IFAS has not been implemented in Kenya before.

**Methods:**

Following an intervention study implementing a community-based approach for IFAS in five public health facilities in Lari Sub-County, 19 interviews were conducted among CHVs, nurses and pregnant women participating to describe their experiences. Thematic analysis of data was done using NVivo and findings described, with use of quotes.

**Findings:**

The nurses, CHVs and pregnant women were all positive and supportive of community-based approach for IFAS. They reported increased access and utilization of both IFAS and antenatal services leading to perceived reduction in anaemia and better pregnancy outcomes. Counselling provided by CHVs improved IFAS knowledge among pregnant women and consequent adherence. The increased IFAS utilization led to main challenge experienced being IFAS stock-outs. All participants recommended complementing antenatal IFAS distribution approach with community-based approach for IFAS.

**Conclusion:**

Using CHVs to implement a community-based approach for IFAS was successful and increased supplement awareness and utilization. However, the role of CHVs in IFAS programme implementation is not clearly defined in current policy and their potential in IFAS education and distribution is not fully utilized. All participants endorsed integration of community-based approach for IFAS into the antenatal approach to enhance IFAS coverage and adherence among pregnant women for better pregnancy outcomes.

## Introduction

Daily oral Iron and Folic Acid Supplementation (IFAS) is recommended by the World Health Organization (WHO) to prevent anaemia in pregnancy [1], reduce pregnancy complications and improve pregnancy outcomes. The Kenyan Ministry of Health adopted this high impact nutrition intervention in 2010 [2]. The current IFAS policy requires routine provision of IFAS tablets, free of charge, as part of Focused Antenatal Care (FANC) [3], to all pregnant women in all public health facilities. The ministry also developed various IFAS Information, Education and Communication (IEC) materials as part of the effort to improve IFAS coverage. Further, the ministry introduced one combined iron and folic acid tablet per day in 2012 to reduce side-effects associated with high iron levels to increase uptake and reduce tablet burden of separate formulations.

Despite these interventions, anaemia in pregnancy remains a public health problem in Kenya at 55.1% [2] and adherence with IFAS remains poor over the years, with less than 10% pregnant women taking IFAS tablets for 90 days or more, and about 30% not taking them at all [4]. Studies have reported numerous and diverse factors that affect IFAS adherence. These include: cost of IFAS tablets, tablets stock-outs, socio-economic status, IFAS side effects [5, 6], poor access and low utilization of antenatal services [7, 8] as well as inadequate and poor quality of IFAS counselling [8–11], among others. Consequently, the advantages of IFAS are not fully realized, underscoring the need for diverse strategies to strengthen supplementation.

The gap in integration of health interventions and strategies formed the basis for this study. Research has recommended community-based IFAS delivery [12], integration into primary health-care system [13] and community-based interventions [14, 15]. Nevertheless, little scientific evidence is available on integration of different health interventions and strategies especially at community level to improve the overall health status of all clients [16–18]. The experience of MICronutrients And Health (MICAH) program in Ghana and Malawi indicates that community-based administration of anaemia control interventions is critical to their success [19, 20]. The goal of the MICAH program was to improve the nutritional and health status of women and children through the most cost-effective and sustainable interventions, with a focus on micronutrients. It is therefore clear that antenatal distribution of IFAS using the channel of health facilities alone is useful but often inadequate, creating need to explore other delivery channels like community networks. The sustainability of intervention programs largely depends on community involvement. Consequently, an intervention study was developed to implement a community-based approach for IFAS in a rural County in Kenya.

Community based interventions involve use of volunteers/community agents, as point of contact/intermediary between intervention/health service and clients at community level. Witmer (1995) broadly defined community health workers as “community members who work almost exclusively in community settings and who serve as connectors between health care consumers and providers to promote health among groups that have traditionally lacked access to adequate care” [21]. Although community health workers are recognized by WHO and Global Health Workforce Alliance as an integral component of health workforce who are highly cost-effective [22, 23], most countries lack clear national policies or strategies for integrating community health workers’ programmes into the health care system [24].

Though the current Kenyan health policy recognizes community health workers in health care provision, they are currently referred to as Community Health Volunteers (CHVs) for lack of formal salary compensation. In this spirit of volunteerism and social reciprocity, there is a generally high dropout and turnover rate of CHVs [25]. Various other factors weaken their service delivery such as lack of clear training guidelines and job descriptions at the managerial level, unclear terms of service and poor linkage mechanisms, among others [25, 26]. All the same, the CHVs have well defined roles that include: maintaining personal and environmental hygiene; promoting good health seeking behavior; promoting health rights; provision of home based care to the chronically ill; defaulter tracing for immunization, TB and ART; referrals to health facilities for health care especially for the malnourished under-fives, pregnant women and the severely ill; and educating community members on various health topics such as reproductive health, with distribution of contraceptives, and safe motherhood, for example, the importance of pregnant women making four ANC visits and hospital delivery [27].

From the aforementioned, it is clear that, the CHVs neither have specific deliverables in relation to IFAS nor are they allowed to distribute IFAS tablets to pregnant women in their homes. Lessons learnt from this study where CHVs distributed IFAS tablets together with education can help inform policy on the role of CHVs in IFAS programme implementation. This approach has not been practiced in Kenya before, so experiences of various stakeholders have not been assessed. Therefore, the aim of this study was to describe the experiences of CHVs of participating in a community-based approach for IFAS. In addition, since pregnant women and nurses are key stakeholders as recipients and supervisors respectively, their experiences were also described. The specific objectives of the study were to (1) describe perceived benefits (2) identify challenges experienced and (3) describe recommendations of CHVs, nurses and pregnant women of participating in a community-based approach for IFAS.

## Methods

### Study setting

This study was conducted in March 2017 in Lari Sub-County, Kiambu County in Kenya. The study was embedded in a larger study involving implementation of a community-based approach for IFAS. Community health volunteers were trained to distribute IFAS tablets together with IFAS education to pregnant women in their homes. Various training materials used, developed and provided by the Ministry of Health, have been included under supporting information (S2 to S5). After training, CHVs were provided with Information, Education and Communication (IEC) materials developed by the Ministry of Health for educating pregnant women. These materials included community health workers IFAS counselling guides, posters and leaflets. The CHVs followed up the pregnant women on a weekly basis in their homes, during when they provided each woman with the entire week’s supply of IFAS, evaluated previous week’s intake and counselled her on different topics of IFAS. The nurses in charge of antenatal clinics where CHVs were attached were initially trained to provide supportive supervision to CHVs during the study and issue IFAS tablets to them for distribution to the pregnant women in their homes.

### Setting of the larger study

The larger study consisted of an intervention study that adopted a pretest-posttest quasi-experimental study design with a control group. Mixed methods approach of data collection for both quantitative and qualitative data were used. The study implemented a new community-based approach for iron and folic acid supplementation. In the control group, pregnant women were followed up at health facilities by nurses who issued IFAS tablets together with IFAS education, according to the standard practice. In the intervention group, trained CHVs distributed IFAS tablets together with IFAS education and followed up pregnant women in their homes on a weekly basis. Pregnant women were all followed up until the time of delivery of their babies when the delivery outcome was determined. Baseline and endline data were collected on the pregnant women’s knowledge, attitude and adherence to IFAS, otherwise referred to as compliance in the study, the main outcome of the study.

The study was conducted in the five largest public health facilities (Lari hospital, Kagwe, Kagaa, Githirioni and Kinale health centres) in Lari Sub-County in Kiambu County. The study was based in the community and maternal and child health (MCH) clinics whereas maternity units were used for evaluation of pregnancy outcome. Recruitment of respondents was done both in MCH and the community. Lari Sub-County is in the Western part of Kiambu County and is pre-dominantly rural. The Sub-County is further divided into five wards: Lari/Kirenga, Kijabe, Kamburu-Kamuchege, Kinale and Nyanduma. Lari has a total area of 439.20 square kilometers. According to the 2009 census, Lari Sub-County has a population of 123,895 people. Kiambu County covers an area of 2,543.42 km^2^ and is in the Central highlands of Kenya, close to Kenya’s capital city of Nairobi. The County has a population of 1,623,282 people, 51% of whom are females and has twelve sub-counties [28].

### Sampling

Two stage sampling was adopted to identify one Sub-County and five of its largest public health facilities where the study was conducted. Sampling frame consisted of all Sub-Counties in Kiambu County. The Sub-County with existing functional community units formed the basis for the intervention, meaning its CHVs were active in provision of community health services at the community level. The largest public health facilities, with highest client/patient population turnover, and with existing and functional (active) community units were used to implement the intervention. These largest public health facilities were considered because of the low turnover of antenatal clients in health facilities in Lari Sub-County. The methodology adopted in the larger study implementing a community-based approach for IFAS has also been published elsewhere [29, 30].

### Study population

The target population in this study was mainly CHVs participating in the community-based approach for IFAS. Nurses and pregnant women participating were also included in the study. A total of 30 nurses involved in provision of antenatal services in the entire Sub-county initially went through an update training on the IFAS programme. A total of 16 active CHVs were then trained on IFAS, out of which 12 consented to participate in the study. Nine of them participated up to the end and were therefore included in this study. Since CHVs are usually chosen by community members and are well known to them, both males and females were included. The two nurses in charge of antenatal services in the two largest public health facilities involved, who provided overall supportive supervision to the CHVs, were included in the study. The nurse in-charges are the representatives of all nurses working in the facility hence their use in this study, to provide key information on the community-based approach for IFAS on behalf of all the nurses in their facilities. Equally, eight pregnant women participants were randomly chosen, four from each of the two largest health facilities and interviewed. Since many pregnant women were involved in the study and we could not be able to collect qualitative data from all of them, to avoid bias, the eight were randomly chosen as they attended antenatal services on different days, thus ensuring diversity in the participant sample. This was to ensure both first time mothers and mothers on subsequent pregnancies were captured. By the eighth pregnant woman, they were repeating the same thing hence no more IDIs on pregnant women were conducted.

### Data collection and analysis

The researcher developed In-Depth Interview (IDI) guides for nurses, CHVs and pregnant women for data collection in English (S1). The interview guides for pregnant women were translated into Swahili. The interview guides focused on participants’ views of their experiences regarding various topics on community-based approach for IFAS including: perceptions, benefits, IFAS counselling provision, home visits’ evaluation, failures, challenges and recommendations. The researcher conducted all the interviews to ensure consistency with the help of one trained research assistant. The data was audio recorded during collection. A field diary was kept for back-up and to document happenings and write reflective notes that would inform final data analysis and interpretation.

Audio recorded data from the interviews were transcribed verbatim and translated into English (where applicable) then typed. The researcher imported interview transcripts into NVivo version 10 which was used to organize and analyze data. Theoretical thematic analysis was done using the six-step framework by Braun and Clarke (2013) as also outlined by Maguire and Delahunt (2017).

The first step was familiarization with the data which involved reading and re-reading transcripts, listening to audio-recorded data and writing down the initial impressions. The second step was generating initial codes through open coding. The first two transcripts were used to develop as many codes as possible. These codes were then modified into higher-order codes as more transcripts were read and re-read. The third step was searching for themes which involved examining the codes to identify and organize those fitting together into preliminary broader themes. The fourth step was reviewing the themes and involved modifying and developing preliminary themes further to make appropriate changes to ensure each theme was distinct. Higher order codes were grouped into sub-themes then organized into relevant overarching themes that best capture the content of the sub-themes and codes that built them. The fifth step was defining and naming the themes followed by the sixth step of writing up the final report where the findings were summarized according to the themes identified. The summary was then described in detail and representative quotations presented in the results for each theme that emerged [31–33].

The findings from each of the 3 categories of participants involved are presented separately.

### Ethics

The ethical approval for the study was granted by Kenyatta National hospital/University of Nairobi Ethics and Research Committee (KNH-ERC/A/90 protocol number—P706/11/2015). Research permit to distribute IFAS tablets through CHVs was obtained from the National Commission for Science, Technology and Innovation (NACOSTI/P/18/81499/2231). In addition, authority to conduct the study was obtained from administrative authorities of Kiambu County, Lari Sub-county and all health facilities participating in the study. Participation in the study was purely voluntary. All the study participants provided verbal and written informed consent before recruitment into the study. Ethical procedures observed have also been published elsewhere [29, 30].

## Findings

### Socio-demographic characteristics of study participants

The sociodemographic characteristics of the three categories of study participants are shown in Table 1. The age of CHVs and nurses ranged from 33 to 48 and 42 to 47 years respectively while all pregnant women were 30 years and below, the youngest being 19 years. All study participants were married.

**Table 1:** Profile of study participants.

Characteristic	Number (Percentage)		
	Nurses (n = 2)	Community Health Volunteers (n = 9)	Pregnant Women (n = 8)
**Age**			
Less than 40 years	0	3 (34)	8 (100)
41–45 years	1 (50)	4 (44)	0
Above 45 years	1 (50)	2 (22)	0
Range	42–47	33–48	19–30
**Gravidity**			
Primigravida	N/A	N/A	3
Multigravida	N/A	N/A	5
**Sex**			
Male	0	4 (44)	N/A
Female	2 (100)	5 (56)	8
**Marital Status**			
Married	2 (100)	9 (100)	8 (100)
Single	0	0	0
**Highest Education Level**			
Primary	0	2 (22)	6 (75)
Secondary	0	6 (67)	2 (25)
Tertiary	2 (100)	1 (11)	0

### Experiences of study participants on community-based approach for IFAS

When the participants were asked about their experiences on the community-based approach for IFAS based on various topics as stated earlier including: perceptions, benefits, IFAS counselling provision, home visits’ evaluation, failures, challenges and recommendations, three themes emerged namely perceived benefits, challenges experienced and recommendations regarding community-based approach for IFAS. The findings for each category of participants have been described below, most of the information being from CHVs who were the sole implementers of the community-based approach for IFAS, doing the follow-up to pregnant women through home visits.

### Experiences of community health volunteers on community-based approach for IFAS

#### Perceived benefits

When asked about their perceived benefits of community-based approach for IFAS, the CHVs reported numerous benefits. To begin with, all of them reported that they gained a lot of knowledge on IFAS. The CHVs were happy with trainings received and desired more regular updates. In addition, majority reported that it was a very enriching and satisfying experience to them, being of great help to pregnant women and contributing positively to community’s health in general. As some CHVs put it:

“…. the advantages are so many in the community than what we can think of”

*(Female CHV*, *health facility 2)*

“Personally, I think it has helped a lot, I can only see good things about it”

*(Female CHV*, *health facility 4)*.

“There are many benefits because I learned that IFAS increases the blood levels in a woman and it makes her with baby to be healthy. It helps the mothers to avoid so many problems which they face when giving birth. We learned the sources of iron and how to cook like spinach and not to overcook which we pass to the mothers and teach the same thus creating awareness to the community”

*(Female CHV*, *health facility 2)*.

Most CHVs reported having had very helpful and interactive sessions with pregnant women as they visited them in their homes. This was witnessed by reports CHVs received from pregnant women who reported having had open discussions with CHVs where they learnt a lot and received enormous support. The CHVs reported better adherence with IFAS among pregnant women and associated benefits including: increased antenatal care attendance, being more energetic, having sufficient blood levels, having no excessive bleeding, no complications at delivery, and increased babies’ birth weight thus healthier babies. One CHV reported a testimony from one of the mothers, who had a healthy twin delivery:

“She was very happy to see us at her home because if she did not take IFAS in order to increase her blood levels and improve on her pregnancy maybe there were going to be some complications…… she explained to me how she felt after giving birth that she had enough energy and did not have low blood levels.”

*(Female CHV*, *health facility 2)*

All the CHVs reported that IFAS utilization increased so much that this sub-county which had rarely experienced IFAS stock-outs before, experienced serious stock-outs during the study period. Concerning the increased IFAS utilization, some of the CHVs had this to say:

“…women were committing to take the IFAS…”

*(Female CHV*, *health facility 4)*

“When I came for the first time, I was taught on how to give IFAS to mothers in the community ………we started the job immediately and when we started we were able to get a lot of pregnant women in our villages and we taught them on how to use IFAS…….”

*(Male CHV*, *health facility 2)*

“The number of clients who have come to the facility has increased because we are able now to reach them, to reach more clients so that they can come to the facility and ….”

*(Female CHV*, *health facility 3)*

In addition, some of the CHVs reported increased early antenatal services utilization, going against the common practice of starting antenatal clinics late. Besides, increase in health services utilization was not just for antenatal services but other health services also like laboratory, immunization and family planning services among others. Some quotes by CHVs to this effect are included below:

“….and when they get a pregnant woman, they always refer her to me.”

*(Female CHV*, *health facility 4)*

“Aaaah, they are the ones who were looking for you to tell you that so and so is pregnant, and they need to begin ANC early……”

*(Female CHV*, *health facility 3)*

“…we have visited them during this program of IFAS and they have been taking them and now they are teaching the other women about the importance of taking IFAS ………and other services as well”

*(Female CHV*, *health facility 1)*

“Some mothers have their own problems and I usually go; we talk with them and we solve…… So, I do referrals”

*(Female CHV*, *health facility 3)*

#### More benefits: Closer communication improved health education and IFAS counselling

The CHVs shared their experiences in providing IFAS education and counselling. The CHVs reported that they were able to tackle side effects that hindered women from taking IFAS by educating them. Addressing side-effects and their management led to better adherence as opposed to the common practice of stopping IFAS with every slight discomfort/side-effect. Sharing her experience, one CHV had this to say:

“It is good because like now the ones who come to the facility when they are given the IFAS then return home and have side effects, they stop taking them. But if you give them in the community, if they have side effects on taking them, you talk and encourage them to keep on taking them, that way I saw it is good.”

*(Female CHV*, *health facility 1)*

The home visits made by CHVs presented an excellent opportunity for closer interaction with the clients and for other health messages as well. They reported better relations with the community, associated with better communication with clients and more consultations from them. This helped create awareness in the community not only about IFAS but other health issues as well, thus improving general community understanding on health matters. Furthermore, most CHVs reported that, through this approach, peer education and counselling was enhanced among peers of pregnant women leading to better understanding of the importance of taking IFAS. As a few of them said, this was evidenced by pregnant women referring their fellow pregnant women to CHVs early in pregnancy to start IFAS, immediately they conceived. This was fueled by the benefit they had seen from the pregnant women the CHVs were already distributing IFAS to. Here are some of their quotes:

“…. some of the pregnant women brought their friends to me to start taking IFAS immediately they knew they were pregnant.”

*(Female CHV*, *health facility 1)*

“…and I encourage them to refer to me other pregnant women because some of them know these pregnant women… …”

*(Female CHV*, *health facility 5)*

“I personally felt good because now I had something that I would be taking to them and teach them about IFAS in details. I felt that this was going to help many women”

*(Female CHV*, *health facility 5)*.

Majority of the CHVs equally reported better relations with the health facilities’ staff. They reported becoming more conversant with the hospital and how it operates in the various departments, which they did not know before. In addition, they were able to interact with the nurses more, unlike before and respect each other more in their respective roles.

#### Challenges: Community-based approach for IFAS has its own share of problems which can be overcome

One of the major challenges all CHVs experienced was IFAS stock-outs, owing to the reported increased IFAS utilization in the Sub-County. One CHV reported that:

“I came to the hospital to get the supplements and I was told they were not there and at that time women were waiting for me to take them to their places”

*(Female CHV*, *health facility 2)*

However, this was circumvented by the researcher engaging management of health facilities to acquire more IFAS supplies. The CHVs reported that delay caused by bureaucratic processes of acquiring health supplies reduced their morale.

Some CHVs, though very few compared to the majority, reported facing frustrations regarding the home visits. Whereas a few women were living in hard-to-reach areas, a few others did not embrace home visits fully, so they became uncooperative. A few CHVs attributed this to negative attitude and mood swings in pregnancy, whereby some clients did not want anyone to intrude into their privacy. It could also be attributed to failure of the CHV to inform their husbands first. Except for those husbands who accompanied their wives for antenatal services, this approach was a new concept which was not easy for the pregnant women to explain to their husbands. The CHVs reported that failure to talk to the husband first would have made some of the few pregnant women uncooperative. In addition, a few CHVs sometimes missed the clients both at home and on phone or pregnant women gave the phone contacts of their husbands instead. When asked to share the problems they encountered during the home visits, this is how some CHVs described their experiences:

“I can say dealing with pregnant women is not easy since they tend to have bad moods and I would find one in a bad mood who would not even want us to talk and she thinks the IFAS are for my own benefit and not hers”

*(Female CHV*, *health facility 3)*

“Some women think that I am so concerned with her life and they see as if I have become a bother to them.”

*(Female CHV*, *health facility 1)*

“….at times you call someone, the husband picks the phone, you talk for some time trying to explain who you are to them because that is someone’s else wife you called….”

*(Male CHV*, *health facility 2)*

“Just a little bit, you know for me as a man, visiting peoples’ home week after week might raise some questions somewhere. But now considering that I have been doing other jobs at the grass roots level it was not a huge challenge because I had explained well to them why I was following up on IFAS for pregnant women”

*(Male CHV*, *health facility 1)*

A few of the CHVs interviewed reported experiencing fear of the unknown at the beginning of the study. Their perceptions revealed that some CHVs were fearful of the women’s reactions as the study began. The CHVs feared non-acceptance or rejection from both pregnant women and the community. To their surprise, this did not turn out so. Instead, the intervention was well received by the entire community, went on very well and women were positive and cooperative. One of them said:

“We thought it was going to be hard to convince the mothers to accept these supplements”

*(Male CHV*, *health facility 5)*

The other major challenge reported by all CHVs was lack of monthly remuneration. However, the researcher provided USD 150 per month to facilitate their fare and transport for weekly home visits. Lack of monthly salary becomes a challenge and interferes with their commitment because they must search for other income generating activities to provide for their families. Sharing his frustrations in lack of salary, one CHV said:

“The major problem is lack of facilitation.”

*(Male CHV*, *health facility 1)*

#### Recommendations: Suggestions for improvement

The CHVs recommended their official recognition. All CHVs unanimously asked for continued motivation and monthly salary. They reported that community work is very involving and getting no compensation for it does not augur well with their roles as bread winners, since at the end of the day, they need to put food on the table.

Of great concern to some of the CHVs was the need for more advocacy, social mobilization, sensitization and general meeting with all stakeholders, particularly husbands of pregnant women. All the CHVs also recommended consistent IFAS supply and continuous follow-up of pregnant women. Two CHVs said:

“In short, taking IFAS from the hospital or facility should not be the end but there should be ways of following up especially to the grass roots because I have realized that some of the mothers who get IFAS from the hospital throw them in the toilets or they just keep and do not take them”

*(Male CHV*, *health facility 1)*.

“They are supposed to give us the IFAS so that we can be able to do our work of delivering them to the mothers because of the ones that we call defaulters”

*(Male CHV*, *health facility 2)*.

To sum it all, the CHVs recommended permanent adoption of the community-based approach into IFAS distribution to complement facility distribution. They suggested integration of both strategies to reach all pregnant women as illustrated in these quotes:

“If we leave them on their own after educating them a little in the hospitals we won’t get any benefits from this program … …because I have realized that the mothers who get IFAS from the hospital, some throw them in the toilets or just keep them after collecting them …….so I would love it to be made permanent”

*(Female CHV*, *health facility 2)*.

### Experiences of nurses on community-based approach for IFAS

The following are the experiences of the two nurse in-charges of antenatal services in the two largest public health facilities involved, who are the representatives of all nurses working in their facilities.

#### Perceived benefits

When the nurses were asked about their perceived benefits of community-based approach for IFAS, they greatly liked the approach. They were really impressed and encouraged by the IFAS training provided. The nurses found this approach timesaving on time spent counselling clients. This was an advantage to them which helped address their heavy workload that often affected their quality of IFAS counselling. Sharing their views, they said:

“It was not tedious on our side. When the client is supposed to take IFAS from CHV, you spend less time with them.”

*(Nurse in charge of ANC services*, *health facility 2)*

“… …we tell them about IFAS, but this always depends on the workload.”

*(Nurse in charge of ANC services*, *health facility 1)*

Based on their experience, nurses identified various benefits of community-based approach for IFAS to pregnant women. First, closer follow-up by CHVs led to more acceptance and uptake of IFAS thus higher levels of utilization and adherence. Secondly, antenatal clinic attendance improved. Thirdly, nutritional status of pregnant women improved due to consistent nutritional education offered by CHVs. Fourthly, access to IFAS improved since the women could always get IFAS at home reducing missed opportunities for IFAS distribution. Consequently, cases of low haemoglobin levels/anaemia during pregnancy and delivery reduced as evidenced by this quote by one nurse:

“…. antenatal care attendance improved because CHVs were really mobilizing mothers to come to the clinic. For the deliveries we had, I would say we did not have many serious cases due to low Hb. Also, IFAS adherence improved.”

*(Nurse in charge of ANC services*, *health facility 1)*

Nurses perceived that this approach bridged the gap between health facility and community. It also brought the nurses and CHVs closer, improving their working relationships, as a team. They reported that harmonious working enabled pregnant women trust CHVs more. This enhanced their confidence to consult CHVs over other health matters thus leading to improved community health in general. One nurse said:

“This was a good project because it is a gap and an area which health workers have not taken seriously.”

*(Nurse in charge of ANC services*, *health facility 1)*

#### Challenges experienced and recommendations

The main challenge nurses experienced was IFAS stock-outs. They reported that this had not been reported in the entire Sub-County before and that it was due to improved antenatal clinic attendance and increased IFAS utilization. The perceived benefit of improved antenatal clinic attendance thus became a challenge to the nurses. In addition, improved antenatal attendance led to increased workload among nurses perpetuating the problem of limited time with client to provide detailed quality counselling, as indicated by this quote:

“Even though we give them IFAS at the clinic, due to workload, we do not have time to emphasize on its importance.”

*(Nurse in charge of ANC services*, *health facility 1)*

Finally, when asked which strategy they preferred to use for IFAS distribution, nurses recommended integration of both hospital and community-based distribution as supported by this quote:

“I think in future, they should be really combined”

*(Nurse in charge of ANC services*, *health facility 1)*

One of the nurses emphasized that integration is only okay if the community-based approach for IFAS is practiced professionally with appropriate and timely referrals where need be, to avoid mismanagement of pregnant women. She said:

“I would say the health professionals are much better, we cannot solely give the responsibility to the CHVs. We have to do this professionally, bearing in mind the woman is pregnant… …there can be other problems.”

*(Nurse in charge of ANC services*, *health facility 2)*

Moreover, they all recommended more community-based education and good IFAS supply to curb stock-outs as supported by the following quotes:

“There was a challenge, we ran out of stock when the program was halfway”

*(Nurse in charge of ANC services*, *health facility 2)*

“……they should make sure that there is consistency in the supply of IFAS”

*(Nurse in charge of ANC services*, *health facility 1)*

“……empower people with more effective counselling, even to the health workers”

*(Nurse in charge of ANC services*, *health facility 1)*

### Experiences of pregnant women on community-based approach for IFAS

#### Perceived benefits

To explore the experiences of pregnant women as the recipients of the community-based approach for IFAS, a few were similarly interviewed to share their experiences. The women reported that the experience of using community-based approach for IFAS was very good, helpful and not problematic at all. The pregnant women reported:

“It had no problems, it was good……. They have never failed to come and… …”

*(ANC woman*, *health facility 1)*

“…and the program is good, it really helps people”

*(ANC woman*, *health facility 2)*

The women interviewed reported several benefits. First, convenience of receiving regular supply of IFAS at home and not waiting until the next antenatal clinic. Second, IFAS was readily accessible and available even to those far away from the hospital. Third, they appreciated the fact that they did not have to spend money on bus fare just to go for IFAS tablets. Fourth, some were pleased with the visits because they did not have to stop their chores. Fifth, health education offered enabled them to manage any IFAS side-effects experienced, appropriately. Sixth, close follow-up by CHVs provided an opportunity to consult on other health problems hence were helped comprehensively. Overall, they were happy with the outcome and reported increased blood levels on testing. Referring to easy IFAS access, some pregnant women said:

“The ones who are far will access the supplements easily ……… I come from far, so it is easy for them to bring me the supplements and the transport has reduced”

*(ANC woman*, *health facility 2)*

Concerning other benefits, the pregnant women said:

“This one for the community approach is good because they must bring IFAS for you, and they emphasize on the importance. You see that they consider it important and you also become serious”

(*ANC woman*, *health facility 2)*.

“They increase the amount of blood after taking them for some time……

Another one is that when I take them, they help me in my body and the child”.

“.…he then told me about taking foods which help in raising the blood levels”

(*ANC woman*, *health facility 1)*.

#### Challenges experienced and recommendations

Most of the women reported that they did not experience any challenges with this approach of IFAS. Only one woman reported that although the CHV visited her weekly, there are times he failed to come on time including times when client did not have supplements.

“……sometimes he did not come on time and kept me waiting”

(*ANC woman*, *health facility 1*, *IDI)*

Overwhelmingly, most women were satisfied with how study was implemented. All pregnant women involved highly suggested its continuation.

“It is good for the approach to continue because it will help a lot of people since majority do not know about IFAS and they need to know more … …those at home will get to know better, about IFAS”

*(ANC woman*, *health facility 2)*

## Discussion

The main findings from this study were: (1) all the three groups of study participants were positive and supportive of the community-based approach for IFAS (2) provider-client communication was improved at both community and facility levels (3) there was perceived increased use of not only IFAS services and tablets but other health services as well (4) community-based education yielded positive results and needs further strengthening (5) there was perceived increased IFAS utilization than ever before in the Sub-county leading to the main challenge experienced being IFAS stock-outs (6) continued use of community-based approach for IFAS by integration into the existing antenatal distribution was highly recommended by all participants.

These findings are consistent with literature on strengths of community based distribution of IFAS as a valuable platform in implementation of IFAS programmes [34]. The study participants found this approach beneficial to not only the pregnant women but also to all stakeholders and the entire community. They were all positive and supportive of the approach. Using CHVs to provide IFAS tablets and education was a very satisfying experience to them. As other studies indicate, CHVs perform diverse functions related to health care delivery especially in developing nations due to shortage of health care providers [35, 36]. During the community-based approach for IFAS, CHVs felt valued and appreciated in that they made significant contributions to improvement of the health of pregnant women, as witnessed in other studies [25, 27]. This encouraged CHVs to be more committed. In turn, pregnant women benefitted more by consistently receiving closer follow-up from CHVs. Thus, involving CHVs in community-based approach for IFAS can go a long way in ensuring the benefits of IFAS are realized.

Following this study, communication among the various stakeholders was enriched. As they reported, the nurse-CHV-client interaction was improved both at facility and community levels. There was closer interaction between nurses, CHVs and pregnant women. Consistent with other studies, CHVs acted as a link to facilitate access to health services by clients and informed nurses about community health needs [21] especially for those living in rural and underserved areas [37, 38]. Since the nurses in charge of the health facilities where CHVs were attached provided supportive supervision and IFAS tablets to CHVs, this link was activated more thus more opportunities to communicate and exchange ideas. This increased community awareness and uptake of IFAS. As Titaley (2014) indicates, community participation in health programs are necessary to improve their uptake. This cannot be achieved without proper communication. The CHVs are closer to the community leading to presumably better interpersonal communication with community members than health care providers. In this regard, equipping them with the necessary IFAS information in a simple way can achieve more and help address related fears or misconceptions. This in turn increases understanding of the importance of taking supplements.

One of the unanticipated benefits of the study was increased use of not only IFAS but also other health services, as reported by CHVs and nurses. They reported that pregnant women developed more confidence in CHVs causing more consultations and referrals for health services. This increased utilization of varied health services in addition to increased antenatal care attendance and IFAS utilization, as reported in this study. Numerous studies likewise indicate great success with community-based interventions. Using community health volunteers reduced maternal anaemia in Thailand, [39] and Nepal [40]. In these studies, these community health volunteers actually contributed to increasing antenatal care attendance, dispelling the myth that community distribution discourages women from seeking care at health facilities [41]. Furthermore, other studies indicate that using community agents significantly improved antenatal and neonatal practice indicators [42] on top of reducing maternal/child morbidity and mortality [43, 44]. For example training traditional birth attendants in a highly dispersed rural African community with limited access to healthcare, significantly reduced neonatal mortality by up to 50% [45]. Also, using community health workers reduced adverse perinatal outcomes in meta-analysis of several trials [42, 46]. This shows the value and benefit of using community agents to improve the most basic and important health indicators.

Community-based IFAS education, including peer counselling which was positively implicated in a study in Bangladesh [47], yielded positive results in this study and needs further strengthening. Likewise, the importance of family support, especially husbands, in IFAS use is critical, just as indicated in a study in Zimbabwe [48]. As Titaley stated, strengthening counselling sessions during antenatal care alone without improving community-based education is not sufficient to improve IFAS uptake [49]. Pregnant women should be provided with detailed counselling on anaemia control [50]. Expanding community-based services and sources of supplements by expanding local, community-based providers delivering anaemia counselling and education services and IFAS tablets improved IFAS utilization in Indonesia [51]. Lack of clarity on benefits of IFAS during pregnancy [10] due to inadequate counselling [9] is one of the factors affecting adherence [52]. Counselling enables clients to gain understanding to take initiative and create demand for services. Formative research studies have emphasized the need for community-based counselling [34, 52, 53]. Nurses in this study confessed providing inadequate IFAS counselling. On the contrary, pregnant women indicated positive learning experiences from CHVs. The pregnant women appreciated this approach, stating that they learned a lot, freely and with ease. Thus, the need to strengthen community-based education (offering health education at the community level) cannot be over-emphasized.

Following this study, the CHVs and nurses reported increased IFAS utilization than ever before leading to the main challenge experienced being IFAS stock-outs. Stock-outs of IFAS tablets had never been experienced before in this Sub-county, suggesting the high utilization following implementation of this study. It was a major concern for all the three categories of study participants since it had never happened before. Stock-outs [54, 55] and inconsistent IFAS supply [53] have been cited as a challenge and foremost barrier to effective IFAS use. Through this approach, a demand for IFAS was created which could not be met by existing resources. This reveals that health facilities underestimate the quantity of required resources due to probable underutilization of their health services. Replenishing the stock was not easy because of the bureaucratic government systems that were time-consuming. Therefore, there is need to address stock replenishing procedures to ensure consistent supply of IFAS throughout pregnancy. Use of a complementary push and pull mechanism as applied by Omotayo and colleagues in their study [56] would be of great help. However, this had not been considered at the beginning of the study since it was unexpected now that IFAS stock-outs had never been experienced before in the entire Sub-County.

Findings from this study indicate and recommend that the time for integration of community-based approach into existing vertical health facility approach in provision of IFAS services is now. All the study participants suggested and desired sustenance of community-based approach for IFAS. From this and the larger study where this current study was embedded, it shows that, the potential of CHVs in the IFAS programme, in distribution and education, is not fully utilized since they do not have specific tasks, records or trainings in relation to the IFAS programme. Through the community health strategy, CHVs should be involved more in IFAS programme. Nisar et al (2014) recommended development of interventions that provide sufficient information and quality counselling to increase IFAS coverage, and community-based approach for IFAS is one of such [57].

Despite the contribution of CHVs in both preventive and curative services, work environment challenges and weakening characteristics influence their functionality and sustainability [58]. These include: workload, supportive supervision, supplies and equipment, and respect from community and health system, affecting their productivity [37]. Working on voluntary basis without a regular compensation for services rendered is one of the set-backs in their active involvement as reported in this and other studies [25, 27]. For the few CHVs who are active, they do not have specific tasks or records in relation to the IFAS programme. Before this study, they had never been specifically trained on IFAS. Since CHVs are already working with communities to improve their general health, together with their leaders and the health facilities, they should come up with more specific IFAS related tasks, activities and terms of reference, for consideration by the Ministry of Health, in application of the bottom-up approach. Therefore, the Ministry of Health should formally recognize and officially absorb CHVs into the existing health care system by including them in the pay roll to earn a salary. The Ministry should also increase IFAS supply by applying the push and pull mechanism [56] to avoid IFAS stock-outs.

### Strengths and limitations

This study utilized qualitative methods of in-depth interviews to obtain as much information from the study participants as possible, of their experiences in community-based approach for IFAS. The study reveals that CHVs have a role to play in implementation of IFAS programme. In addition, weaknesses in the current system of acquiring health resources were revealed, probably indicating an underlying bigger problem of underutilization. Some of the limitations in this study include more focus on information from CHVs, who were the sole implementors of this approach who conducted follow-ups to pregnant women through home visits, hence the sample size for nurses and pregnant women was smaller.

## Conclusion

In conclusion, there was a perception that using CHVs to implement a community-based approach for IFAS was successful in increasing supplement awareness and utilization. It may have the potential to increase the coverage and adherence if sustained. The CHVs, nurses and pregnant women were all positive about this approach and embraced it with a lot of enthusiasm. However, the role of CHVs in IFAS programme is not clearly defined in the current IFAS policy and their potential in IFAS programme implementation is not fully utilized. The community-based approach is a potential approach for strengthening IFAS policy implementation in Kenya. It can be used to increase both uptake and delivery of IFAS to pregnant women to improve the low adherence levels. There is need to complement the antenatal IFAS distribution approach with community-based approach for IFAS to strengthen IFAS programme interventions to further increase supplementation coverage and consequently reduce deficiencies of these crucial micronutrients among women and children.

Further research is needed to determine the feasibility of integration of community-based approach for IFAS into the existing antenatal distribution, by allowing community health volunteers to distribute supplements in the community. The role of CHVs in IFAS programme implementation also needs to be specified. These are policy initiatives worth considering, in an effort to strengthen IFAS policy implementation in Kenya. The community-based approach may be a promising strategy because it has the potential to immediately increase access to IFAS supplements by pregnant women. It’s high time for change in relation to integrating the community-based approach for IFAS into the existing vertical health facility approach in provision of IFAS services.

## Supporting information

S1 File Interview guides. This consists of three parts including: part A- Nurses’ key in-depth interview guide used to collect key information from the nurses; part B—Community health volunteers’ in-depth interview guide—the interview guide used to collect data from the community health volunteers; part C—Pregnant women’s in-depth interview guide—the interview guide used to collect data from the pregnant women.
(DOCX)

S2 File Community health workers IFAS counselling guide, Swahili. This is the main training material used for training community health volunteers.
(PDF)

S3 File Accelerating reduction of iron deficiency anaemia among pregnant women in Kenya, plan of action 2012–2017. This is one of the training materials used for training the community health volunteers.
(PDF)

S4 File National IFAS communication strategy 2013–2017. This is one of the training materials used for training the community health volunteers.
(PDF)

S5 File The link for Information, Education and Communication (IEC) materials on IFAS. This is the link where more of the supplementary materials used for IFAS training can be found.
(DOCX)

## References

[1] WHO. Essential Nutrition Actions: Improving maternal, newborn, infant and young child health and nutrition. Geneva: World Health Organization; 2013. 25473713

[2] MoH. National Policy Guideline on combined iron and folic acid (IFA) supplementation for pregnant mothers in Kenya. In: Division of Nutrition, editor. Nairobi2013.

[3] MoH. Accelerating reduction of iron deficiency anaemia among pregnant women in Kenya: Plan of action 2012–2017. In: Division of Nutrition, editor. Nairobi2012.

[4] KNBS, Macro ICF. Kenya Demographic and Health Survey 2014. Nairobi: Kenya National Bureau of Statistics; 2015.

[5] Maina-Gathigi L, Omolo J, Wanzala P, Lindan C, Makokha A. Utilization of folic acid and iron supplementation services by pregnant women attending an antenatal clinic at a regional referral hospital in Kenya. Maternal and child health journal. 2013;17(7):1236–42. Epub 2012/08/22. 10.1007/s10995-012-1120-x . 22907273

[6] Gebremedhin S, Samuel A, Mamo G, Moges T, Assefa T. Coverage, compliance and factors associated with utilization of iron supplementation during pregnancy in eight rural districts of Ethiopia: a cross-sectional study. BMC public health. 2014;14:607 10.1186/1471-2458-14-607 . 24930036 PMC4073172

[7] Pal PP, Sharma S, Sarkar TK, Mitra P. Iron and Folic Acid Consumption by the Ante-natal Mothers in a Rural Area of India in 2010. Int J Prev Med. 2013;4(10):1213–6. Epub 2013/12/10. . 24319564 PMC3843311

[8] Nisar YB, Dibley MJ, Mir AM. Factors associated with non-use of antenatal iron and folic acid supplements among Pakistani women: a cross sectional household survey. BMC Pregnancy Childbirth. 2014;14:305 Epub 2014/09/06. 10.1186/1471-2393-14-305 . 25189220 PMC4162926

[9] Galloway R, Dusch E, Elder L, Achadi E, Grajeda R, Hurtado E, et al Women’s perceptions of iron deficiency and anemia prevention and control in eight developing countries. Soc Sci Med. 2002;55 10.1016/s0277-9536(01)00185-x 12188461

[10] Mithra P, Unnikrishnan B, Rekha T, Nithin K, Mohan K, Kulkarni V, et al Compliance with iron-folic acid (IFA) therapy among pregnant women in an urban area of south India. African Health Sciences. 2014;14(1). 10.4314/ahs.v14i1.39 PMC4449048 26060488

[11] MoH. IFAS Dialogue guide for health care providers. In: Division of Nutrition, editor. Nairobi2012.

[12] Siekmans K, Roche M, Kung’u JK, Desrochers RE, De-Regil LM. Barriers and enablers for iron folic acid (IFA) supplementation in pregnant women. Maternal & Child Nutrition. 0(0):e12532 10.1111/mcn.12532 29271115 PMC6865983

[13] Charoenlarp P, Dhanamitta S, Kaewvichit R, Silprasert A, Suwanaradd C, Na-Nakorn S, et al A WHO collaborative study on iron supplementation in Burma and in Thailand. Am J Clin Nutr. 1988;47. 10.1093/ajcn/47.2.280 2963533

[14] Alam A, Rasheed S, Khan NUZ, Sharmin T, Huda TM, Arifeen SE, et al How can formative research inform the design of an iron-folic acid supplementation intervention starting in first trimester of pregnancy in Bangladesh? BMC Public Health. 2015;15:374 10.1186/s12889-015-1697-2 25887449 PMC4425912

[15] Bhutta ZA, Darmstadt GL, Hasan BS, Haws RA. Community-based interventions for improving perinatal and neonatal health outcomes in developing countries: a review of the evidence. Pediatrics. 2005;115(2 Suppl):519–617. 10.1542/peds.2004-1441 . 15866863

[16] Kc A, Thapa K, Pradhan YV, Kc NP, Upreti SR, Adhikari RK, et al Developing community-based intervention strategies and package to save newborns in Nepal. J Nepal Health Res Counc. 2011;9(2):107–18. . 22929839

[17] Shah NM, Brieger WR, Peters DH. Can interventions improve health services from informal private providers in low and middle-income countries?: a comprehensive review of the literature. Health Policy Plan. 2011;26(4):275–87. 10.1093/heapol/czq074 . 21097784

[18] Community Directed Interventions Study Group. Community-directed interventions for priority health problems in Africa: results of a multicountry study. Bull World Health Organ. 2010;88(7):509–18. Epub 2010/07/10. 10.2471/BLT.09.069203 . 20616970 PMC2897985

[19] Yip R. Iron supplementation: country level experiences and lessons learned. Journal of Nutrition. 2002;132(859S–861S). 10.1093/jn/132.4.859S 11925498

[20] MICAH. Malawi final survey report. World Vision. 2006.

[21] Witmer A, Seifer SD, Finocchio L, Leslie J, O’Neil EH. Community health workers: integral members of the health care work force. American Journal of Public Health. 1995;85(8_Pt_1):1055–8. 10.2105/ajph.85.8_pt_1.1055 7625495 PMC1615805

[22] Perry H, Zulliger R. How Effective are Community Health Workers? An Overview of Current Evidence with Recommendations for Strengthening Community Health Worker Programs to accelerate Progress in Achieving the Health-related Millennium Development Goals. HRH Global Resource Center Johns Hopkins Bloomberg School of Public Health, International Health and Health BaS; 2012.

[23] Vaughan K, Kok MC, Witter S, Dieleman M. Costs and cost-effectiveness of community health workers: evidence from a literature review. Hum Resour Health. 2015;13:71 10.1186/s12960-015-0070-y . 26329455 PMC4557864

[24] WHO. Strengthening The Performance Of Community Health Workers In Primary Health Care: report of a WHO Study Group. Geneva: Group WS; 1989. 2497585

[25] Ministry of Health. Strategy for Community Health 2014–2019. In: Community Health Service Unit, editor. Nairobi. 2014.

[26] Ministry of Public Health and Sanitation. National Communication Strategy for Community Health Services. In: Community Health Services Unit, editor. Nairobi. 2012.

[27] European Commission. What drives community health services in Kenya, Position Paper No.1. Nairobi: Austrian Development Cooperation; 2012.

[28] COUNTY GOVERNMENT OF KIAMBU. Politcal Units 2018 [cited 2018 14/01/2018]. https://kiambu.go.ke/political-units/.

[29] Kamau MW, Mirie W, Kimani S. Compliance with Iron and folic acid supplementation (IFAS) and associated factors among pregnant women: results from a cross-sectional study in Kiambu County, Kenya. BMC public health. 2018;18(1):580 10.1186/s12889-018-5437-2 29720135 PMC5930505

[30] Kamau M, Kimani S, Mirie W. Counselling on iron and folic acid supplementation (IFAS) is associated with improved knowledge among pregnant women in a rural county of Kenya: a cross-sectional study [version 1; referees: awaiting peer review]. AAS Open Research. 2018;1(21). 10.12688/aasopenres.12891.2 PMC7118767 32259021

[31] Burnard P, Gill P, Stewart K, Treasure E, Chadwick B. Analysing and presenting qualitative data. Br Dent J. 2008;204(8):429–32. Epub 2008/04/29. 10.1038/sj.bdj.2008.292 . 18438371

[32] Clarke V, Braun V. Teaching thematic analysis: Overcoming challenges and developing strategies for effective learning. Psychologist. 2013;26(2):120–3.

[33] Maguire M, Delahunt B. Doing a thematic analysis: A practical, step-by-step guide for learning and teaching scholars. All Ireland Journal of Higher Education; Vol 9, No 3 (2017). 2017.

[34] Kavle JA, Landry M. Community-based distribution of iron–folic acid supplementation in low- and middle-income countries: a review of evidence and programme implications. Public Health Nutrition. 2017;21(2):346–54. Epub 10/24. 10.1017/S1368980017002828 29061205 PMC10261080

[35] Kane S, Gerretsen B, Scherpbier R, Dal Poz M, Dieleman M. A realist synthesis of randomised control trials involving use of community health workers for delivering child health interventions in low and middle income countries. BMC Health Serv Res. 2010;10 10.1186/1472-6963-10-286 20942900 PMC2964693

[36] Bigirwa P. Effectiveness of community health workers (CHWS) in the provision of basic preventive and curative maternal, newborn and child health (MNCH) interventions: A systematic review. Health Policy and Development. 2009;7(3):162–72.

[37] Jaskiewicz W, Tulenko K. Increasing community health worker productivity and effectiveness: a review of the influence of the work environment. Human Resources for Health. 2012;10:38-. 10.1186/1478-4491-10-38 23017131 PMC3472248

[38] George A, Young M, Nefdt R, Basu R, Sylla M, Clarysse G, et al Community Health Workers Providing Government Community Case Management for Child Survival in Sub-Saharan Africa: Who Are They and What Are They Expected to Do? The American Journal of Tropical Medicine and Hygiene. 2012;87(5 Suppl):85–91. 10.4269/ajtmh.2012.11-0757 23136282 PMC3748527

[39] Pattanee W. Prevention and Control of Anemia: Thailand Experiences. Journal of Nutrition. 2002;132(862S–866S). 10.1093/jn/132.4.862S 11925499

[40] Sanghvi TG, Harvey PW, Wainwright E. Maternal iron-folic acid supplementation programs: evidence of impact and implementation. Food Nutr Bull. 2010;31(2 Suppl):S100–7. Epub 2010/08/19. 10.1177/15648265100312S202 . 20715594

[41] MCHIP. Community-based distribution for routine Iron/folic acid supplementation in pregnancy. Maternal and Child Health Integrated Program, USAID, Nairobi: 2014.

[42] Gogia S, Sachdev HS. Home visits by community health workers to prevent neonatal deaths in developing countries: a systematic review. Bulletin of the World Health Organization. 2010;88(9):658–66B. 10.2471/BLT.09.069369 20865070 PMC2930362

[43] Lassi ZS, Haider BA, Bhutta ZA. Community-based intervention packages for reducing maternal and neonatal morbidity and mortality and improving neonatal outcomes. Cochrane Database Syst Rev. 2010;(11):CD007754 10.1002/14651858.CD007754.pub2 . 21069697

[44] Lewin SA, Babigumira SM, Bosch-Capblanch X, Aja G, Van Wyk B, Genton C, et al Lay health workers (LHWs) in primary and community health care: A systematic review of trials. Cochrane Database Syst Rev: 2005. 10.1002/14651858.CD004015.pub2 15674924

[45] Gill CJ, Phiri-Mazala G, Guerina NG, Kasimba J, Mulenga C, MacLeod WB, et al Effect of training traditional birth attendants on neonatal mortality (Lufwanyama Neonatal Survival Project): randomised controlled study. BMJ. 2011;342. 10.1136/bmj.d346 PMC3032994 21292711

[46] Wilson A, Gallos ID, Plana N, Lissauer D, Khan KS, Zamora J, et al Effectiveness of strategies incorporating training and support of traditional birth attendants on perinatal and maternal mortality: meta-analysis. BMJ. 2011;343. 10.1136/bmj.d7102 PMC3228291 22134967

[47] Ara G, Khanam M, Papri N, Nahar B, Haque MA, Kabir I, et al Peer counselling improves breastfeeding practices: A cluster randomized controlled trial in urban Bangladesh. Maternal & Child Nutrition. 2018;14(3):e12605 10.1111/mcn.12605 29660858 PMC6055706

[48] Tinago CB, Annang Ingram L, Blake CE, Frongillo EA. Individual and structural environmental influences on utilization of iron and folic acid supplementation among pregnant women in Harare, Zimbabwe. Maternal & Child Nutrition. 2017;13(3):e12350 10.1111/mcn.12350 27502366 PMC6866096

[49] Titaley CR. Factors associated with not using antenatal iron/folic acid supplements in Indonesia: the 2002/2003 and 2007 Indonesia Demographic and Health Survey. Asia Pacific journal of clinical nutrition. 2014;23(4). 10.6133/apjcn.2015.24.1.10 25740755

[50] Campaore A, Gies S, Brabin BJ, Tinto H, Brabin L. “There is iron and iron…” Burkinabè women’s perceptions of iron supplementation: a qualitative study. Maternal and child health journal. 2014;18(8). 10.1007/s10995-014-1443-x PMC4167572 25138626

[51] Elder LK. Issues in programming for maternal anaemia. Mother Care: Mother Care, 2000.

[52] Martin SL, Seim GL, Wawire S, Chapleau GM, Young SL, Dickin KL. Translating formative research findings into a behaviour change strategy to promote antenatal calcium and iron and folic acid supplementation in western Kenya. Maternal & Child Nutrition. 2017;13(1). 10.1111/mcn.12233 26898417 PMC6866120

[53] Birhanu Z, Chapleau GM, Ortolano SE, Mamo G, Martin SL, Dickin KL. Ethiopian women’s perspectives on antenatal care and iron-folic acid supplementation: Insights for translating global antenatal calcium guidelines into practice. Maternal & Child Nutrition. 2018;14(S1):e12424 10.1111/mcn.12424 29493899 PMC6866194

[54] Maina-Gathigi L, Omolo J, Wanzala P, Lindan C, Makokha A. Utilization of folic acid and iron supplementation services by pregnant women attending an antenatal clinic at a regional referral hospital in Kenya. Maternal Child Health Journal. 2013;17(7):1236–42. Epub 2012/08/22. 10.1007/s10995-012-1120-x . 22907273

[55] Gebremedhin S, Aregash S, Girma M, Tibebu M, Tsehai A. Coverage, compliance and factors associated with utilization of iron supplementation during pregnancy in eight rural districts of Ethiopia: a cross-sectional study. BMC Public Health. 2014. 10.1186/1471-2458-14-607 PMC4073172 24930036

[56] Omotayo MO, Dickin KL, Pelletier DL, Martin SL, Kung’u JK, Stoltzfus RJ. Feasibility of integrating calcium and iron–folate supplementation to prevent preeclampsia and anemia in pregnancy in primary healthcare facilities in Kenya. Maternal & Child Nutrition. 2018;14(S1):e12437 10.1111/mcn.12437 29493897 PMC6866141

[57] Nisar YB, Alam A, Aurangzeb B, Dibley MJ. Perceptions of antenatal iron-folic acid supplements in urban and rural Pakistan: a qualitative study. BMC Pregnancy and Childbirth. 2014;14:344 10.1186/1471-2393-14-344 25269515 PMC4262227

[58] Shakir F. Community Health Worker Programs: A Review of Recent Literature. USAID Health Care Improvement Project, 2010.

