# Supplementary material for: Time for change is now: Experiences of participants in a community-based approach for iron and folic acid supplementation in a rural county in Kenya, a qualitative study
Source: PLoS One. 2020 Jan 16;15(1):e0227332. doi: 10.1371/journal.pone.0227332 (PMC6964883; doi:10.1371/journal.pone.0227332)
Supplement: S1 File — This consists of three parts including: part A- Nurses’ key in-depth interview guide used to collect key information from the nurses; part B—Community health volunteers’ in-depth interview guide—the interview guide used to collect data from the community health volunteers; part C—Pregnant women’s in-depth interview guide—the interview guide used to collect data from the pregnant women. (DOCX) [file pone.0227332.s001.docx]

## APPENDIX XI A: Nurses Experiences’ Key Informant Interview Guide

**This is just a guide. Ask open-ended questions and avoid leading the clients to the answers. Explore widely to get as much information from the client as possible.**

**PART A:**

1. Share your expectations about the community-based approach of IFAS before it began
2. Compare these with your experiences throughout the conduct of the study

**PART B:** Share your experiences on this community-based distribution of IFAS through community health volunteers

1. Share your experiences with the community health volunteers in getting supplements from the hospital?
2. Share your perceptions about this approach?
3. Tell me what you liked about this approach?
4. What do you perceive as its benefits and advantages?
5. Tell me what you did not like about this approach?
6. What do you perceive as its failures and disadvantages?
7. Share the problems encountered in handling the CHVs
8. Share your challenges in using CHVs to distribute IFAS in the community
9. Compare using professional health workers and using CHVs for distribution
10. How would you want it to be done in future? Share other areas of improvement

## APPENDIX XI B: Community Health Volunteers Experiences’ Indepth Interview Guide

**This is just a guide. Ask open-ended questions and avoid leading the clients to the answers. Explore widely to get as much information from the client as possible.**

**PART A:**

1. Share your expectations about the community-based approach of IFAS before it began
2. Compare these expectations with your experiences throughout the conduct of the study

**PART B:**

1. I would like you to think of your last home visit to the pregnant women and tell me all about it
2. Share your experiences of taking IFAS tablets to pregnant women in their homes?

(Probe for:

- 1. Frequency of visits
  2. Content of visits
  3. Any counselling offered
  4. Content of counselling offered
  5. Problems encountered
  6. Perceived benefits and advantages
  7. Perceived failures and disadvantages

1. What are your perceptions about this approach now that you have been involved in it?
2. Share what you perceive as benefits and advantages of this approach?
3. Tell me what you liked about this approach?
4. Tell me what you did not like about this approach?
5. Share what you perceive as failures and disadvantages of this approach?
6. How would you want it to be done? (Probe for frequency of visits, modalities, counselling, other issues)

## APPENDIX XI C: Antenatal Mothers Experiences’ In-Depth Interview Guide

**This is just a guide. Ask open-ended questions and avoid leading the clients to the answers. Explore widely to get as much information from the client as possible.**

**PART A:**

1. Share your expectations about the community-based approach of IFAS before it began. *Nielezee matarajio yako kuhusu hii* *mbinu ya kuletewa IFAS nyumbani kabla ianze*

2. Compare these expectations with your experiences throughout the conduct of the study. *Linganisha matarajio yako na ulioyapitia katika hii mbinu ya kuletewa IFAS nyumbani*

**PART B:** I would like you to think of your last home visit from the community health volunteer and tell me all about it. *Fikiria juu ya mara ya mwisho kutembelewa* *na muhudumu wa jamii nyumbani unielezee ilivyokuwa*

1. What were your experiences with the community health volunteer bringing you the IFAS tablets at home? *Nielezee uliyoyapitia ulipokuwa unaletewa IFAS na muhudumu wa jamii nyumbani* (Probe for?
   1. Frequency of visits. *Ni mara ngapi ulitembelewa*
   2. Content of visits. *Yaliyokuwemo ulipotembelewa*
   3. Any counselling offered. *Ushauri uliopeanwa*
   4. Content of counselling offered. *Yaliyokuwemo kwa ushauri uliopeanwa*
   5. Problems encountered. *Shida zilizotokea*
   6. Perceived benefits and advantages. *Mtazamo wa umuhimu wa hii mbinu*
   7. Perceived failures and disadvantages. *Mtazamo wa ubaya wa hii mbinu*
2. What are your perceptions about this approach now that you have gone through it? *Nielezee mtazamo wako kuhusu hii mbinu ya kuletewa IFAS nyumbani*
3. Tell me what you liked about this approach? *Nielezee ni nini ulipenda kuhusu hii mbinu ya kuletewa IFAS nyumbani*
4. Tell me what you did not like about this approach? *Nielezee ni nini haukupenda kuhusu ya* *hii mbinu ya kuletewa IFAS nyumbani*
5. How would you want this approach to be conducted? *Ungependa hii mbinu itekelezwe vipi?*
