# Supplementary material for: Time for change is now: Experiences of participants in a community-based approach for iron and folic acid supplementation in a rural county in Kenya, a qualitative study
Source: PLoS One. 2020 Jan 16;15(1):e0227332. doi: 10.1371/journal.pone.0227332 (PMC6964883; doi:10.1371/journal.pone.0227332)
Supplement: S2 File — This is the main training material used for training community health volunteers. (PDF) [file pone.0227332.s002.pdf]

Nyongeza ya Iron na Folic Acid (IFAS)

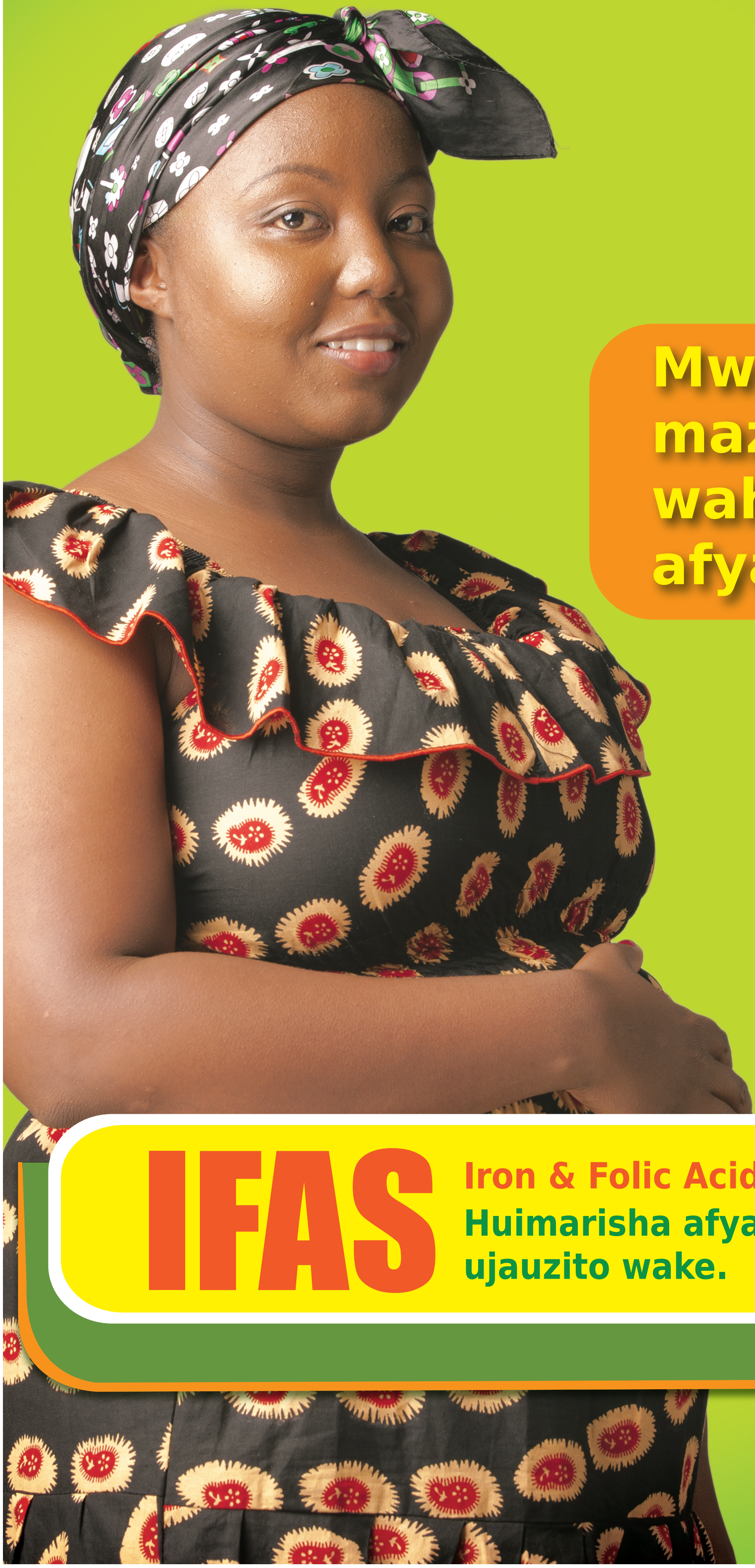

Mwongozo wa mazungumzo kwa wahudumu wa afya ya jamii.

IFAS

Iron & Folic Acid Supplements
Huimarisha afya ya mama na ujauzito wake.

Ministry of Health

Jinsi ya kutumia kadi za mazungumzo

- Kadi hizi zimeandaliwa ili kuwawezesha wahudumu wa afya ya jamii kuwasiliana vyema kuhusu nyongeza ya Iron na Folic Acid kwa akina mama wajawazito.
- Kadi hizi zaweza kutumiwa kwa ushauri nasaha wa kibinafsi au wa kikundi
- Unaweza kutumia kadi inayoambatana na ushauri nasaha unaohitajika, sio lazima kadi zote zitumiwe kwenye kila mjadala na mama mjamzito.
- Upande wa kadi ulio na michoro au picha unakusudiwa uonekane na mama, ilhali upande mwingine ulio na maandishi utatumiwa na mhudumu wa afya ya jamii.
- Ushauri nasaha wa kibinafsi kwa kina mama kuhusu Iron na Folic Acid ni muhimu kwa kuboresha matumizi .
- Hakikisha kuwa mnawasiliana moja kwa moja usoni na mama kwenye kikao cha ushauri nasaha.
- Zingatia na uyatumie yale ambayo mama tayari anaelewa.
- Yatumie maelezo muhimu kwenye kadi hizi kuhakikisha au kusahihisha maoni utakayopata kutoka kwa mama.
- Mrejelee na mama maswala muhimu mliyoyajadili ili kuhakikisha maelezo muhimu yameeleweka vyema.
- Ushauri nasaha utatofautiana ikiwa ni mara ya kwanza au ya pili mnakutana na mama. Iwapo mama amewahi kuja kwa ushauri, ni muhimu kujadili yaliyozongumziwa kwenye mjadala uliopita na kutadhmini kama ushauri ulizingatiwa na changamoto alizokumbana nazo na utoe ushauri unaostahili.

Madhumuni ya Iron na Folic Acid kwa ujauzito

Kupunguza ukosefu wa damu mwilini kwa akina mama, uwezekano wa kuzaliwa kwa mtoto aliye na uzani wa chini, ulemavu kwenye uti wa mgongo, na kuimarisha matokeo ya ujauzito kwa ujumla.

Ushauri nasaha unafaa kuhusisha yafwatayo:

Kadi ya 1: Mahitaji ya lishe bora wakati wa ujauzito
Kadi ya 2: Chanzo, dalili na madhara ya upungufu wa damu wakati wa ujauzito
Kadi ya 3: Manufaa ya Iron na Folic Acid Supplements (IFAS)
Kadi ya 4: Madhara ya kutumia Nyongeza ya Iron na Folic Acid Supplements (IFAS) na jinsi ya kukabiliana nayo
Kadi ya 5: Malaria na minyoo yaweza kusababisha ukosefu wa damu
Kadi ya 6: Wajibu wako ni upi kama mama mjamzito?

Lishe bora ni muhimu kwa ujauzito wenye afya na mtoto mwenye afya. Unahitaji kula chakula kingi na mchanganyiko wa vyakula.

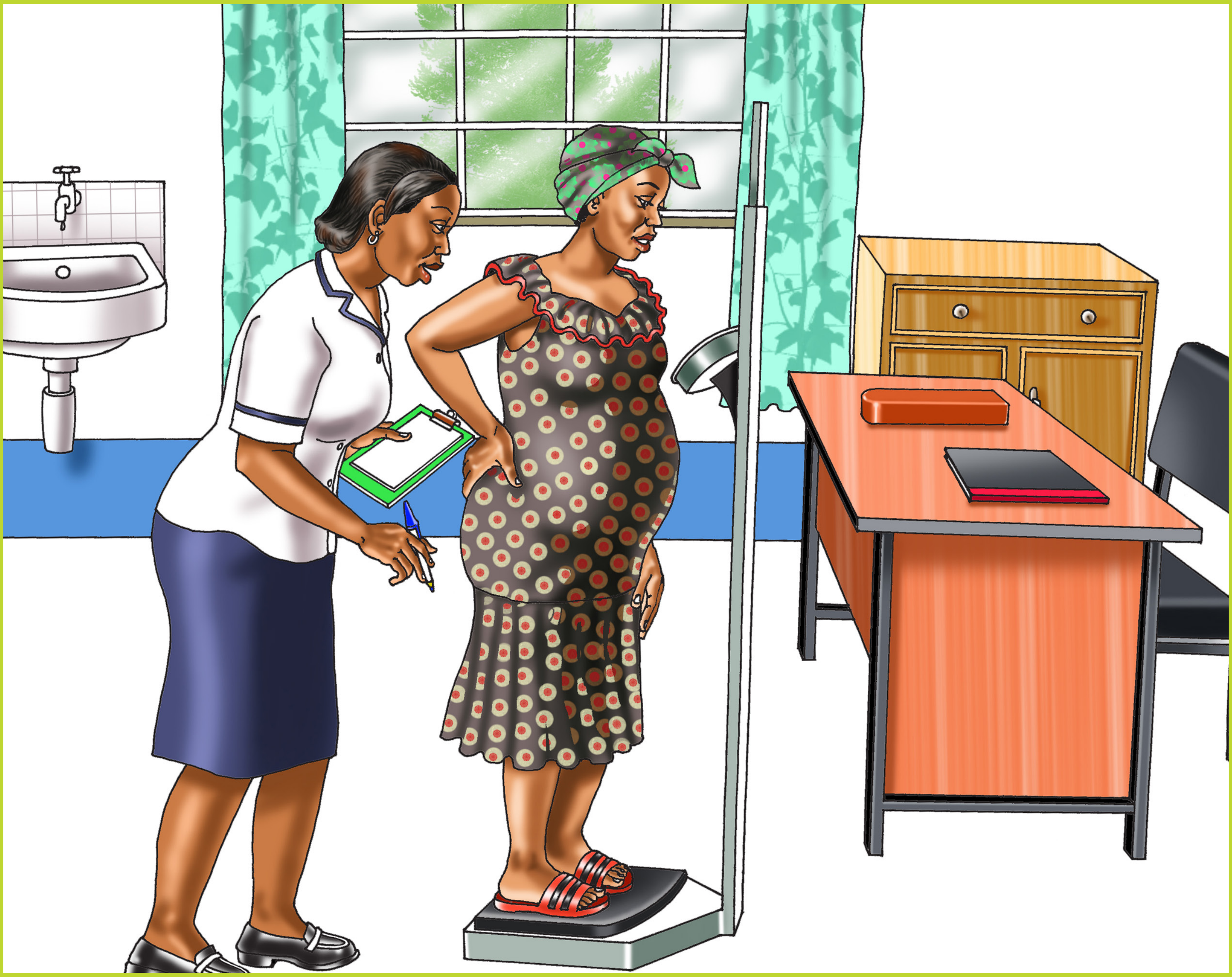

Mahitaji ya lishe bora wakati wa ujauzito.

Ujumbe muhimu: Lishe bora ni muhimu kwa ujauzito wenye afya na mtoto mwenye afya. Unahitaji kula chakula kingi na mchanganyiko wa vyakula.

Maswali ya udadisi kwa mhudumu wa afya ya jamii

- Muulize mama aeleze anachokiona kwenye picha
- Ongezea majibu ya mama na ueleze kuwa lishe bora ni muhimu kwa ujauzito
- wenye fanaka, kwa kujifungua na kumpata mtoto mwenye afya
- Eleza kuwa wakati wa ujauzito wanawake wote wanahitaji chakula zaidi na
- mchanganyiko wa vyakula unaojumuisha protini, kabohaidreti, vitamini na virutubishi
- Toa mfano wa chakula kinachopatikana kwa urahisi kwa kila kikundi cha chakula
- Muulize mifano ya mchanganyiko wa vyakula ambavyo mama anakula
- Ongezea majibu hayo na utoe mifano ya vyakula vilivyo rahisi kupatikana na vya bei nafuu vinavyoweza kuboresha lishe yake.
- Tilia mkazo hatua muhimu ambazo mama anafaa kuchukua kama ilivyoelezwa hapa chini.

Hatua Muhimu

- Kula lishe iliyo na vyakula aina tofauti kwa kutumia vyakula vinavyopatikana kwa urahisi (k.m. mbegu na nafaka kamili, mizizi na viazi, jamii ya kunde, mboga za kijani, kokwa na njugu, maziwa na bidhaa za maziwa, matunda, nyama, samaki, nyama ya kuku na mayai)
- Kula angalau mara tatu kwa siku chakula kilichokamilika na pia ule vitafunio kila baada ya saa 4 wakati wa mchana ili wewe na mtoto mpate nguvu
- Zidisha kula matunda yenye ya rangi ya kung'aa na mboga za kijani kwa sababu kutaimarisha hali ya virutubishi vingi na vitamini mwilini mwako
- Tumia bidhaa nyingi zinazotokana na wanyama kama nyama, maziwa, mayai kwa sababu ni asili nzuri ya protini, mafuta na virutubishi
- Tumia chumvi iliyotiwa madini kwenye chakula chako kwa sababu madini ya ayodini husaidia kukua kwa ubongo wa mtoto aliye tumboni.
- Pima uzani wako na uhakikishe kuwa unaongeza angalau kilo moja kila mwezi hasa katika kipindi cha pili na cha tatu cha ujauzito.
- Punguza kiwango cha kazi zako na upumzike zaidi wakati wa ujauzito ili kuhifadhi nguvu.

Upungufu wa damu mwilini unaweza kusababisha kuongezeka kwa vifo wakati wa kujifungua na vifo vya watoto kabla ya kuzaliwa.

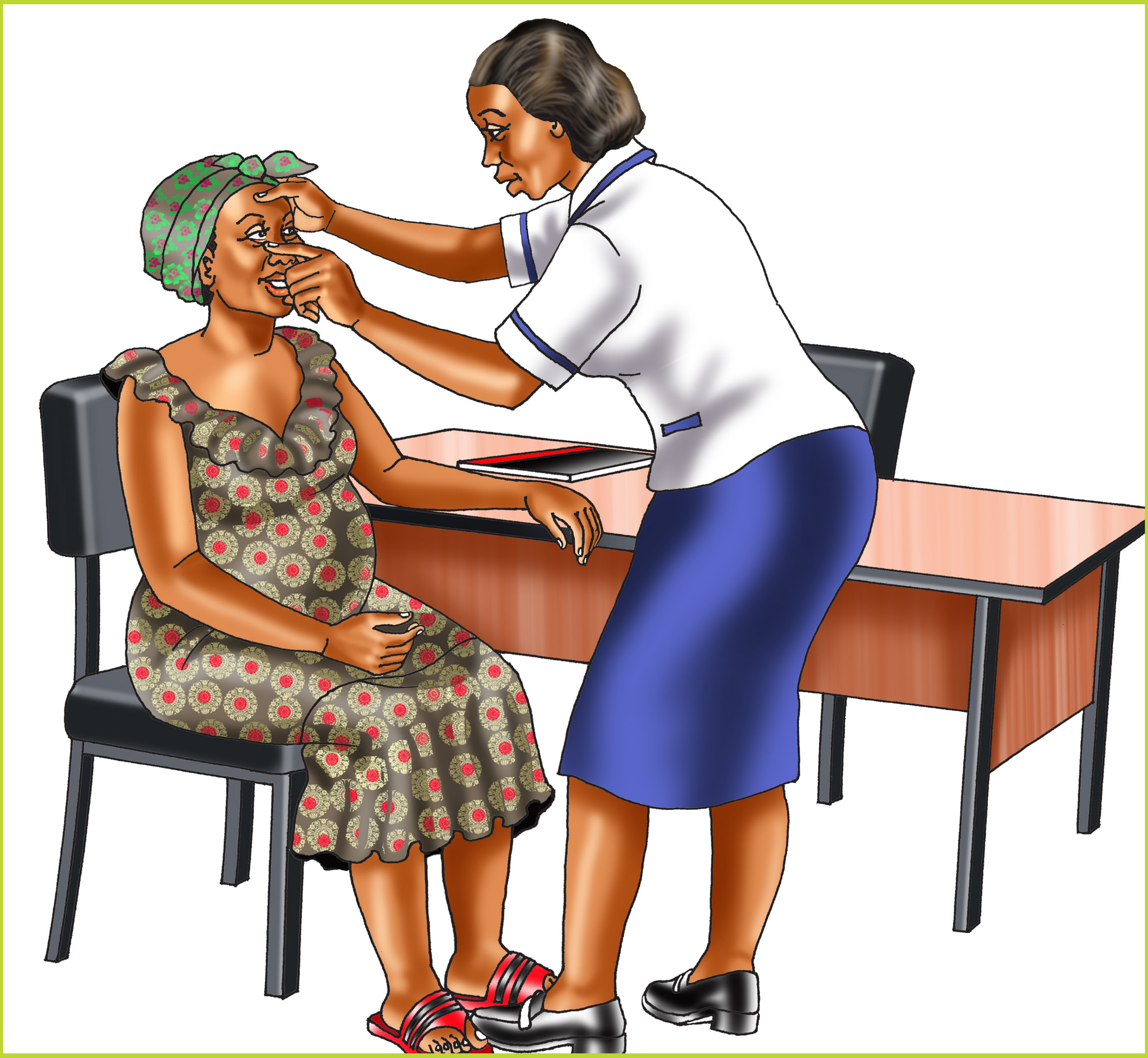

Sababu, dalili na madhara ya upungufu wa damu wakati wa ujauzito.

Ujumbe muhimu: Upungufu wa damu mwilini unaweza kusababisha kuongezeka kwa vifo wakati wa kujifungua na vifo vya watoto kabla ya kuzaliwa

Maswali ya kudadisi kwa mhudumu wa afya ya jamii

- Muulize mama aeleze anachokiona kwenye picha
- Ongezea kwenye majibu ya mama na ueleze kuwa upungufu wa damu wakati wa ujauzito ni hatari, unaweza kusababisha uzani wa chini wa mtoto anapozaliwa na ambayo ndiyo sababu kubwa ya vifo vya watoto wachanga na wale walioko tumboni mwa mama
- Eleza sababu za kawaida, dalili na madhara ya upungufu wa damu wakati wa ujauzito kama zilivyoelezwa hapa chini;

Sababu, dalili na madhara ya upungufu wa damu mwilini

- **Sababu ya kawaida ya upungufu wa damu ni pamoja na;** kutokula vyakula vyenye iron kama nyama, ini, kuku, kuwa na maradhi kama malaria, kuwa na minyoo, virusi vya ukimwi, kuendesha na magonjwa mengine.
- **Dalili za kawaida za upungufu wa damu ni pamoja na:** udhaifu, kutoweza kupumua vyema, kisunzi, kuumwa na kichwa, uchovu, kuchujuka rangi kwa kope za macho na viganja.
- **Madhara ya upungufu wa damu ni kama vile:** uzani wa chini wakati wa kujifungua ambao waweza sababisha vifo vya watoto wanaozaliwa na walioko tumboni mwa mama, kuongezeka kwa matatizo ya wakati wa kujifungua

Maswali ya kufuatilia kwa mteja

- Muulize mama kama ana maswali yoyote yanayohusu sababu, dalili na madhara ya upungufu wa damu mwilini
- Muulize kama amepata mojawapo ya dalili hizo
- Mfafanulie wazi umuhimu wa kwenda kwenye kliniki ya kinamama wajawazito (ANC) kila mwezi kwa ukaguzi wa kila mara pamoja na ukaguzi wa hali ya damu

Iron na Folic Acid Supplements (IFAS) hukuhakikishia ujauzito wenye afya na mtoto mwenye afya.

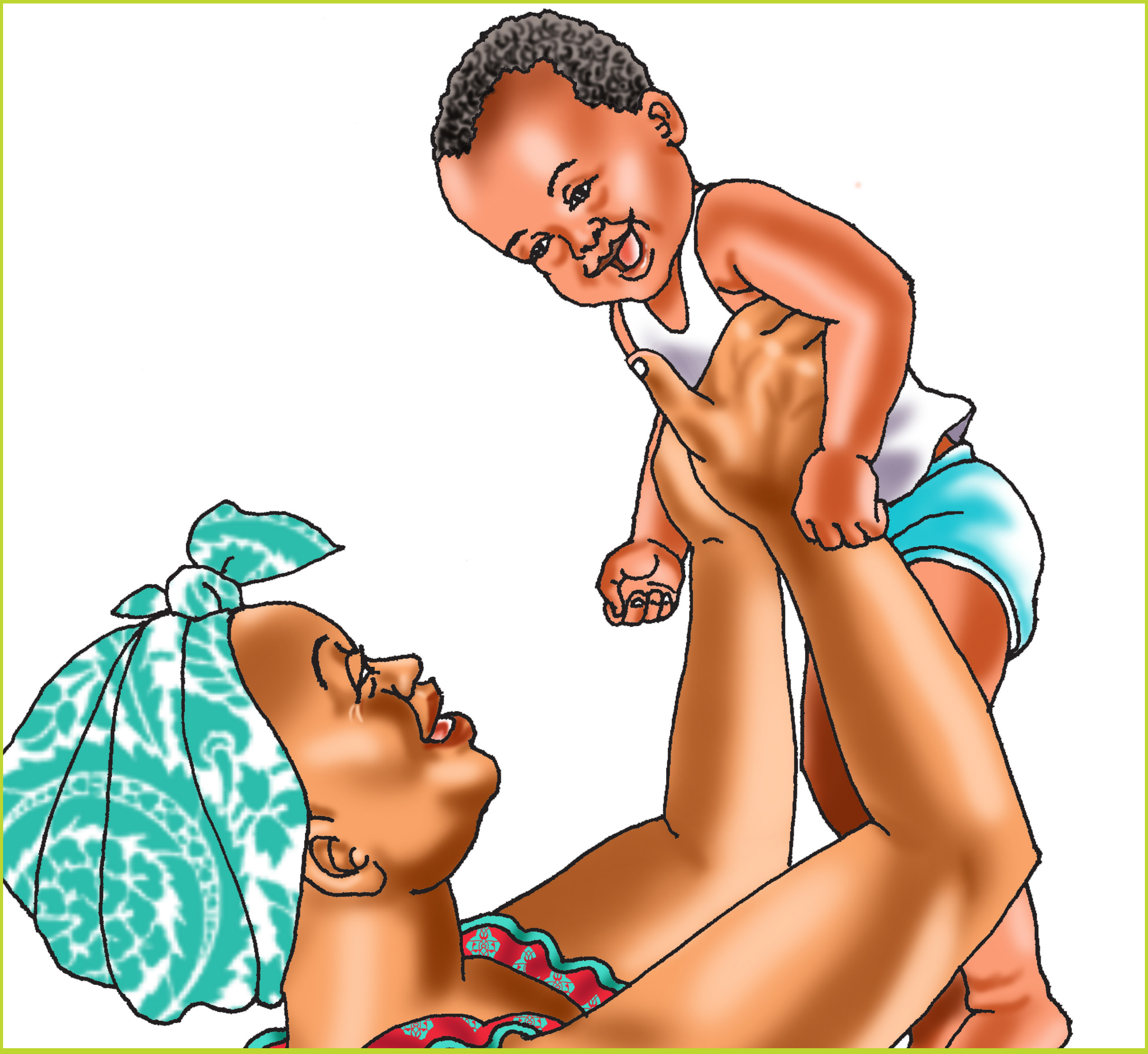

Manufaa ya Iron na Folic Acid Supplements (IFAS).

Ujumbe muhimu: : Iron na Folic Acid Supplements (IFAS) hukuhakikishia ujauzito wenye afya na mtoto mwenye afya.

Maswali ya kudadisi kwa mhudumu wa afya ya jamii

- Muulize mama aeleze anachokiona kwenye picha
- Muulize mama kama amewahi kutumia IFAS au anatumia IFAS
- Ongezea kwenye majibu ya mama na umweleze kuwa mahitaji ya madini ya iron na folic acid wakati wa ujauzito ni vigumu kutimiza kwa kutumia aina nyingi za vyakula.
- Eleza kuwa akina mama wote wajawazito wanafaa kupewa IFAS bila kujali viwango kiwango cha damu mwilini, na wanatakiwa kumeza tembe moja kila siku wakati wa ujauzito
- Eleza manufaa muhimu ya IFAS yaliyoelezwa hapa chini:

Manufaa ya tembe ya IFAS iliyochanganywa

- Tembe chache humezwa zikilinganishwa na tembe za iron na folic acid zikiwa kando
- Huimarisha uzingatifu ikilinganishwa na tembe zikiwa tofauti
- Wakati mwingine tembe hupakwa sukari na hivyo kuwa na ladha tamu

Manufaa ya Iron

- Huzuia upungufu wa damu mwilini
- Hupunguza hatari ya uzani mdogo wa mtoto anapozaliwa
- Huhifadhi nguvu wakati wa ujauzito
- Huhakikisha kuwa mama ana akiba ya kutosha ya damu wakati na baada ya kujifungua

Manufaa ya Folic Acid

- Hupunguza visa vya ulemavu kwa uti wa mgongo inapotumiwa kabla ya kupata mimba na kipindi cha siku 28 baada ya kupata mimba
- Wakati wa ujauzito husaidia mwili kutumia iron na kupunguza upungufu wa damu

Maswali fuatilizi kwa mama

Muulize kama ana maswali yoyote kuhusu manufaa ya IFAS iliyochanganywa. Tumia kadi inayofuata kujadili madhara ya matumizi na jinsi ya kuyadhibiti.

Tumia Iron na Folic Acid Supplements (IFAS) pamoja na vyakula kupunguza uwezekano wa kupata kichefuchefu.

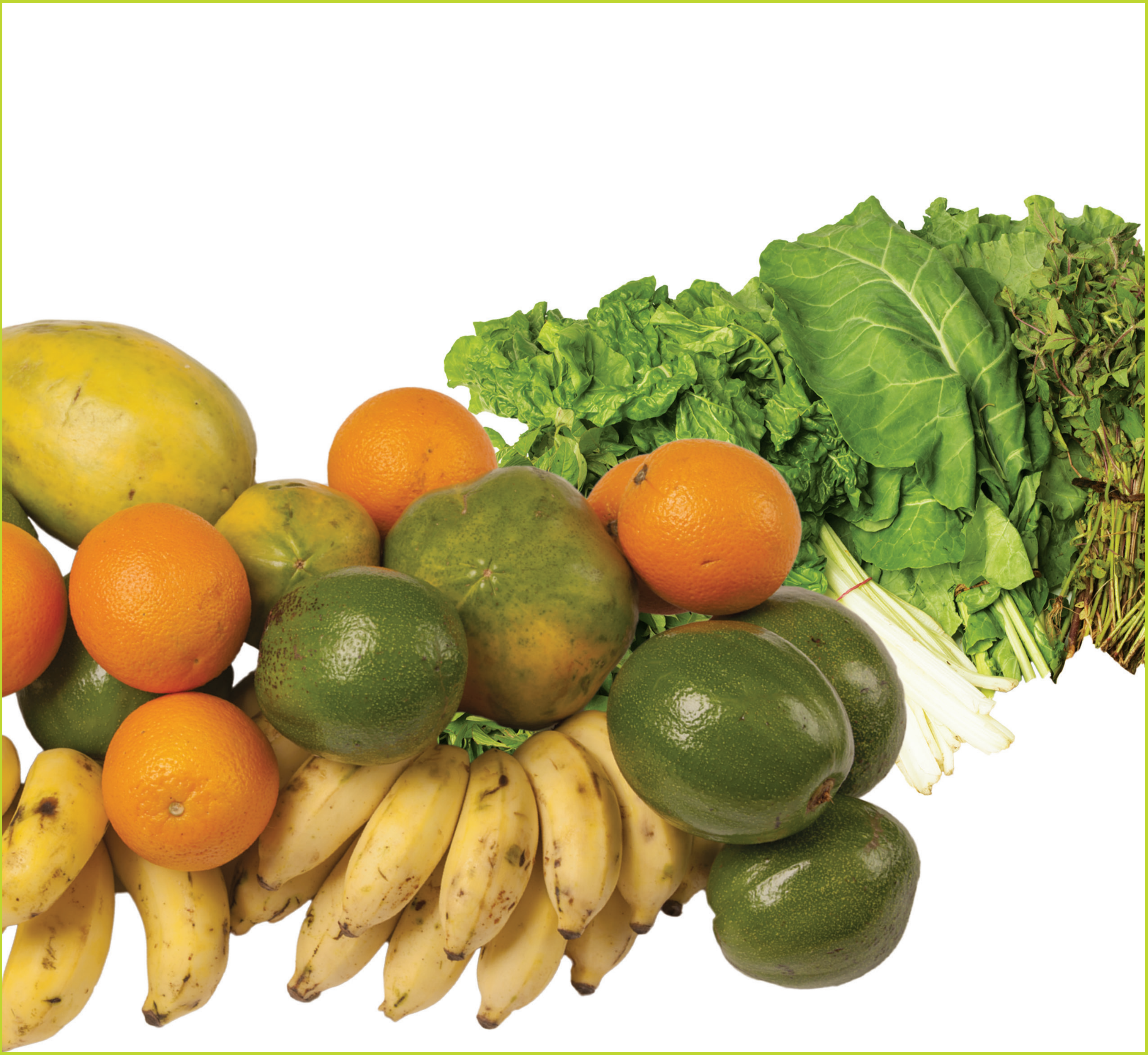

Athari za Iron na Folic Acid Supplements (IFAS) na Jinsi ya Kuzidhibiti

Ujumbe muhimu: Meza Iron na Folic Acid Supplements (IFAS) wakati wa mlo ili kupunguza uwezekano wa kupata kichefuchefu.

Maswali ya kudadisi ya mhudumu wa afya ya jamii

- Muulize mama kama amewahi kupatwa na athari za IFAS na muulize azitaje
- Muulize jinsi alivyoweza kuzidhibiti athari hizo ikiwa alizipata
- Kwa kutumia athari hizo mweleze mama kwamba ni kawaida kwa athari kama hizo kutokea anapotumia IFAS
- Eleza kwamba athari hizo sio mbaya na kuwa nyongeza hiyo ya IFAS inafaa kuendelea
- Eleza athari zinazoweza kutokea na njia zinazopendekezwa za kukabiliana nazo kulingana na jedwali lililo hapa chini:

Athari za kawaida na njia za kuzidhibiti

Athari zinazoweza kutokea	Hatua za kuchukua
Maumivu ya tumbo, kichefuchefu, kuendesha au kuvimbiwa	• Epuka kutumia kiasi kikubwa cha Vitamini C supplement, pamoja na vidonge vya IFAS.<br>• Kula matunda na mboga kwa wingi. Meza IFAS wakati wa kula chakula
Kinyesi kugeuka rangi na kuwa cheusi kutokana na kukosa kufyonzwa kwa madini ya chuma.	Kubadilika kwa rangi ya kinyesi ni kawaida na matumizi ya IFAS yanafaa kuendelea.

Swali la kufuatilia hali ya mama mjamzito

Muulize kama anayo maswali kuhusu athari. Tumia kadi inayofuata kumshauri mama kuhusu upungufu wa damu mwilini na mikakati ya kukingana nayo.

Lala ndani ya neti usiku na utumie dawa ya minyoo mara kwa mara.

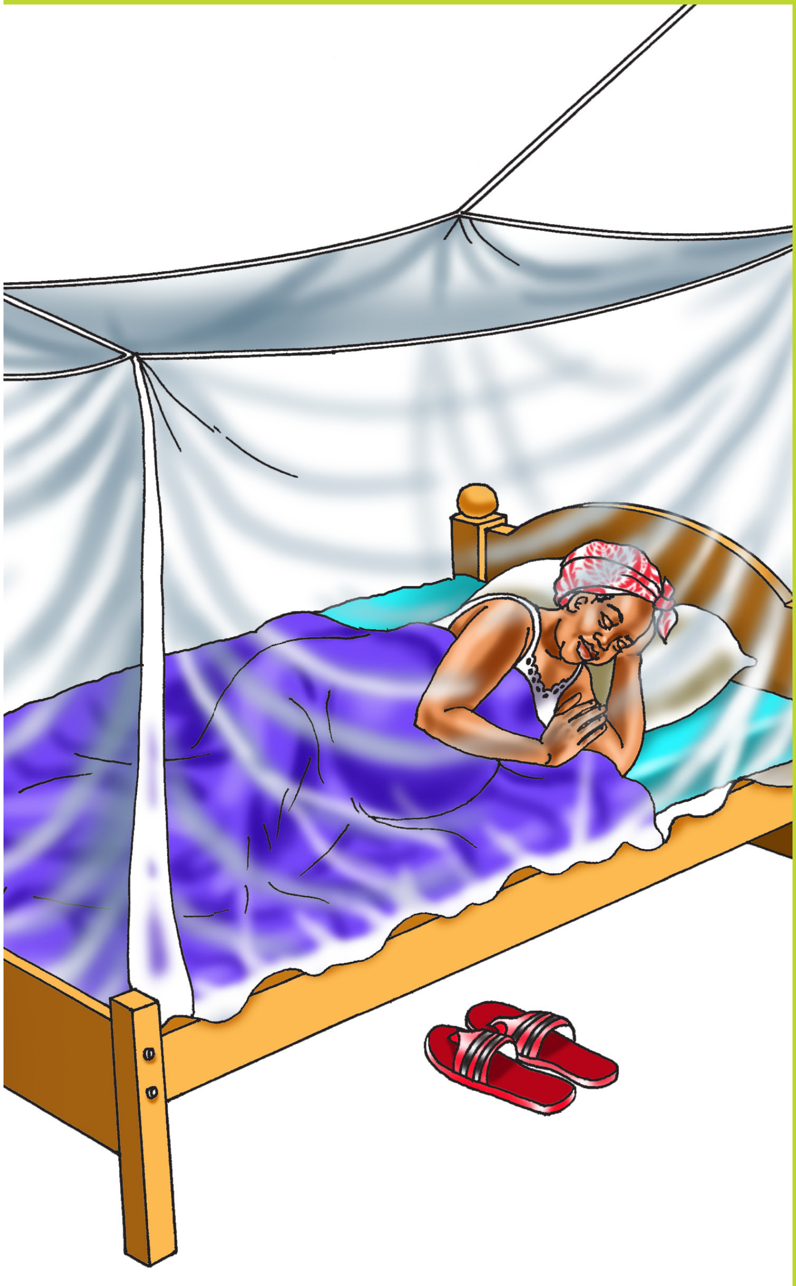

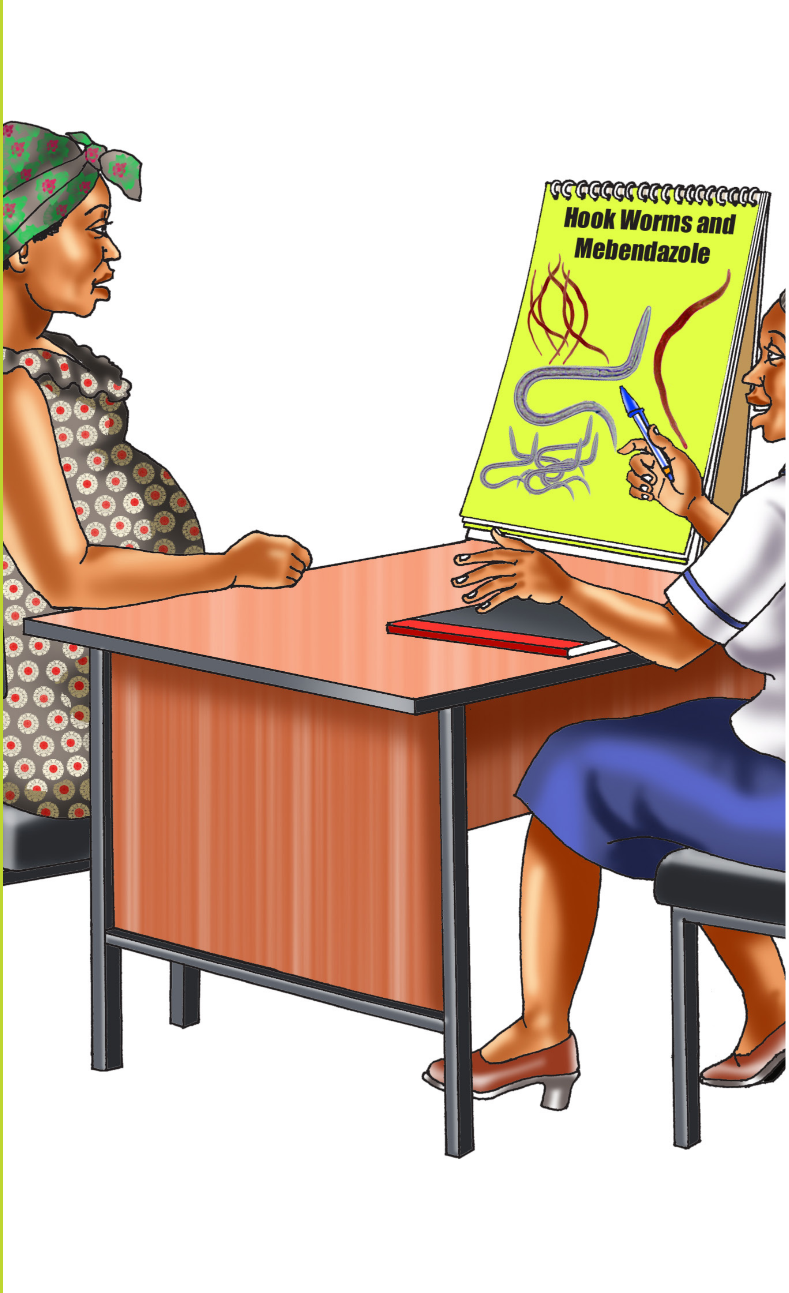

Ugonjwa wa malaria na minyoo sugu yaweza kusababisha upungufu wa damu mwilini.

Ujumbe muhimu: Lala ndani ya neti kila usiku na utumie dawa ya minyoo mara kwa mara.

Maswali ya kudadisi kwa mhudumu wa afya

- Muulize mama mjamzito anachokiona kwenye picha
- Kulingana na maelezo yake fafanua zaidi jinsi kinga ya malaria na udhibiti wake vilivyo muhimu katika kuzuia upungufu wa damu mwilini wakati wa ujauzito. Kuna uhusiano mkubwa kati ya malaria wakati wa ujauzito na upungufu wa damu mwilini
- Eleza kuwa akina mama wajawazito katika maeneo yaliyo na malaria ni lazima watumie dawa kuwakinga na malaria za IPT wakati huo wa ujauzito hata iwapo hawataonyesha dalili za kuwa na malaria na hata kama kiwango cha damu yao ni nzuri
- Pia eleza kuwa minyoo sugu yaweza kumfanya mama mjamzito kudhoofika kiafya, apungukiwe na damu mwilini na kuwa urahisi wa kuambukizwa magonjwa. Kuambukizwa kunatokana na kula chochote kilichogusa udongo ambao umesibikwa na kinyesi cha mwanadamu
- Imarisha hatua muhimu za kuchukuliwa na mama kama ilivyoelezwa hapo chini:

Ujumbe muhimu

- Hakikisha kwamba unatembelea kliniki ya ANC kila mwezi katika kipindi chote cha ujauzito wako
- Hakikisha kwamba unapata dawa za kuzuia malaria (IPT) kutoka katika kliniki ya ANC hata kama huna dalili zozote mwilini za malaria na damu yako iko sawa
- Uliza upewe neti iliyotiwa dawa ya kuulia wadudu na ulale ndani yake kila siku
- Hakikisha umezingatia viwango vya juu vya afya na mazingira na unatumia dawa za minyoo ambazo zinatolewa bure katika vituo vya afya

Swali fuatilizi ya hali ya mama mjamzito

Muulize kama ana maswali yoyote kuhusu malaria na maambukizi ya safura (minyoo)
Mshauri mama wakati wowote kutafuta matibabu katika kituo chochote cha afya.

Ukiwa mama mjamzito ni jukumu lako kuchukua hatua za kuhakikisha kuwa afya yako na ya mtoto wako aliye tumboni ni thabiti.

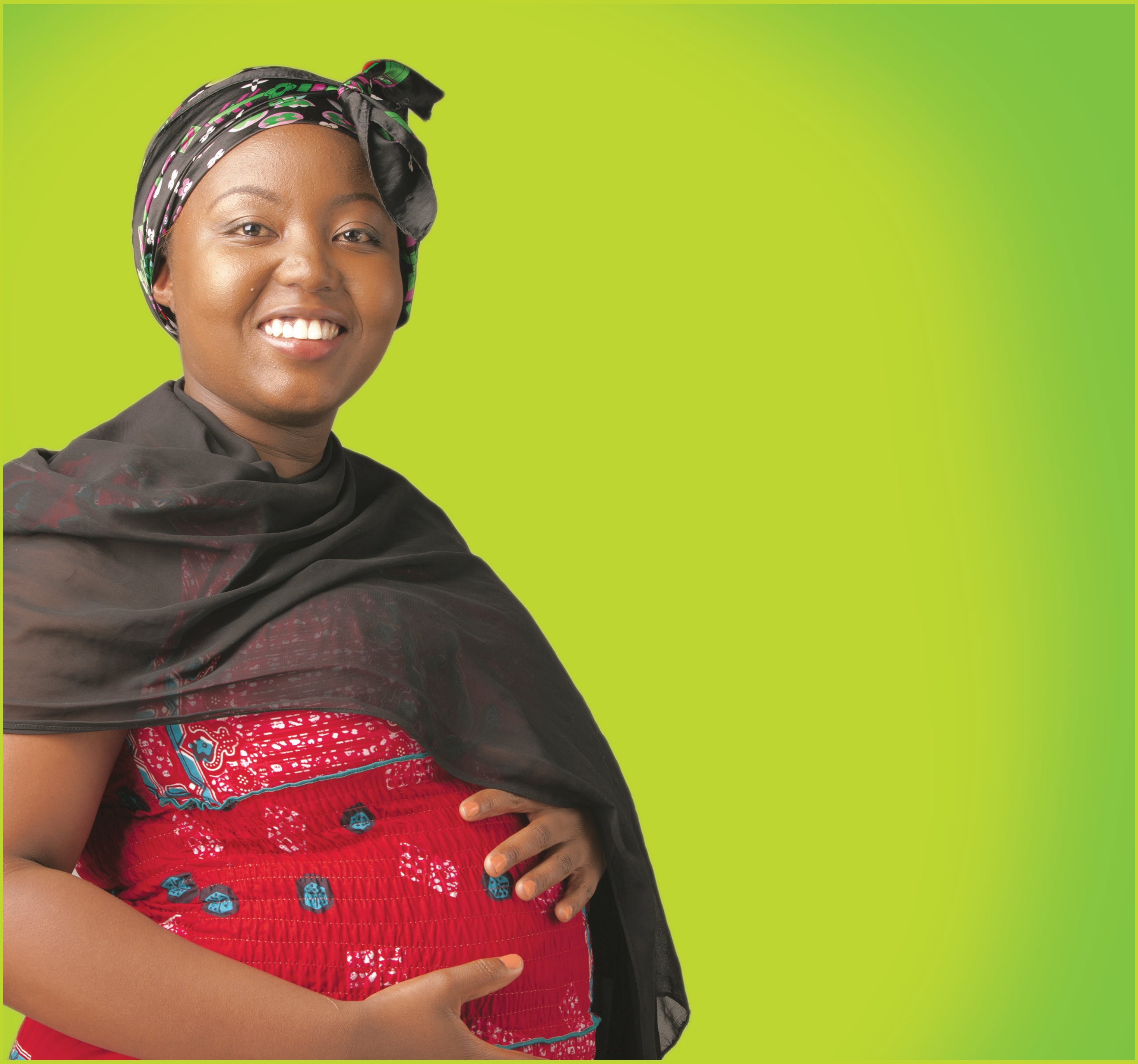

Jukumu lako kama mama mjamzito ni lipi?

Ujumbe muhimu: Ukiwa mama mjamzito ni jukumu lako kuchukua hatua za kuhakikisha kuwa afya yako na ya mtoto wako aliye tumboni ni thabiti.

Maswali ya kudadisi kwa mhudumu wa afya ya jamii

- Muulize mama mjamzito kueleza hatua muhimu za kuchukua ili kufanikisha afya bora katika ujauzito
- Zingatia majibu ya mama na tilia mkazo hatua zilizotajwa hapa chini;

Hatua muhimu

- Tembelea kliniki ya ANC iliyokaribu mara tu unapojua kwamba u mjamzito
- Hudhuria kliniki za ANC kila mwezi
- Tumia IFAS kila siku katika kipindi cha ujauzito
- Kula mlo uliokamilika wa vyakula vyenye Iron kwa wingi
- Kula aina tofauti ya vyakula vinavyopatikana kwa urahisi
- Lala ndani ya neti kila siku
- Tumia dawa ya minyoo mara kwa mara
- Ungana na vikundi vya kusaidiana vya kinamama

IFAS

Iron & Folic Acid Supplements

Huimarisha afya ya mama na ujauzito wake.

Ministry of Health
