# Supplementary material for: Time for change is now: Experiences of participants in a community-based approach for iron and folic acid supplementation in a rural county in Kenya, a qualitative study
Source: PLoS One. 2020 Jan 16;15(1):e0227332. doi: 10.1371/journal.pone.0227332 (PMC6964883; doi:10.1371/journal.pone.0227332)
Supplement: S3 File — This is one of the training materials used for training the community health volunteers. (PDF) [file pone.0227332.s003.pdf]

HARAMBEE

GOVERNMENT OF KENYA

MINISTRY OF HEALTH

DIVISION OF NUTRITION

ACCELERATING REDUCTION OF IRON DEFICIENCY ANAEMIA AMONG PREGNANT WOMEN IN KENYA

PLAN OF ACTION

2012-2017

GOVERNMENT OF KENYA

MINISTRY OF HEALTH

DIVISION OF NUTRITION

ACCELERATING REDUCTION OF IRON DEFICIENCY ANAEMIA AMONG PREGNANT WOMEN IN KENYA

PLAN OF ACTION

2012-2017

ACKNOWLEDGEMENTS

The Division of Nutrition appreciates the tireless efforts of members of the task force through the supplementation sub-committee for their valuable inputs in development of this Plan of Action. The task force was comprised of members from Division of Nutrition (DoN), Division of Child and Adolescent Health, Division of Reproductive Health, in the Ministry of Health (MoH), USAID-MCHIP, Kenyatta University, UNICEF (KCO), and Micronutrient Initiative (MI).

We would like to express our gratitude to USAID-MCHIP for their Technical and Financial support. Special thanks go to Evelyn Kikechi (DON), Evelyn Matiri (USAID-MCHIP), Marjorie Volege (UNICEF –KCO), Ruth Situma (UNICEF KCO) and Esther Wamae (MI) for their contributions and support for technical processes in developing this strategy. The ministry also acknowledges the support and contributions of all national staff of the Division of Nutrition for their contributions.

Last but not least, appreciations go to the Permanent secretary Mr. Mark Bor, the Director of Public Health and Sanitation, Dr, Sharif, Head, Department of Family Health, Dr. Wamae for their invaluable support and contributions that enabled us to accomplish this task.

Terrie Wefwafwa HSC

Head, Division of Nutrition

FOREWORD

With three years left to the Millennium Development Goals (MDG) deadline, many countries, Kenya included, are far from achieving the targets set almost ten years ago. Kenya has made insufficient progress towards achieving these goals especially MDG 4 and 5 with a slow decline in child mortalities and stagnation of maternal mortality (KDHS, 2008-09). At the current rates, the under 5 mortality and maternal mortality are respectively 2.3 and 3.3 times higher than the MDG targets.

Iron and Folic acid (IFA) supplementation for pregnant women has been shown to reduce maternal anaemia and consequently maternal mortality and LBW while folic acid if taken from the time of pre-conception and throughout the early months of pregnancy is critical in preventing neural-tube birth defects. Whereas these high impact interventions are known, their uptake is low and therefore children and women do not optimally benefit from them. Various factors contribute to these low uptake levels and hence to Kenya's slow progress in addressing MDG 4 and 5. These factors need to be well addressed effectively to help achieve the MDG targets.

IFA deficiency control is prioritized in the National Food and Nutrition Security Policy and the draft National Health Sector Strategic Plan (NHSSP III). The National Nutrition Action plan 2012 - 2017 identifies four main strategies to successfully address iron and folic acid deficiencies among pregnant women. These are 1) dietary diversification and modification 2) food fortification and bio-fortification, 3) iron and folic acid supplementation and 4) public health measures including malaria control and helminthes control. Other public health strategies to control iron and folic acid deficiencies are also highlighted in the Food and Nutrition Security Policy which takes cognizance of other sectors that contribute to reducing micronutrient deficiencies.

While the main focus of this document is on iron and folic acid supplementation programs, this plan of action acknowledges the beneficial role parasite control, food fortification and dietary diversification and malaria control programs can have in controlling iron deficiency anemia.

Addressing iron and folic acid deficiency requires clear policies, strategies, actions and investment. While the policies are in place, this plan of action seeks to provide the practical implementation plan with clear actions and costing for the next five years. The plan of action document has been developed by the Division of Nutrition within the Ministry of Health to coordinate all relevant stakeholder efforts on the priority actions that the country will focus on, for the next 5 years, to strengthen the IFA programme to improve coverage and utilization of iron and folic acid supplements thereby preventing and controlling the iron deficiency anemia among pregnant women. The plan of action will be useful for stakeholders who are involved in designing, implementing and evaluating IFA programs at national, county and community levels.

Dr. Annah Wamae OGW

Head, Department of Family Health

TABLE OF CONTENTS

LIST OF ACRONYMS 5

EXECUTIVE SUMMARY 6

1.1 SITUATION ANALYSIS OF MATERNAL ANAEMIA IN KENYA 7

1.2 INTERVENTIONS TO ADDRESS ANAEMIA AMONG PREGNANT WOMEN 7

1.3 CHALLENGES CURRENTLY FACED IN IFA SUPPLEMENTATION 7

2.0 PROPOSED PLAN TO ADDRESS BARRIERS OF IFA SUPPLEMENTATION 10

2.1 PURPOSE AND OBJECTIVES OF PLAN 10

2.2 PROCESS OF DEVELOPING PLAN 10

Table 1: IFA Supplementation Plan: Focus Areas and Expected Outcomes 11

Table 2: IFA Supplementation Plan: Implementation Framework and Summary Budget .... for the period 2012-2017 12

ANNEX 1: DETAILED BUDGET 17

ANNEX 2: LIST OF CONTRIBUTORS 25

REFERENCES 26

LIST OF ACRONYMS

ACSM	Advocacy, Communication and Social Mobilization
ANC	Ante Natal Care
CHW	Community Health Workers
CME	Continuous Medical Education
DCHS	Division of Child Health Services
F&Q	Forecasting and Quantification
FANC	Focused Ante Natal Care
FAO	Food and Agriculture Organization
FSNP	Food Security and Nutrition Policy
HIS	Health Information System
HIV	Human Immuno Virus
HMIS	Health Management Information System
HW	Health Workers
IDA	Iron Deficiency Anaemia
IEC	Information, Education and Communication
IFA	Iron and Folic Acid
IRCK	Inter Religious Council of Kenya
KAP	Knowledge, Attitude and Practices
KDHS	Kenya Demographic Health Survey
KEMSA	Kenya Medical Supplies Agency
KSPA	Kenya Service Provision Assessment
LBW	Low Birth Weight
M&E	Monitoring and Evaluation
MCH	Maternal and Child Health
MCHIP	Maternal and Child Health Integrated Program
MI	Micronutrient Initiative
MIYCN	Maternal, Infant and Young Child Nutrition
MOA	Ministry of Agriculture
MOMS	Ministry of Medical Services
MOPHS	Ministry of Public Health and Sanitation
MTEF	Mid Term Expenditure Framework
NMDCC	National Micronutrient Deficiency Control Council
OJT	On Job Training
PNC	Post Natal Care
PS	Permanent Secretary
SCUK	Save the Children UK
SUN	Scaling Up Nutrition
TBD	To Be Done
TOR	Terms of Reference
UNICEF	United Nations Children's Funds
WFP	World Food Programme
WHO	World Health Organization

EXECUTIVE SUMMARY

The National IFA Supplementation Plan of Action 2012-2017 was formulated and developed through extensive participatory collaborations and consultations between the Ministry of Public Health and Sanitation, NGOs, partners and other relevant stakeholders. The process was coordinated by the Division of Nutrition though the National Supplementation Sub-Committee, the National Micronutrient Deficiency Control Council and the Nutrition Interagency Coordinating Committee. The main objective of this document is to provide a framework for coordination of stakeholder efforts towards strengthening the IFA programme implementation to improve the coverage and utilization of iron and folic acid supplements among pregnant women, thereby contributing to the reduction of iron deficiency anaemia.

Implementing the costed Plan of Action will require increased political will, donor investment, stakeholder involvement, public investment and a heightened awareness of the critical importance of IFA supplementation among health workers, pregnant women and community based care providers. Involvement of the national and county government, families, communities, community based organizations (CBOs), in collaboration with international organizations and other concerned parties will ultimately ensure that necessary action is taken.

The Costed Plan of Action is divided into eight broad areas as follows:

- **Chapter One:** This section provides an insight into the status of maternal anaemia in Kenya, the interventions in place to address anaemia among pregnant women and the challenges currently faced in IFA supplementation,

- **Chapter Two:** Proposed Plan to Address Barriers of IFA Supplementation - this section provides the objectives of the Plan of Action 2012-2017 and also a 5 year costed IFA Supplementation Plan with 5 key Focus Areas and their expected outcomes.

CHAPTER I

1.1 Situation Analysis of Maternal Anaemia in Kenya

The prevalence of anaemia among pregnant and lactating women in Kenya is worrying. The Kenya Micronutrient Survey, 1999 indicated the prevalence of iron-deficiency anaemia among pregnant women to be high at 55.1% and 46.4% among non-pregnant women. Anaemia, resulting from iron deficiency, referred to as iron deficiency anaemia, is the most common type of anaemia globally. The risk factors of iron deficiency anaemia (IDA) include: inadequate consumption or low intake of haeme iron, consumption of staples with low bio available iron, inadequate intake of foods that enhance iron absorption from diet such as vitamin C, consumption of foods high in inhibitors of iron absorption, parasitic infection, malaria, chronic infections, heavy blood loss and restricted food intake.

The consequences of anaemia are major. Maternal anaemia contributes to maternal and peri-natal mortality, pre-term delivery, low birth weight (LBW) and fetal impairment. Anaemia is associated with an increased risk of morbidity and mortality, especially in pregnant women and young children. Maternal deaths in Kenya have been on the increase as evidenced in the last two demographic health surveys; 488 maternal deaths per 100,000 live births were recorded in 2008-09 compared with 414 deaths per 100,000 live births in 2003. Neonatal deaths were at 31 per 1,000 live births in 2008-09 and they accounted for approximately 60 percent of under-five mortality.

1.2 Interventions to address Anaemia among Pregnant Women

Globally and in Kenya, one of the key strategies that has been used for years to address anaemia among pregnant women is iron and folic acid (IFA) supplementation. IFA supplementation is critical as it has been shown to reduce maternal anaemia and consequently maternal mortality and LBW while folic acid is critical in preventing neural-tube birth defects. IFA supplementation for pregnant women in the country is one of the routine services provided within Focused Antenatal Care [FANC].

It is important to note that the IFA supplementation is only one of the maternal anaemia control strategies. It is clear from experience that implementation of a package of proven interventions are much more likely to succeed in improving maternal anaemia than implementing any one single intervention. Other interventions critical in dealing with maternal anaemia include fortification of food with iron, malaria control, HIV management, hookworm control and optimal birth spacing as appropriate. For the coordinated integration of all these strategies, there is need for strong collaboration with key divisions including Divisions of Nutrition (DON), Reproductive Health (DRH), Malaria and Environmental Health.

1.3 Challenges currently faced in IFA supplementation

1.3.1 Compliance

As indicated in the Kenya Demographic Health Survey (KDHS) 2008-09, IFA compliance is poor with only 2.5% of women taking supplements for ≥90 days. Over 30% women do not take any iron supplements during pregnancy. A comparison with 2003 KDHS data indicates that the proportion of women who took iron supplements increased from 41% to 60% in 2008-09. Although this is a sizeable increase, almost all of the women who took iron supplements took them for less than 60 days during pregnancy.

FIGURE 1: Distribution of women who took iron tablets or syrup during pregnancy of last birth by number of days (KDHS 2008)

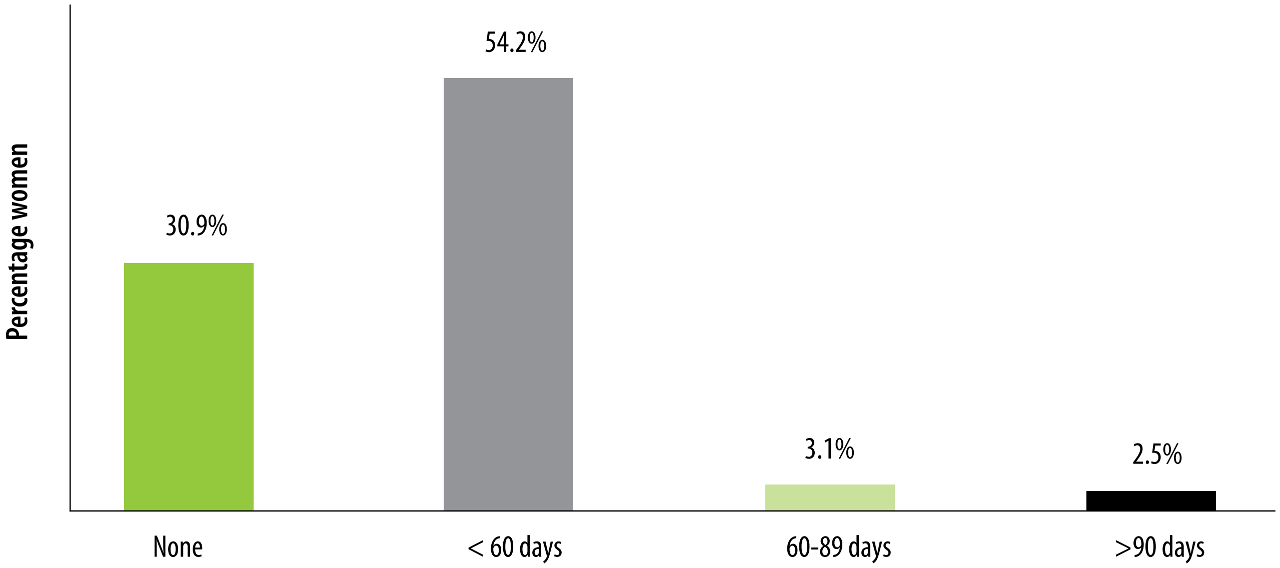

1.3.2 Counselling skills and Awareness

The KSPA indicated that there were limited pre- and in-service training on FANC and this compromised quality of counselling at the Ante Natal Care (ANC) and Postnatal Care (PNC).

1.3.3 Late start of antenatal care by pregnant women

IFA supplementation is dependent on client adherence to recommended number and timing of ANC visits. The KDHS 2008-09 currently indicates that 47% of all pregnant women meet the recommended four or more ANC visits. Provider capability to provide the range of services required for focused ANC remains weak in some aspects and most providers have limited knowledge of which routine services are expected for FANC, including critical aspects like the timing of visits, and many aspects of education and counseling. It is worth noting that the training curricula for ANC in pre-service training institutions have remained unchanged, a situation that gravely affects the quality and sustainability of focused ANC services, including IFA supplementation.

1.3.4 Supply Chain

IFA tablets are part of the essential drugs list procured and distributed by government to all health facilities. The procured iron supplements are used both for treatment and supplementation in the health facility, a situation which sometimes results in shortages of IFA supplements for use during the pregnancy period. Iron supplementation program for pregnant women through public health system had many challenges amongst them poor stock control management leading to breakage of supply chain and collapse of the program during the last decade. Data from the Kenya Service Provision Assessment (KSPA) 2012 showed that only 41% of health facilities had iron tablets, while 74% had folic acid supplements. A thorough analysis of the FANC supply chain system is necessary to understand all other factors affecting availability of stocks within health facilities.

1.3.5 Formulation

The current IFA supplementation recommendations for pregnant women are detailed in the Kenya National Technical Guidelines for Micronutrient Deficiency. The recommended dosage per day is 60mg iron and 400µg folic acid, and the period for supplementation is from the first month of pregnancy for a period of 6 months.

The Ministry of Health (MOH) has recently introduced enteric coated and combined formulations (60mg Fe and 400µg FA). This formulation is recommended to replace the high dose iron supplements (200mg that are currently being used which have been associated with side effects such as constipation and other gastrointestinal effects including nausea, vomiting and diarrhoea. The new formulations will reduce the side effects experienced by women and will be important to the country because they are likely to be more acceptable resulting in increased compliance levels. The combined formulations will also be easier to take than separate iron and folic supplements.

1.3.6 Weak implementation strategy

National guidelines indicate that iron tablets and folic acid tablets are to be provided to all pregnant women attending ANC at the Maternal and Child Health (MCH) clinics in all health facilities (levels 2-5). Due to lack of clarity, it has been found that health workers insist on first screening pregnant women for anaemia before prescribing IFA tablets.

For IFA supplementation to have the desired impact on the iron and health status of pregnant women and newborns, it is necessary to strengthen the implementation of the intervention.

CHAPTER 2

2.0 Proposed Plan to Address Barriers of IFA Supplementation

2.1 Purpose and Objectives of Plan

The Ministry of Health (MOH), Division of Nutrition with support from partners has embarked on a robust five year plan aimed at accelerating reduction of anaemia among pregnant women through strengthening IFA supplementation.

The main objective of this plan is to coordinate stakeholder efforts towards strengthening the IFA programme to improve the coverage and utilization of iron and folic acid supplements among pregnant women, thereby contributing to the reduction of iron deficiency anaemia.

The specific objectives of the plan are:

1. To improve the knowledge, attitudes and practices on IFA supplementation among pregnant women, health workers and other key influencers

2. To ensure quality and timely implementation of evidence-based IFA interventions

3. Increase coverage of IFA supplementation for pregnant women from 68.7% to 80% by the year 2017

4. Increase compliance to IFA supplementation (for more than 90 days) from 2.5% to 30% by 2017

5. To develop an effective and efficient supply chain management system of IFA commodities

6. To strengthen the coordination of IFA interventions among key stakeholders

7. Improve monitoring and support for IFA supplementation at all levels

2.2 Process of Developing Plan

The process of developing the IFA supplementation plan included:

(1) An initial IFA supplementation meeting among key stakeholders who are members of the supplementation committee under the National Micronutrient Deficiency Control Council (NMDCC). The main purpose of the meeting was to share the challenges currently faced in the IFA intervention and to forge a way forward. From the meeting, consensus was drawn regarding the need to have an elaborate plan to unlock the barriers in the intervention and;

(2) A IFA supplementation stakeholder workshop was held in February 2012 to develop a comprehensive costed plan of action (2012-2017) to be implemented under the leadership of the Division of Nutrition;

(3) the Plan of Action 2012-2017 was reviewed and adopted by the National Micronutrient Deficiency Control Council (NMDCC) and the Nutrition Inter-agency Coordinating Committee (NICC); and

(5) A national dissemination meeting with key stakeholders was held where partners committed resources for the implementation of the 5 year plan.

2.3 The Action Plan

The plan is based on five focus areas outlined below together with the expected outcomes.

TABLE 1: IFA Supplementation Plan: Focus Areas and Expected Outcomes

FOCUS AREAS	ISSUE ADDRESSED	EXPECTED OUTCOME
1. Policies and legislation	Need for heightened advocacy among key policy makers to ensure prioritization of nutrition	National Food Security and Nutrition Policy enacted, Micronutrient Strategy and Technical Guidelines reviewed
2. Capacity development and service delivery strengthening	Health workers knowledge, attitudes and practices on IFA interventions, including supplementation	Improved quality of IFA supplementation service delivery at all levels
3. Advocacy, partnership and communication	BCC needed to address barriers and facilitating factors for increasing IFA uptake, utilization and compliance among pregnant women	Increased awareness and support for IFA interventions
4. IFA commodities and supply chain management	The entire supply chain system including forecasting, quantification, procurement, distribution and storage mechanisms	Effective and efficient supply chain management system
5. Monitoring, evaluation and research	Need to strengthen M&E at all levels from health facility to national level. This includes data reporting tools, sensitization of health workers on importance of appropriate and timely reporting	Quality and timely implementation of evidence-based IFA interventions

The proposed budget for the 6-year IFA supplementation plan is approximately Kes 1 billion (~$12 million). The implementation framework and summary budget for the plan is indicated in Table 2.

TABLE 2: IFA Supplementation Plan: Implementation Framework and Summary Budget for the period 2012-2017

OUTPUTS	ACTIVITIES	INDICATORS	BASELINE	TARGET	LEAD	IMPLEMENTING PARTNERS	UNIT COST	TOTAL BUDGET KES
**Focus Area 1:** Policies and Legislation<br>Expected Outcome: Food and Nutrition Security Policy Enacted and reviewed Micronutrient Strategy and Technical Guidelines								
Increased prioritization of nutrition and funding at national and county levels	Parliamentary caucus meetings/ County Assembly	% of health sector funding of the total national budget<br><br>% of nutrition sector funding of the total health budget	8%<br><br>2%	15%<br><br>5%	MOH - DON	UNICEF, MI, MCHIP	Quarterly Meetings (50 pax)<br>Development & printing of briefing materials<br>(2,000 copies)	7,400,000
	County/Parliamentary health committee meetings				MOH – DON	UNICEF, MI, MCHIP	Quarterly meetings (50 pax)	7,000,000
	2-day nutrition symposium for launching SUN				MOH – DON	UNICEF, SCUK, WFP, MI	Planning & implementation	16,000,000
	PS's/ County meetings				MOH, MOA	UNICEF, FAO, WFP	2 meetings (15 pax)<br>Conference package<br>Per diem	810,000
	Engagement of potential future leaders and query their manifestos				MOH	UNICEF	Media briefing, development & printing of publications<br>Meetings with potential leaders at county level	2,645,000
	Dissemination of the policies (MIYCN & FSNP) and nutrition action plan				MOH - DON	UNICEF, MI, MCHIP	Production<br>Distribution & storage (15% of total)<br>Dissemination meetings	34,790,000
	Advocate for evidence based financial allocation to the health sector through MTEF process				MOH	UNICEF, WHO	Technical support and lobbying	-

OUTPUTS	ACTIVITIES	INDICATORS	BASELINE	TARGET	LEAD	IMPLEMENTING PARTNERS	UNIT COST	TOTAL BUDGET KES
Micronutrient strategy and technical guidelines implemented	Review micronutrient technical guidelines and micronutrient strategy based on new evidence	Updated strategy and technical guidelines on micronutrient deficiency control	Existing strategy and guidelines	Reviewer strategy and guidelines	MOH – DON	DRH, MI, UNICEF, MCHIP	Technical analysis of multiple strategies<br>Review meeting	28,150,000
	Dissemination of the technical guidelines and micronutrient strategy		-	Disseminated in all counties	MOH - DON	DRH, MI, UNICEF, MCHIP	Production<br>Distribution<br>Dissemination meetings at national and county levels	3,900,000
**FOCUS AREA 1: SUB TOTAL**								**89,795,000**
**Focus Area 2: Capacity Development and Service Delivery Strengthening**<br>**Expected Outcome: Improved quality of IFA service delivery at all levels**								
Barriers and facilitating factors to IFA interventions identified and addressed	Bottle neck analysis on IFA supply, demand, KAP and monitoring	Recommendations from the bottle-neck analysis implemented	-	100%	MOH – DON	DRH, HMIS, KEMSA, UNICEF, MI, MCHIP	Formative Assessment (mid and end-term)<br>Supply Chain Analysis (mid and end-term)<br>M&E audit	39,060,000

OUTPUTS	ACTIVITIES	INDICATORS	BASELINE	TARGET	LEAD	IMPLEMENTING PARTNERS	UNIT COST	TOTAL BUDGET KES
Improved knowledge, attitude and skills among health workers at all levels	Advocate for review of pre-service curricula to strengthen IFA	Proportion of health workers who adhere to the IFA protocol	-	80% of health workers	MOH – DON	DRH	Sensitization meetings (1 x 50 pax)	350,000
	Review the in-service training materials to strengthen IFA				MOH - DON	DRH, DCAH, MI, UNICEF, MCHIP	Review workshops<br>Production	20,250,000
	Training of health workers at all levels				MOH - DON	DRH, DCAH, UNICEF, MI, MCHIP	Workshops<br>Logistics support for OJT, CME and mentorship including exchange program	45,120,000
	Develop and disseminate IFA job aids				MOH – DON	DRH, UNICEF, MI, MCHIP	Development workshop<br>Production and distribution	8,550,000
**FOCUS AREA 2: SUB TOTAL**								**113,330,000**
**Focus Area 3: Advocacy, Partnership and Communication**<br>**Expected Outcome: Increased awareness and support for IFA interventions**								
Improved partnership and coordination among key stakeholders on IFA	Develop Advocacy, Communication and Social Mobilization (ACSM) plan on IFA supplementation based on formative assessment	Proportion of the ACSM strategic objectives achieved	-	100%	MOH – DON	DRH, MI, UNICEF, MCHIP	Technical assistance for development of IFA plan<br>Strategy Development Workshops<br>Production<br>Dissemination meetings at national level	3,600,000
	Develop and implement TOR for supplementation sub-committee	Proportion of the IFA TOR of the supplementation committee achieved	-	100%	MOH – DON	DRH, UNICEF, MI, MCHIP	Monthly Subcommittee meetings	150,000
	Advocate inclusion of IFA agenda in all nutrition-related coordination forums	Proportion of nutrition coordination forums supporting IFA interventions	-	100%	MOH – DONS	MOH - DFH, UNICEF, MI, MCHIP		1,500,000

OUTPUTS	ACTIVITIES	INDICATORS	BASELINE	TARGET	LEAD	IMPLEMENTING PARTNERS	UNIT COST	TOTAL BUDGET KES
Improved KAP among health workers and communities on IFA	Review, harmonize and develop IEC materials to include IFA messages	Proportion of health workers who adhere to the IFA protocol[1]<br>Proportion of pregnant women receiving combined IFA supplements<br>Proportion of pregnant women consumption a minimum of 90 tablets	60% for Iron	80% for combined IFA	MOH – DON	DRH, UNICEF, MI, MCHIP	Review workshops<br>Production and Pretesting	8,400,000
	Utilize multiple channels for communication including HWs, media, CHWs and religious institutions				MOH – DON	DRH, UNICEF, MI, MCHIP	Dissemination through all channels<br>• Media<br>• Meetings (e.g. IRCK)	40,000,000
**FOCUS AREA 3: SUB TOTAL**								**53,650,000**
**Focus Area 4:** IFA Commodities and Supply Chain Management<br>Expected Outcome: Effective and efficient supply chain management system								
Improved supply chain management system	Development of IFA F&Q guidelines and tools	Proportion of health facilities with no IFA stock-outs	Existing draft document	Finalized & implemented guidelines and tools	MOH – DRH	DON, KEMSA, MI, UNICEF, MCHIP	Workshops<br>Production<br>Dissemination meetings	10,100,000
	Development of a IFA procurement, storage and distribution plan with all stakeholders		-	Final annual plans	MOH – DRH	DON, KEMSA, MI, UNICEF, MCHIP		-
	Procure and distribute IFA commodities		TBD	100%	MOH - DRH	DON, KEMSA, MI, UNICEF, MCHIP	Combined IFA tablets<br>Distribution and storage	672,000,000
	Capacity building of health workers on IFA F&Q and supply chain management at all levels	Proportion of health workers trained on IFA F&Q and supply chain management	-	80% of health worker trained	MOH - DRH	DON, KEMSA, MI, UNICEF, MCHIP	Workshops<br>Technical and Logistics support for OJT, CME and mentorship	9,870,000

[1] IFA protocol to be further developed

OUTPUTS	ACTIVITIES	INDICATORS	BASELINE	TARGET	LEAD	IMPLEMENTING PARTNERS	UNIT COST	TOTAL BUDGET KES
**FOCUS AREA 4: SUB TOTAL**								**691,970,000**
**Focus Area 5:** Monitoring, Evaluation and Research<br>Expected Outcome: Quality and timely implementation of evidence-based IFA interventions								
Improved information management on IFA interventions	Strengthen routine data collection and management on IFA	Proportion of health facilities reporting on IFA	TBC	100%	MOH - DRH	DON, UNICEF, MI, MCHIP	Sensitization meetings<br>Technical and Logistics support for OJT, CME and mentorship<br>Quarterly support supervision	38,400,000
	Review existing IFA indicators in the HIS and periodic surveys	Revised HIS indicator manual to reflect proposed IFA indicators	Existing indicators and tools		MOH - DON	DRH, UNICEF, MI, MCHIP	Review workshops<br>Production of tools<br>Dissemination meetings	12,000,000
Harmonized M&E plans	Align IFA implementation framework to national nutrition M&E strategy	Proportion of indicators in the IFA Implementation plan reflected in the M&E strategy	TBD	100%	MOH - DON	DRH, UNICEF, MI, MCHIP		-
**FOCUS AREA 5: SUB TOTAL**								**50,400,000**
**TOTAL BUDGET (KES)**								**999,145,000**
**TOTAL BUDGET (USD)**								**12,037,891**

ANNEX 1: DETAILED IMPLEMENTATION BUDGET

IFA IMPLEMENTATION FRAME WORK BUDGET

**FOCUS AREA 1:** POLICIES & LEGISLATION. EXPECTED OUTCOME: FOOD SECURITY AND NUTRITION POLICY ENACTED ; MICRONUTRIENT STRATEGY AND TECHNICAL GUIDELINES REVIEWED														
**OUTPUT**	**BROAD ACTIVITIES**	**SPECIFIC ACTIVITIES**	**PAX**	**PAX COST**	**UNITS**	**UNIT COST**	**AMOUNT**	**Y1**	**Y2**	**Y3**	**Y4**	**Y5**	**TOTAL**	**BUDGET NOTES**
Increased prioritization of nutrition and funding at national and county levels	Paliamentary caucaus meetings	Quartely Meetings (50 pax)	50	7000	4	350,000	1,400,000	1,400,000	1,400,000	1,400,000	1,400,000	1,400,000	7,000,000	
		Development & printing of briefing materials (2000 copies)	2000	200	1	400,000	400,000	100,000	100,000	100,000	100,000	100,000	400,000	2000 fact sheet, briefing folder, pamplet
	parliamentary health committee meetings	Quartely Meeting (50 pax)	50	7000	4	350,000	1,400,000	1,400,000	1,400,000	1,400,000	1,400,000	1,400,000	7,000,000	
	Symposium for launching SUN	Planning &Implementation (2day symposium)				16,000,000	16,000,000	16,000,000					16,000,000	
	PSs meeting	PS meeting (2 meetings)	15	7000	2	105,000	210,000	210,000					210,000	7000 confernce package (2 meetings first year
		PS meeting (Per diem)	15	20000	2	300,000	600,000	600,000					600,000	night out/perdiem - 20000
	Engagement of potential future leaders and query their manifestos	Media briefing, development and printing of publications				1,000,000	1,000,000	1,000,000					1,000,000	lumpsum
		Meetings withpotential leaders	5	7000	47	35,000	1,645,000	1,645,000					1,645,000	at least five in every county (5leaders*47 counties*7000 Conference Package)

OUTPUT	BROAD ACTIVITIES	SPECIFIC ACTIVITIES	PAX	PAX COST	UNITS	UNIT COST	AMOUNT	Y1	Y2	Y3	Y4	Y5	TOTAL	BUDGET NOTES
	Dissemination of Policies (MIYCN, FNSP and nutrition action plan	Production (MIYCN &FSNP)	20000	1000	1	20,000,000	20,000,000	20,000,000					20,000,000	10,000 each at 1000/= per copy * 2 documents
		Production NAP	600	1000		600,000	600,000	600,000					600,000	600 copies of action plan * 1000/=
		Distribution				3,090,000	3,090,000	3,090,000					3,090,000	15% of cost of printing (in country)
		Dissemination (national)	100	2000	3	200,000	600,000	600,000					600,000	national 100 pax * 2000 CP * 3 meetings for 3 documents
		Dissemination (3 meetings in 10 regions County)	50	7000	30	350,000	10,500,000	10,500,000					10,500,000	10 regions * 50 pax * 7000 Conference Package(CP) (includes transport reinbursements)*3meetings
	Advocate for evidence based financial allocation to the health sector thro the MTEF process	technical support and lobbying												part of continous programming
Micronutrient strategy and technical guidelines implemented	Review of Micronutrient technical guidelines and micronutrient strategy based on new evidence	Technical analysis of multiple strategies (engage consultant)	1	30000	30	900,000	900,000	900,000					900,000	30 day consultancy @ 30,000/ day (inclusive of transport)

OUTPUT	BROAD ACTIVITIES	SPECIFIC ACTIVITIES	PAX	PAX COST	UNITS	UNIT COST	AMOUNT	Y1	Y2	Y3	Y4	Y5	TOTAL	BUDGET NOTES
		Review meetings	25	7000	2	175,000	350,000	350,000					350,000	25 pax *7000 CP* 2 meetings
		Production (MNTG)	20000	1000	1	20,000,000	20,000,000	20,000,000					20,000,000	10,000 each at 1000/= per copy * 2 documents
		Distribution (MNTG)				3,000,000	3,000,000	3,000,000					3,000,000	
	Dissemination of the technical guidelines and micronutrient strategy	Dissemination (national)	100	2000	2	200,000	400,000	400,000					400,000	national 100 pax * sh 2000 (cost for morning 8-11.30am (cocktail) * 2 meeting
		Dissemination (County)	50	7000	10	350,000	3,500,000	3,500,000					3,500,000	10 regional meetings * 50 pax * 7000 CP (includes transport reinbursements)* 1meetings (the other meeting is covered in the capacity buiding (orientation on guidelines)
								83,895,000	1,500,000	1,500,000	1,500,000	1,500,000	89,795,000	
FOCUS AREA 2: CAPACITY DEVELOPMENT AND SERVICE DELIVERY STRENGTHENING. EXPECTED OUTCOME: IMPROVED QUALITY OF IFA SERVICE DELIVERY AT ALL LEVELS														
Barriers and facilitating factors to IFA interventions identified and addressed	Bottleneck analyis on IFA supply, demand, KAP and monitoring	Formative assessment (consultancy) (mid and end term)	1	30000	40	30,000	1,200,000	1,200,000		1,200,000		1,200,000	3,600,000	consultancy - 40 days * 30,000

OUTPUT	BROAD ACTIVITIES	SPECIFIC ACTIVITIES	PAX	PAX COST	UNITS	UNIT COST	AMOUNT	Y1	Y2	Y3	Y4	Y5	TOTAL	BUDGET NOTES
		Formative assessment (Logistics for field work)	1	360000	20	360,000	7,200,000	7,200,000		7,200,000		7,200,000	21,600,000	field cost/logistics - 20days * 360,000/= (vehicle, driver,fuel, communication and enumeration)
		Supply chain analysis (mid and end term)				3,360,000	3,360,000	3,360,000		3,360,000		3,360,000	10,080,000	40% of the formative
		M&E audit				1,260,000	1,260,000	1,260,000		1,260,000		1,260,000	3,780,000	15% of the formative
Improved knowledge, attitude and skills among health workers at all levels	Advocate for review of preservice curriculum to strengthen IFA	Sensitization meeting (50 pax)	50	7000	1	350,000	350,000	350,000					350,000	50 pax * 7000 CP
	Review the in-service training materials to strengthen IFA	Review workshops	15	10000	15	150,000	2,250,000		2,250,000				2,250,000	15 pax * 10,000/= * 3 workshops * 5 day workshop)
		Printing	300	1000	60	300,000	18,000,000		9,000,000	3,000,000	3,000,000	3,000,000	18,000,000	3 *2 documents facilitator + participant (community maternal & newborn care guidelines, MIYCN guidelines, Fanc) * 1000/= * 50 copies*60 counties
	Training of HW at all levels	Workshops	30	7000	47	210,000	9,870,000		4,935,000	4,935,000			9,870,000	10 TOT/County *47 counties * 3 days * 7000/=

OUTPUT	BROAD ACTIVITIES	SPECIFIC ACTIVITIES	PAX	PAX COST	UNITS	UNIT COST	AMOUNT	Y1	Y2	Y3	Y4	Y5	TOTAL	BUDGET NOTES
		Logistic support for OJT	2350	3000	1	7,050,000	7,050,000	7,050,000	7,050,000	7,050,000	7,050,000	7,050,000	35,250,000	1 day/2350 visits * 3000/= (fuel) * 1 time every year * 1 HW (1000 lunch, 2000 fuel)
	Develop and diseminate IFA job aids	Development (IFA job aids)	60	20000	1	1,200,000	1,200,000		1,200,000				1,200,000	15pax * 20,000/= * 4 days
		Production (Job aidsS)	14000	500	1	7,000,000	7,000,000		3,500,000	3,500,000			7,000,000	7000 facilities * 2 copies * 500/= (flip chart, protocol)
		Distribution				350,000			175,000	175,000			350,000	5% of printing
							58,740,000	20,420,000	28,110,000	31,680,000	10,050,000	23,070,000	113,330,000	
FOCUS AREA 3: ADVOCACY, PARTNERSHIPS AND COMMUNICATION. EXPECTED OUTCOME: INCREASED AWARENESS AND SUPPORT FOR IFA INTERVENTIONS														
Improved partnerships and coordination among key stakeholders on IFA	Develop ACSM strategy on IFA based on formative assessment	Technical assistance to develop BCC strategy (Consultancy)	1	30000	30	30,000	900,000	900,000					900,000	consultancy 30 days * 30,000/= (350USD)
		Strategy Workshop	75	10000	2	750,000	1,500,000	750,000	750,000				1,500,000	15 pax * 2 workshops * 5 day workshops * 10,000/=
		Production	1000	1000	1	1,000,000	1,000,000		1,000,000				1,000,000	1000 copies * 1000/=
		Dissemination meeting (national)	100	2000	1	200,000	200,000		200,000				200,000	national 100 pax * 2000 CP * 1 documents

OUTPUT	BROAD ACTIVITIES	SPECIFIC ACTIVITIES	PAX	PAX COST	UNITS	UNIT COST	AMOUNT	Y1	Y2	Y3	Y4	Y5	TOTAL	BUDGET NOTES
	Develop and implement TOR for the supplementation sub commitee	Monthly sub committee meetings	25	100	12	2,500	30,000	30,000	30,000	30,000	30,000	30,000	150,000	25 pax * 100/= teas and snacks * 12 months
	Advocate for inclusion of IFA agenda in all nutrition-related coordination forums	Advocacy												to be done as part of ongoing activities
Improved KAP among health workers and communities on IFA	Harmonize IEC materials to include IFA messages	Harmonization workshop	15	10000	10	150,000	1,500,000	1,500,000					1,500,000	15 pax *10000* 2 meetings * 5days
		Production and pretesting of IEC	5600	1500	1	8,400,000	8,400,000	8,400,000					8,400,000	review and reproduction 5600 copies * 1500/=
	Utilize multiple channels for communication including HWs, media, CHWs, and religious institutions	Disseminate through channels (media + meetings)				40,000,000	40,000,000		20,000,000	10,000,000	10,000,000		40,000,000	radio spots and TV spots, bill boards (40M lumpsum) phased approach
							53,530,000	11,580,000	21,980,000	10,030,000	10,030,000	30,000	53,650,000	
**FOCUS AREA 4: IFA COMMODITIES AND SUPPLY CHAIN MANAGEMENT. EXPECTED OUTCOME: EFFECTIVE AND EFFICIENT SUPPLY CHAIN MANAGEMENT SYSTEM**														
Improved supply chain management system	Develop F&Q guidelines and tools	Workshops	15	10000	10	150,000	1,500,000	1,500,000					1,500,000	15 pax *10000* 2 meetings * 5days

OUTPUT	BROAD ACTIVITIES	SPECIFIC ACTIVITIES	PAX	PAX COST	UNITS	UNIT COST	AMOUNT	Y1	Y2	Y3	Y4	Y5	TOTAL	BUDGET NOTES
	Develop IFA procurement, storage and distribution plan with all stakeholders	Production	5600	1500	1	8,400,000	8,400,000	8,400,000					8,400,000	80% of 7000 total health facilities = 5600 copies * 1500/=
		Dissemina-tion meetings (national)	100	2000	1	200,000	200,000		200,000				200,000	national 100 pax * 2000 CP * 1 documents
	Procure-ment of IFA comodities	Combined IFA tabltes	1600000	80	1	128,000,000	128,000,000	128,000,000	128,000,000	128,000,000	128,000,000	128,000,000	640,000,000	1.6M pregnant mothers * 80/= (every year) (use 2.7% increase) 80/= cost at which it gets incountry
		Distribution and storage				6,400,000	6,400,000	6,400,000	6,400,000	6,400,000	6,400,000	6,400,000	32,000,000	5% of procure-ment
	Capacity building of HW on F&Q and supply chain manage-ment at all levels	Workshops	30	7000	47	210,000	9,870,000		4,935,000	4,935,000			9,870,000	10 TOT/County *47 counties * 3 days * 7000/=
														OJT to be combined with above
							154,370,000	144,300,000	139,535,000	139,335,000	134,400,000	134,400,000	691,970,000	
FOCUS AREA 5: MONITORING, EVALUATION AND RESEARCH. EXPECTED OUTCOME: QUALITY AND TIMELY IMPLEMENTATION OF EVIDENCE-BASED IFA INTERVENTIONS														
improved information management on IFA inter-ventions	Strengthen routine data collection and management	Sensitization meetings												part of focus area 2

OUTPUT	BROAD ACTIVITIES	SPECIFIC ACTIVITIES	PAX	PAX COST	UNITS	UNIT COST	AMOUNT	Y1	Y2	Y3	Y4	Y5	TOTAL	BUDGET NOTES
		Technical and logistical support for OJT, CME and mentorship												part of focus area 2
		Quarterly supportive supervision	80	4000	1200	320,000	384,000,000	7,680,000	7,680,000	7,680,000	7,680,000	7,680,000	38,400,000	300 districts * (8 DHMT * 4000/= DSA)+3000 fuel/day) * 10 days * 4 times per year = 420M - our contribution to IFA = 2% of 420M= 8.4M
	Review existing IFA indicators in the HIS & periodic surveys	Fuel	10	3000	1200	30,000	36,000,000	720,000	720,000	720,000	720,000	720,000	3,600,000	
		Production of tools	5600	1500	1	8,400,000	8,400,000	8,400,000					8,400,000	
		Review workshops												part of normal programming/to use existing fora
		production of tools												
Harmonized M&E plans	Align IFA implementation framework to national nutrition M&E strategy													to be done as part of M&E strategy development work
							428,400,000	16,800,000	8,400,000	8,400,000	8,400,000	8,400,000	50,400,000	
**TOTAL BUDGET (KES)**													**999,145,000**	
**TOTAL BUDGET (USD)**													**12,037,891.57**	

ANNEX 2: LIST OF CONTRIBUTORS

NAME	ORGANIZATION
Terry Wefwafwa	DON - MOPHS
Evelyn Kikechi	DON - MOPHS
Valerie Wambani	DON – MOPHS
James Njiru	DON – MOPHS
John Mwai	DON - MOPHS
Sarah Onsase	DON-MOPHS
Samuel Murage	DON - MOPHS
Evelyn Matiri	USAID - MCHIP
Herbert Kere	USAID - MCHIP
Crispin Ndedda	DCAH - MOPHS
Alex Mutua	DCAH - MOPHS
Josephat Mutua	DCAH-MOPHS
Patrick Warutere	HMIS
Charity Tauta	DCHS - MOPHS
Samuel Muchiri Kamau	MI/DON
Cosmas Mutunga	DRH - MOPHS
Esther Kariuki	MI
Ruth Situma	UNICEF
Marjorie Volege	UNICEF
Lucy Maina-Gathigi	DON - MOPHS
Rae Galloway	USAID - MCHIP
Judith Kimiywe	USAID - MCHIP
Kiersten Israel	USAID - MCHIP

REFERENCES

1. Mwaniki, D. L., Omwega, A. M., Muniu, E. M., Mutunga, J. N., Akelola, R., Shako, B., Gotink, M. H. and Pertet, A. M. (1999). Anaemia and Micronutrient Status of Iron, Vitamin A and Zinc in Kenya; National Micronutrient Survey Report.

2. Jane, B., Michael, B. and Klaus, K. (2007). The guidebook. Nutritional Anemia. Sight and Life Press. pp 1-11.

3. Ross, J. and Horton, S. "Economic Consequences of Iron deficiency." The Micronutrient Initiative, 1998: Pp 26.

4. Kenya National Bureau of Statistics (KNBS) and ICF Macro, 2010. Kenya Demographic and Health Survey 2008-09. Calverton, Maryland: KNBS and ICF Macro: 113-118.

5. Central Bureau of Statistics (CBS) [Kenya], Ministry of Health (MOH)[Kenya], and ORC Macro. (2004). Kenya Demographic and Health Survey 2003. Calverton, Maryland: CBS, MOH, and ORC Macro: 163-167.

6. National Coordinating Agency for Population Development (NCAP) [Kenya], Ministry of Medical Services (MOMS) [Kenya], Ministry of Public Health and Sanitation (MOPHS) [Kenya], Kenya National Bereau of Statistics (KNBS) [Kenya], ICF Macro. 2011. Kenya Service Provision Assessment Survey 2010. Nairobi, Kenya.

7. MOPHS. (2008). The Kenya National Technical Guidelines for Micronutrient Deficiency Control. Government of Kenya. pp 49-71.

USAID
FROM THE AMERICAN PEOPLE
MCHIP Maternal and Child Health Integrated Program
Micronutrient Initiative
unicef
