# Supplementary material for: Time for change is now: Experiences of participants in a community-based approach for iron and folic acid supplementation in a rural county in Kenya, a qualitative study
Source: PLoS One. 2020 Jan 16;15(1):e0227332. doi: 10.1371/journal.pone.0227332 (PMC6964883; doi:10.1371/journal.pone.0227332)
Supplement: S4 File — This is one of the training materials used for training the community health volunteers. (PDF) [file pone.0227332.s004.pdf]

MINISTRY OF HEALTH

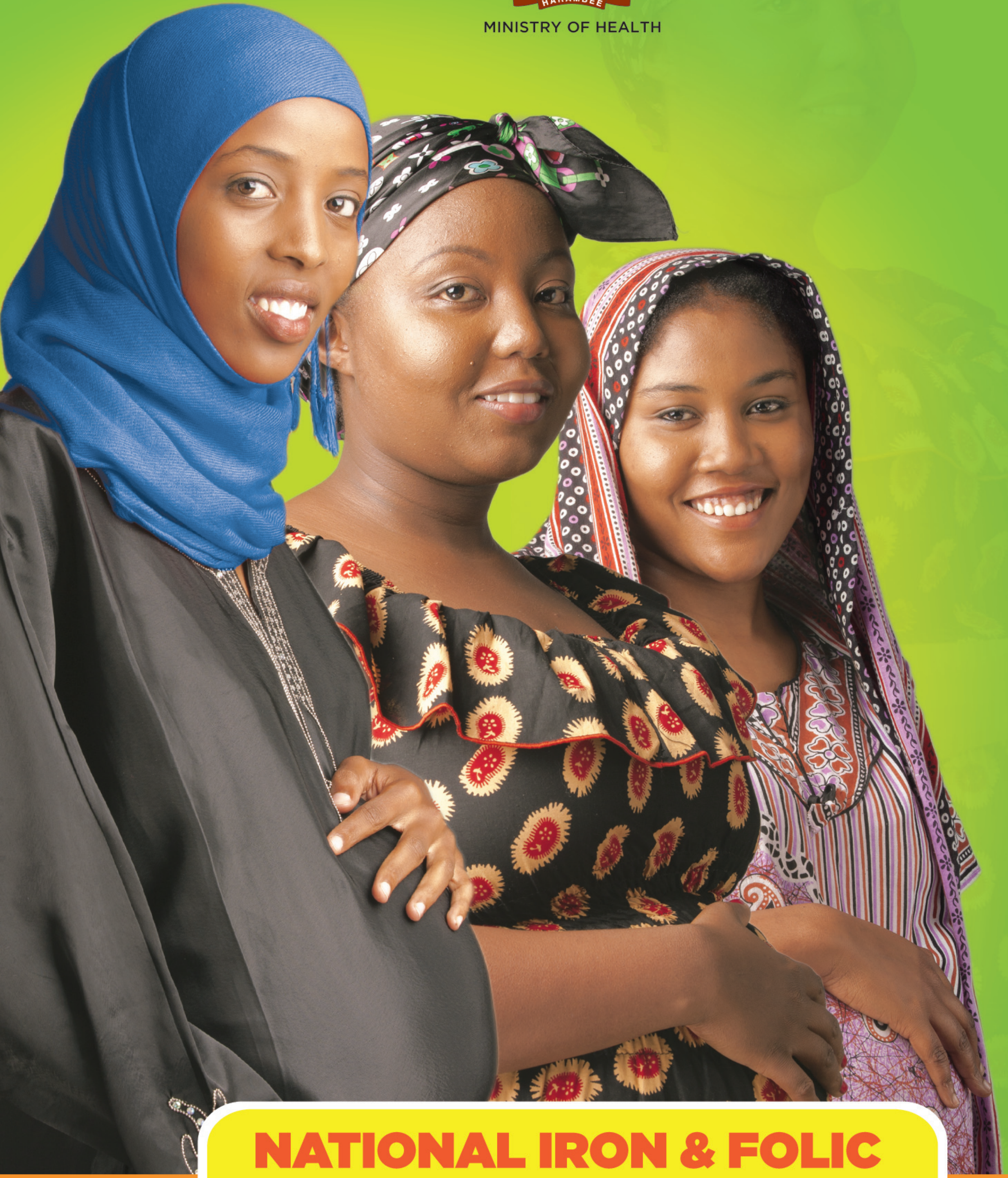

NATIONAL IRON & FOLIC ACID SUPPLEMENTATION

COMMUNICATION STRATEGY

2013 – 2017

NATIONAL IRON & FOLIC ACID SUPPLEMENTATION COMMUNICATION STRATEGY 2013 - 2017

Suggested citation:
Ministry of Health, Republic of Kenya.
National Iron and Folic Acid Supplementation
Communication Strategy Nairobi, Kenya:
Government of Kenya; February 2013
@2013 Government of Kenya

Enquiries and Feedback:
Direct all correspondence to:
Head,
Division of Nutrition
Ministry of Health
P.O. BOX 30016 GPO
Nairobi, 00100, Kenya

TABLE OF CONTENTS

Acronyms and Abbreviations 5
Foreword 6
Acknowledgement 6
Executive Summary 8

CHAPTER 1: INTRODUCTION 9
1.1. Background to Iron and Folic Acid Supplementation 9
1.2. Current IFAS Situation in Kenya 9
1.3. Policy and Legal Framework 10
1.4. The IFAS Communication Strategy Development Process 12

CHAPTER 2: IFAS COMMUNICATION STRATEGY 14
2.1. The Goal and Objectives of the Strategy 14
2.2. Key Strategies for IFAS Interventions 14
2.3. IFAS Problem Analysis 16
2.4. IFAS People Analysis and Audience Segments 19
2.5. IFAS Context Analysis 21
2.6. Behaviour Change Analysis for Priority Target Audiences 24
2.6.1. Change Analysis for Pregnant Mothers 24
2.6.2. Change Analysis for Health Care Providers on IFAS 25
2.6.3. Change Analysis for Community Health Workers 26
2.6.4. Change Analysis for Influencers 27
2.6.5. Change Analysis for Policy Makers 28
2.7 IFAS Key Message Themes 29
2.8. Communication Channels and Material Ideas 32
2.9. Best Practices on Ifas Utilization and Coverage 34

Case Study: Pandey-Nepal 34
Case Study: Thailand 34
Case Study: Indonesia 35

CHAPTER 3: IFAS IMPLEMENTATION PLAN 36
IFAS Implementation Plan 37

CHAPTER 4: SUMMARY BUDGET 41

ACRONYMS AND ABBREVIATIONS

**ANC**	Antenatal Care
**BCC**	Behaviour Change Communication
**CHS**	Community Health Strategy
**CHW**	Community Health Worker
**DCH**	Department of Child Health
**DCHS**	Department of Community Health Services
**DH**	District Hospital
**DHIS**	District Health Information Software
**DON**	Division of Nutrition
**FGD**	Focus Group Discussion
**HF**	Health Facility
**HIV**	Human Immunodeficiency Virus
**IEC**	Information, Education and Communication
**IFA**	Iron and Folic Acid
**IFAS**	Iron and Folic Acid Supplementation
**LBW**	Low Birth Weight
**KAP**	Knowledge, Attitudes and Practices
**KDHS**	Kenya Demographic and Health Survey
**KSPA**	Kenya Services Provision Assessment
**M&E**	Monitoring and Evaluation
**MCH**	Mother and Child health
**MDG**	Millennium Development Goals
**MI**	Micronutrient Initiative
**MOH**	Ministry of Health
**MOMS**	Ministry of Medical Services
**MOPHS**	Ministry of Public Health and Sanitation
**NGO**	Non-Governmental Organization
**NMS**	National Micronutrient Survey
**UNICEF**	United Nations International Children's Emergency Fund
**WHO**	World Health Organization

FOREWORD

The National Iron and Folic Acid Supplementation programme is guided by different Policy and National action frameworks. These policy documents include the Kenya Food and Nutrition Security Policy (2011) and The Kenya National Nutrition Action Plan (2011-2017). These documents provide a platform for National and County response to addressing Iron and Folic Acid deficiencies through, among other interventions, supplementation programmes. The Kenya National Health Policy (2012-2030) and the Kenya Health Sector Strategic Plan 2012-2017 provide clear policy objectives and strategies that are supportive of nutrition. The Constitution of Kenya guarantees that every person has the right to health, which includes healthcare services. The Government of Kenya developed the Vision 2030, as its new long-term development plan for the country. To improve the overall livelihoods of Kenyans, the country aims to provide an efficient integrated and high quality affordable health care system. Under the nutrition sector, the Health Strategy aims to strengthen collaboration in order to ensure adequate nutrition for the whole population, through avoiding and managing over, or under nutrition and micronutrient deficiencies. Iron and Folic Acid Supplementation was made a flagship project under the MTP 11 under Vision 2030

According to World Health Organization it is estimated that 41.8% of pregnant women worldwide are anaemic. In Kenya the most recent micronutrient surveyin the country indicated the prevalence of anaemia among pregnant women to be 55.1%, and 46.4% among non-pregnant women. Anaemia is the leading indirect cause of high maternal and neonatal deaths. Iron and Folic Acid Supplementation (IFAS) for pregnant is one of the interventions that has been recommended by WHO and implemented by the Ministry of Health to reduce anaemia levels. IFAS has been implemented through Focused Antenatal Care (FANC) and although this is the case, there have been challenges which have resulted in sub-optimal IFAS coverage rates and very low adherence rates.

This strategy provides a road map that is aimed at improving ANC attendance and IFAS coverage and utilization rates among pregnant women in Kenya in alignment with National IFAS plan targets. We call upon all partners and stakeholders to collaborate and ensure good coordination in the implementation of IFAS interventions to improve the chances ofmaternal and child survival.

Dr.Annah Wamae OGW
Head, Department of Family Health
Ministry of Health

ACKNOWLEDGEMENT

The Division of Nutrition appreciates the efforts by different departments and divisions within the Ministry of Health as well as various partners for their input in the development of this strategy. The consultative and collaborative effort is highly appreciated.

The Division would like to acknowledge the contributions and commitments of various institutions, organizations, partners and individuals who worked diligently towards the successful completion of this document. Special appreciation goes to Evelyn Kikechi (DON), James Njiru (DON) and Esther Kariuki (Micronutrient Initiative) for their technical input.

Gratitude goes to the Department of Child Health, Division of Community Services, and Division of Reproductive Health, Department of Health Promotion and Provincial Nutrition Officers. Appreciation goes to the following organizations that provided technical support; PSI, UNICEF, World Vision, USAID MCHIP, EXP and PATH

Special gratitude goes to Micronutrient Initiative for the generous contribution towards the development of this strategy.

Finally we wish to express our gratitude to the Centre for Behaviour Change and Communication (CBCC) under Essence International, led by Dr. Catherine Lengewa and Ms. June Ndungu for technical assistance and compiling the document.

Terry Wefwafwa HSC
Head, Division of Nutrition
Ministry of Health.

EXECUTIVE SUMMARY

Iron and folic acid deficiency is the most common form of malnutrition in the world affecting more than 2 billion globally. According to World Health Organization it is estimated that 41.8% of pregnant women worldwide are anaemic. In Kenya the most recent micronutrient survey in the country indicated the prevalence of anaemia among pregnant women to be 55.1% and 46.4% among non-pregnant women. Anaemia is the leading indirect cause of high maternal and neonatal deaths. IFA supplementation for pregnant women is one of the interventions that has been recommended by WHO and implemented by the Ministry of Health to reduce anaemia levels. IFA supplementation in Kenya has been implemented through Focussed Antenatal Care (FANC) and although this is the case, there have been challenges which have resulted in sub-optimal coverage rates and very low adherence rates.

The challenges that are attributed to the sub-optimal coverage rates and very low adherence rates include; few ANC visits and inconsistency in ANC care resulting in missed opportunities for IFAS, physical barriers of accessing ANC services, lack of consistency in implementation of IFAS, low adherence and compliance, lack of knowledge on effects of anaemia, poor monitoring of IFAS distribution, interrupted stock outs and lack of supplies, as well as challenges with human resources and the current service delivery model.In order to address some of these challenges successfully, sound behaviour change and communication strategies must be implemented to improve IFAS coverage and utilization among other issues in order to achieve the National IFAS targets of 80% coverage and 30% utilization of 90+ supplements.

The purpose of this strategy is to improve ANC attendance and IFAS coverage and utilization rates among pregnant women in Kenya. It is aimed at guiding the country on key strategies to implement in improving knowledge, attitudes and practices among various target populations on IFAS in alignment with the National IFAS plan targets.

This communication strategy has identified five broad strategies for IFAS interventions to be implemented in the next five years; these include;

- **Strategy one:** Enhance increased resources, political and social commitment for IFAS through advocacy
- **Strategy two:** Empower pregnant mothers to adopt appropriate health practices and health seeking behaviour regarding ANC and IFAS through behaviour change
- **Strategy three:** Cultivate environments that support adoption of positive ANC and IFAS practices through social mobilization
- **Strategy four:** Capacity strengthening for health providers on IFAS to increase utilization rates.
- **Strategy five:** Empower community health workers and volunteers on IFAS to increase coverage and utilization rates.

There is need for concerted effort, coordination and collaboration among partners and stakeholders in the implementation of these strategies in order to realize reduction in the maternal and neonatal mortalities.

CHAPTER 1: INTRODUCTION

1.1. BACKGROUND TO IRON AND FOLIC ACID SUPPLEMENTATION

Iron deficiency is the most common form of malnutrition in the world, affecting more than 2 billion people globally. Iron is important for cognitive development and inadequate iron intake is the main cause of anaemia – a condition which presents as low haemoglobin concentration levels in the blood. Clinical signs of anaemia include pallor of the skin and inner eyelids. In addition to increasing the risk of maternal and infant mortality, iron deficiency impairs learning, decreases physical work capacity, and increases the risk of premature delivery and low birth weight.

Iron deficiency is not the only cause of anaemia, but where anaemia is prevalent; iron deficiency is usually the most common cause. Iron deficiency anaemia is most prevalent and severe in young children and women of reproductive age. According to the World Health Organization (WHO), it is estimated that 41.8% of pregnant women worldwide are anaemic. At least half of this anaemia burden is assumed to be due to iron deficiency with the rest due to conditions such as folate, vitamin B12 or vitamin A deficiency, chronic inflammation, parasitic infections and inherited disorders.

Anaemia is a leading indirect cause of high maternal and neonatal deaths. According to the KDHS 2008-09, maternal deaths increased from 414/100,000 to 488/100,000. The Neonatal deaths decreased marginally from 33/1000 to 31/1000 live births. The Ministry of Health intends to decrease both maternal mortality and neonatal deaths to 147/100,000 and 11/1000 respectively by 20151[20].

Iron and Folic Acid (IFA) supplementation for pregnant women is one of the interventions that has been recommended by WHO and implemented by the MOPHS to reduce anaemia levels. According to WHO, daily iron and folic acid supplementation is recommended as part of the ante natal care to reduce the risk of low birth weight, maternal anaemia and iron deficiency. WHO recommends a daily protocol of 60mg iron supplementation for treatment and prevention in pregnant women and 400Qg of folic acid supplementation before conception and within 28 days after conception. Folic acid significantly reduces the incidence of neural tube defects when taken before conception and within 28 days after conception.

1.2. CURRENT IFAS SITUATION IN KENYA

The Ministry of Health (MOH) is working with partners to strengthen iron and folic acid (IFA) supplementation of pregnant women within the antenatal care services (ANC) in levels 2 and 3 health facilities. The Division of Nutrition (DON) within MOH recently drafted a multi-year plan titled Accelerating Reduction of Maternal Anemia through IFA supplementation of Pregnant Women. Through this plan, the Ministry aims to increase IFA coverage from 68.7% to 80% and to increase IFA utilization from 2.5% to 30% (>90days) by end of 2017.

In Kenya, the most current micronutrient survey in the country indicated the prevalence of anaemia among pregnant women to be high at 55.1% and 46.4% among non-pregnant women and 70% among pregnant women2[21] .The Coast Province, Lake Basin and semi-arid low lands have the highest prevalence.

20 Kenya Demographic Health Survey 2008-2009
2 National Micronutrient Survey (MOH 1999)

Iron and Folic Acid supplementation for pregnant women is one of the interventions that have been implemented by the Ministry of Health to reduce these anaemia levels. This intervention is one of the services provided within the Focused Antenatal Care (FANC) package and is one of the high impact nutrition interventions that are currently being scaled under the global 'Scaling up Nutrition' Movement, of which Kenya is a member.

IFA supplementation has mainly been implemented through FANC. Though IFA supplementation has been implemented for years, it has faced many challenges which have resulted in sub-optimal coverage rates (68.7%2) and very low adherence rates. The last KDHS (2008-09) showed that 54% of women reported taking iron tablets or syrup for less than 60 days during the pregnancy of their most recent birth while only 2.5% took iron supplements for more than 90 days. These figures are very low compared to the recommendation in the new WHO guidelines which indicates daily intake of IFAS throughout pregnancy

he challenges that are attributed to the sub-optimal coverage rates and very low adherence rates include; few ANC visits and inconsistency in ANC care resulting in missed opportunities for IFAS, physical barriers of accessing ANC services, lack of consistency in implementation of IFAS, low adherence and compliance, lack of knowledge on effects of anaemia, poor monitoring of IFAS distribution, interrupted stock outs and lack of supplies, as well as challenges with human resources and current service delivery model. (KDHS 2009-09, KSPA 2010)

1.3. POLICY AND LEGAL FRAMEWORK

The policy documents that have informed the development of this Communication Strategy are the Vision 2030 (Government of the Republic Kenya, 2007), the Kenya National Health Policy 2012 -2030, the Kenya Health Sector Strategic Plan 2012 to 2017 (MoPHS, December 2008), the Kenya Government Constitution, the Millennium Development Goals, Community Health Strategy among others. The key nutrition documents that have guidelines and strategies on IFAS include; the Multi-Year Plan for IFAS, Kenya National Technical Guidelines for Micronutrient Deficiency Control, Food Security and National Nutrition Policy 2012-2017, National Nutrition Action Plan (2012-2017) and draft National Micronutrient Strategy (2012-2017)

The Millennium Development Goals (MDGS) are designed to assist the country to make progress to middle-income status through development plans.It is expected to have met its Millennium Development Goals (MDGs) whose deadline is 2015. The MDGs are eight internationally-agreed goals for socio-economic development that emphasise the following: elimination of extreme poverty and hunger; universal primary education; gender equality; reduction in child mortality; improvement in maternal health; lower HIV/AIDS and major disease incidence; environmental sustainability; and better partnerships with international development partners (Vision 2030).

Greater emphasis is on the activities that are expected to result in achievement of MDGs 1,2,3,4,5, and 6 that have a direct impact on the health of the child and women of reproductive age. It important to note that the Medium Term Plans (MTP) mainstreams the nutrition budget within the national development planning process that ensures allocation of domestic resources to nutrition programmes. Budget lines for nutrition have also been established for several ministries as outlined in the **Food and Nutrition Security Policy**[22].

The Kenya Constitution 2010[23] states thatevery person has the right to life and no person shall be arbitrarily deprived of life (Chapter 4, article 26). Further, the constitution states that; every person has the right to health, which includes the right to health care services, including reproductive health care. It proposes an economic and social bill of rights which aims to ensure availability of adequate food of acceptable quality and clean and safe water in adequate quantity for all citizens.

The Vision 2030 has been developed by the country as the new long-term development plan. The country aims to provide an efficient integrated and high quality affordable health care system. The Health Strategy under the nutrition sector aims to strengthen collaboration in order to ensure adequate nutrition for the whole population, through avoiding and managing over, or under nutrition and micronutrient deficiencies. IFAS was made a flagship project under the Medium Term Plans (MTP) 11 under Vision 2030.

The Kenya Health Strategic Plan 2012-2017[24] outlines the health sector strategies aimed at achieving the national priorities. Itaims at implementing the Kenya Essential Package of Health (KEPH) to support in the collaboration with health related sectors. Under nutrition sector the package includes the following set of interventions: nutrition education and counselling, community based growth monitoring, micronutrient supplementation, management of acute malnutrition, health education on appropriate infant and young child feeding and promotion of safe food handling which are also part of HINI and IFA implementation. The strategy outlines the five different cohorts which represent the stages in the life cycle of the people. The target population for VAS, IFAS, and Zinc/ORS fall within the first two cohorts. Cohort one is the pregnancy and new-born (up to 28 days), cohort two is the childhood (29 months to 59 months).

Food and Nutrition Security Strategy (FNSS)[25] .The FNSS highlights the priority interventions that are important for improving nutritional status of women of reproductive age under five children, reducing micronutrient deficiencies, and increasing awareness on food fortification. The goal of fortification is to reduce micronutrient deficiencies which include Vitamin A deficiency and Iron deficiency.

The National Nutrition Action Plan 2012-17[26] provides a framework for coordinated implementation of nutrition intervention activities by the Government and stakeholders. It highlights efforts to achieve the High Impact Nutrition Interventions (HINI) which include; exclusive breastfeeding, timely complementary feeding, iron folate, vitamin A and zinc supplementation, hand washing, deworming, food fortification and management of moderate and severe malnutrition. The strategic objective is to ensure that the population receives adequate amounts of micronutrients through dietary diversification, supplementation and fortification at all levels.

---

22 Republic of Kenya: Draft Food and Security and Nutrition Policy, 2007
23 Constitution of Kenya (GoK 2010)
24 Kenya Health strategic plan 2012-2017 (MOPHS 2012)
25 Republic Of Kenya: Draft Food Security and Nutrition Strategy 2007
26 (MoPHS, National Nutrition Action Plan: 2012-2017 2012)

One of the key priority areas is strengthening the national micronutrient supplementation program. The outcome indicator is the percentage reduction of iron deficiency among women of reproductive age.

National Policy guideline on combined Iron and Folic Acid Supplementation for pregnant mothers in Kenya 2013: this is important for guiding implementation of IFAS in the country in terms of dose, frequency, duration, and target group, types of supplements, administration and side effects management.The new guidelines were recently drafted and are in the process of dissemination.

The Kenya National Technical Guidelines for Micronutrient Deficiency Control recommend IFA supplementation for all pregnant women. Operational policies and guidelines as well as one-page fact sheets on IFA supplementation are in place to guide service delivery at facility levels.

1.4. THE IFAS COMMUNICATION STRATEGY DEVELOPMENT PROCESS

This strategy was developed through a consultative and collaborative process of various stakeholders. A comprehensive desk review was conducted on all the issues related to IFAS. A formative assessment was also conducted to understand the underlying issues affecting coverage and utilization of selected micronutrient interventions(Iron and Folic Acid, Vitamin A, Zinc) in selected districts. The findings of this assessment have informed the various components of this strategy. Similarly a consultative process with key partners and stakeholders was conducted through a workshop to develop the strategy and messages jointly based on the key findings from desk review and formative assessment.The development of this strategy has been aligned to the socio-ecological model of analysis (Figure 1).

Figure 1: Social Ecological Model for Change

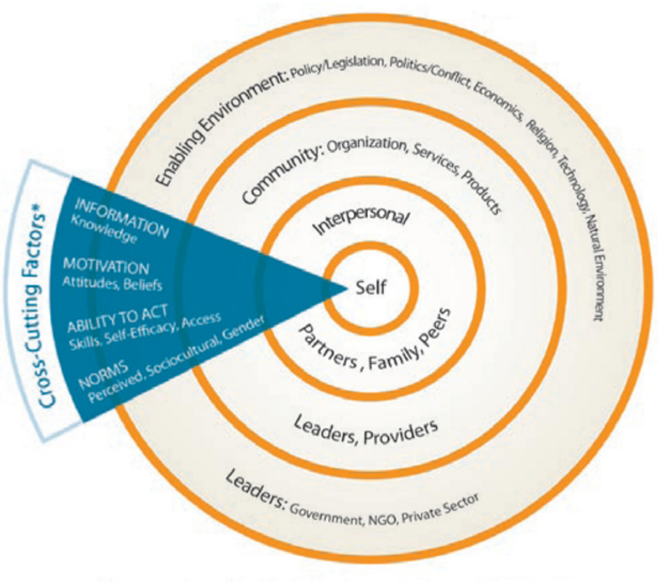

These concepts apply to all levels (people, organizations and institutions).
They were originally developed for the individual level.
SOURCE: Adapted from McKee, Manoncourt, Chin and Carnegie (2000)

The socio-ecological model is based on existing theories, models and approaches from several disciplines including sociology, psychology, communication and political science. It combines the ecological models and psychosocial factors that help in the analysis and planning of various sections in this strategy. The model incorporates newer approaches to social and behaviour change where focus is not only on individual change but on creating or reinforcing social conditions and social relationships that facilitate and encourage healthy behaviour.It proposes several levels of influence to find effective ‘tipping points’ for change. This strategy has been developed using a systematic approach that ensures the application of different levels of analysis to help build the IFAS program around evidence and models that have worked. Different steps have been used to analyse the domains of influence as well as people representing them at each level.

- **Step one: IFAS Problem analysis:** This level of analysis provides insights into the IFAS situation, the causes and broader view of possible effects. As a result it helps in addressing the problem and situation more effectively.

- **Step two: IFAS People analysis:** This level of analysis referred to as the people analysis, helps to clearly highlight the people who are either directly affected by IFAS problem or involved with them in some way. The process brings out the primary audience, secondary audiences, and tertiary audiences.

- **Step three: IFAS Context analysis:** This section of the strategy identifies the cross cutting factors which the IFAS social and behaviour change interventions may be able to modify to generate change. These cross cutting factors in the different individual’s context include;
    - Information that is timely, accessible, and relevant on IFAS
    - Motivation which is often represented by attitudes and beliefs about IFAS
    - Skills, Efficacy and Access which determine the different individual’s ability to act
    - Perceived Norms surrounding IFAS which is expressed in socio-cultural and gender issues.

- **Step four: IFAS Behaviour Change analysis:** This section addresses behavioural and contextual barriers that women of reproductive age, in particular the pregnant mothers face regarding IFAS uptake and utilization. The communication objectives are tailored to address the barriers identified. It highlights the IFAS desired changes by the different target audiences based on evidence from studies done in Kenya.

- **Step five: IFAS Message themes:** The messages are based on analysis, strategic design and understanding of the problem, people, context and barriers discussed in preceding sections. The message themes are derived from the communication objectives developed for each target audience.

- **Step six: Key strategies for IFAS Interventions:** This segment provides strategic Social and Behaviour Change Communication approaches which are mutually reinforcing designed to address the IFAS issues and target audience affected by the problem.

- **Step seven: Communication Channels and materials:** This part proposes a combination of channels which will strategically be used to provide greatest impact to the IFAS interventions to improve coverage and utilization.

CHAPTER 2: IFAS COMMUNICATION STRATEGY

2.1. THE GOAL AND OBJECTIVES OF THE STRATEGY

This strategy is aimed at improving ANC attendance and IFA coverage and utilization rates among pregnant women in Kenya. It is aimed at guiding the country on the key strategies to implement to improve knowledge, attitudes and practices of various target populations on IFAS in alignment with National IFAS plan targets.

2.2. KEY STRATEGIES FOR IFAS INTERVENTIONS

This segment highlights the broad strategies that have been identified to address the issues of IFAS coverage and utilization. These strategies are Advocacy at all levels, Behaviour Change Communication using multimedia and participatory approaches, Social Mobilization for partnerships and alliances, Capacity strengthening for health care providers and engagement of Community Health Workers for community level change. These strategies are mutually reinforcing and they address change at different levels. They are interactive and researched processes aimed at changing social conditions and individual behaviours. Availability and access to services and IFAS supplies are critical for the expected change process

A. Strategy One: Advocate for Increased Resources for IFAS at the National and County Level

1) Advocacy to policy and decision makers at the National and County levels to increase resources and build institutional commitment for IFAS
2) Media engagement at National and County level to increase quantity and quality of reporting on IFAS supplementation
3) Advocacy for increased outreach for MCH services particularly for the hard to reach and marginalised communities.
4) Advocacy for improved IFAS logistics and supplies to avoid interruption of IFAS supplies due to stock outs

B. Strategy Two: Empower Pregnant Mothers to Adopt Appropriate Health Practices and Health Seeking Behaviour regarding ANC and IFAS through Behaviour Change Communication Interventions

1) Mass media campaigns: Conduct a robust IFAS mass media campaign through both print, electronic and social media to increase demand for ANC and IFAS services and facilitate social and behaviour change.
2) Interpersonal communication: Conduct one-on-one interactive communication interventions to increase dialogue on IFA supplementation issues and motivate changes in knowledge, attitudes and practices.
3) Community based media: Community dialogue and community media to motivate collective solutions, provide social support on IFAS issues and feedback to broader community

C. Strategy Three: Create an Environment that Supports Adoption of Positive ANC and IFAS Practices through Social Mobilization

1) Mobilize and coordinate ANC and IFAS partners and stakeholders at National and County levels to create wider participation, coalition and ownership of IFAS interventions
2) Conduct community mobilization activities through existing structures at the County and Sub-county level to create an environment that supports appropriate social and behaviour change actions that lead to sustainable change on IFAS.

D. Strategy Four: Capacity Strengthening for Health Providers on IFA Supplementation to Increase Utilization and Adherence Rates

a) Orientation for health workers on IFAS policy and the rationale for prescribing IFAS to all pregnant mothers, key steps for optimal IFAS service delivery, as well as providing summarised IFAS policy

b) Training to improved Interpersonal communication at the start of IFA supplementation: to enhance communication and service delivery strategies that will retain and increase the ANC visits and demand for IFAS by pregnant women following their first visit

c) Training, supportive supervision and provision of tools to improve the system for monitoring IFAS commodities in terms of procurement and distribution, as well as monitoring IFAS coverage

E. Strategy Five: Empower Community Health Workers on IFA Supplementation to Increase Coverage and Utilization Rates

1) Training CHWs and CHEWs on ANC and IFAs: Pregnant women can receive IFAS messages through community health workers and community leaders to motivate them to seek and demand IFAS from both private and public health facilities.

Figure 2: IFAS BCC Strategies

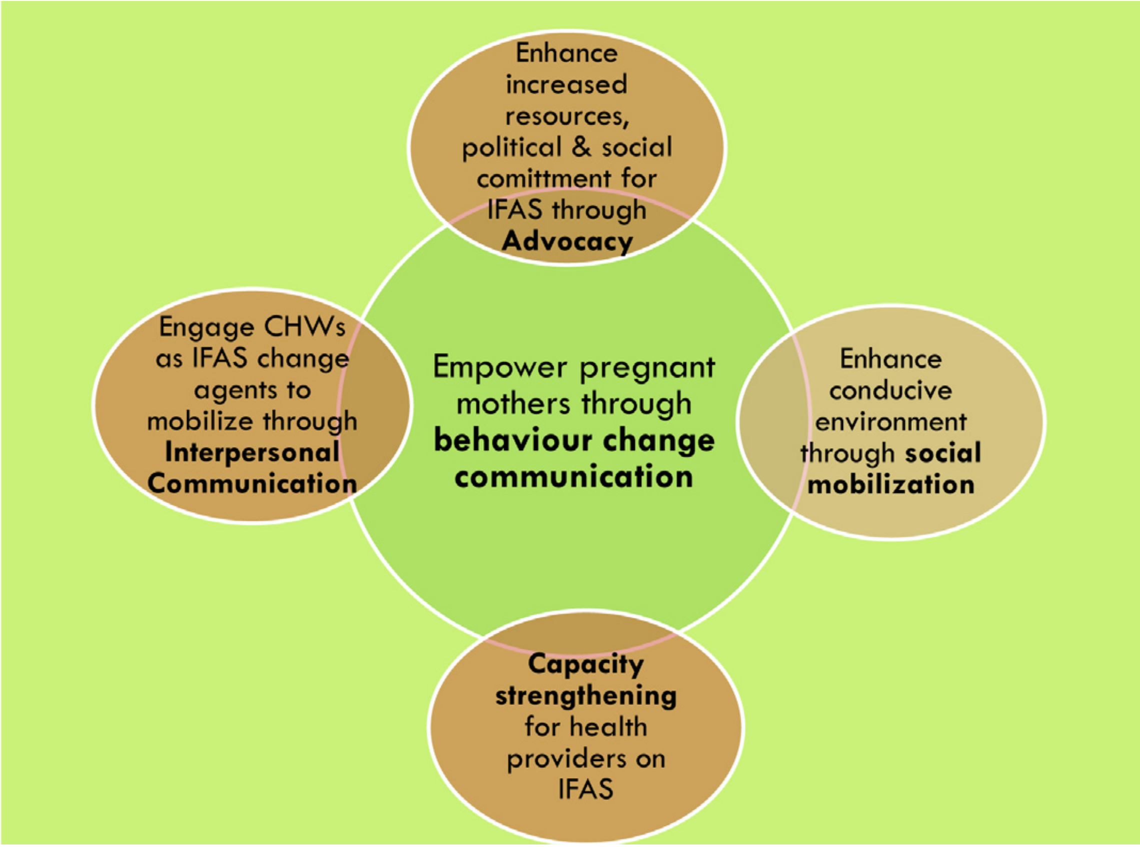

2.3. IFAS PROBLEM ANALYSIS

This section provides some insight into the core issues regarding IFAS that the strategy will purpose to address. The core IFAS problem is the high anaemia rates among women leading to indirect causes of the high maternal mortality rates in Kenya.

The strategy has identified the following key issues from the desk review and research that are associated with IFAS low coverage and utilization rates.

Few ANC visits and sub-optimal ANC practices: Despite the fact that 92% of women in Kenya receive ANC from a medical professional, the timing and the number of visits are still not consistent with the WHO recommendations that indicate a woman without complications should have at least four ANC visits, the first of which should take place during the first trimester. In Kenya, Less than half (47%) of pregnant women make four or more ANC visits and only 15% obtain ANC in the 1st trimester of pregnancy while about half 52% receive care before the 6th month of pregnancy. The median number of months of pregnancy at first visit is above the first trimester at 5.6 months[20] (KDHS 2008-09). One of the main reasons for delayed ANC visits is perceived good health and fear of HIV testing. Other reasons include lack of time and money, long distances to the clinic and lack of knowledge on when to start the clinic among other reasons (MOH 2013)[21] .

Level of education: Examination of differentials in antenatal care in Kenya shows that women's level of education is also associated with antenatal care coverage. Women with higher education are much more likely to receive antenatal care from a medical doctor than those with no education 36 per cent versus 21 per cent (KNSB, ICF 1999)[22].

Inconsistency in ANC results in missed opportunities for IFAS: These challenges are higher among marginalized groups such as poor women, adolescent women, pastoralists and other marginalized groups. In Kenya, two-thirds of women who visit the ANC according to the KDHS 2008-09, received IFA supplementation. Evidently, the uptake of ANC was low and/or sub-optimal among the pastoralists communities in North eastern province (27%), among women without education (49%), the adolescences <20 years of age (64%), and women from households in the lowest wealth quintiles (60.8%)(KNSB/ICF 1999)[23].

Physical barrier to accessing ANC services: IFA is provided to pregnant mothers in health facilities that offer ANC services and through the outreach MCH clinics in the hard to reach areas. The 2010 KSPA[24] has shown that 74 per cent of all facilities offer ANC. For those pregnant women that live around health facilities that do not offer ANC and also those that live far from health facilities offering ANC with no regular outreach clinics, physical barrier to accessing IFASis a major issue. Further, examination of differentials in antenatal care in Kenya shows that rural women are less likely than their urban counterparts to get antenatal care from a doctor, and they are more likely to get no care at all mainly because of the physical barrier. The 2013 MOH has shown over 35% of WRA from Mutomo district (rural) reside over 5km from the nearest antennal clinic compared to 15% from Kitui Central (urban).

20 Kenya Demographic and Health Survey 2008-2009 KNBS and ICF Macro
21 Formative Assessment of Coverage, Uptake, Utilization of Selected High Impact Nutrition Interventions(MOH 2013)
22 (Kenya National Bureau of statistics (KNSB), ICF; 1999)
23 (Kenya National Bureau of statistics (KNSB), ICF; 1999)
24 (Kenya Service Provision Assessment Survey (MoMS 2010)

Lack of consistency in implementation of IFAS: The current country policy on IFA supplementation requires that all pregnant mothers are given IFA supplements whether they have been screened for anaemia or not. However, current practice at the facility level is that health workers insist on screening pregnant women for anaemia before prescribing IFA tablets despite statistics from the 2010 Kenya Service Provision Assessment (KSPA) showing that only a little over 36% of ANC facilities have the capacity to test for anaemia thereby denying IFA supplements to the 'eligible' target group (KSPA 2010)[20] .According to FA MOH 2013, there is a gap in policy dissemination which is one of the barriers to IFAS uptake.

Low adherence and compliance: 2.5% women take >90 supplements. 30% do not take at all (KDHS 200/9). According to IFAS records in the mother child booklet, less than 50% of the mothers received IFAS for up to 60 days, and only 10% received for over 90 days. The common reasons for incomplete intake of IFAS include: limited understanding of IFAS and side effects, Adverse Drug Effects reportedly contributed to discontinuation of IFAS intake or completion of dose. Other reasons include lack of knowledge on duration and dosage of IFAS intake and particularly when to initiate IFAS. Poor management of possible IFAS side effects contributes to challenges of compliance and adherence. Inadequate provider-client interaction contributing to low demand and adherence for IFAS: Only 10% of the mothers were counselled by health providers on how long to take IFAS during pregnancy, only 1% was given reasons for taking the tablets and how to manage side effects (FA MOH 2013)[21] .

Low knowledge on effects and severity of anaemia: According to FA MOH 2013, only 60% of all respondents surveyed were aware of the importance of iron supplements in prevention of anemia during pregnancy. Despite health workers being recognized as the primary source of information for IFAS, awareness on folic acid, IFAS fortified foods and the consequences of anemia in pregnancy if left untreated, were generally low.

Monitoring of IFA distribution: There is lack of a proper system to monitor distribution of supplements. The health information system does not monitor key indicators for iron and folate supplementation (KSPA 2010)[22]. According to FA MOH 2013, monitoring of IFAS intake is a challenge due to poor documentation both in the mother child health booklet and at the facility level.

Logistics and supplies: The IFA supplementation is often interrupted due to frequent stock-outs and lack of supply. According to KSPA 2010, 40% of health facilities have ferrous sulphate while 74% have folate. Perceived causes of frequent IFA stock-outs include: communication breakdown between facilities and depots, low prioritization of IFA supplements by government, differences in forecasting by different MOH units, and inadequate budget allocation. The provision of iron and folic acid as separate tablets negatively affect the utilization of IFA supplements. With the inclusion of combined iron and folic acid supplements in the KEML, Kenya Medical Supplies Agency (KEMSA) has been procuring these supplements. These new tablets are expected to improve compliancesince women ingest fewer tablets compared to the previous separate iron and folic acid tablets.

---

20 Kenya Service Provision Assessment Survey (MOMS 2010)
21 Formative Assessment of Coverage, Uptake, Utilization of Selected High Impact Nutrition Interventions(MOH 2013)
22 Kenya Service Provision Assessment Survey (MoMS 2010)

Service Delivery Models: In accordance with the national policy, IFA supplementation for pregnant women is routinely delivered in all public health facilities through Maternal and Child Health (MCH) clinics as part of the Antenatal Care [FANC]. This strategy is appropriate for long term sustainability.Demand and uptake of IFA is however low despite general awareness of availability of IFA supplements at no cost in public health facilities. Routine service delivery of IFA is hampered by: health worker shortage, limited provider knowledge, periodic stock-outs of IFA and competing priorities. Inadequate engagement of CHWs in IFAS interventions may be associated to poor access to services especially in the hard to reach areas.

Human Resources: Different cadres of health workers have received pre-service and in-service training and are engaged in IFA supplementation through multiple service delivery points. Low knowledge and awareness of IFAS and inadequate counseling materials is hampering optimal delivery of services.The current policy only allows professional health workers to prescribe and dispense IFA supplements to pregnant women but not community health workers (CHWs).
In conclusion, this strategy is designed to address some of the challenges identified through various communication and behaviour change interventions.

Figure 3: IFAS taken daily during pregnancy from conception to delivery

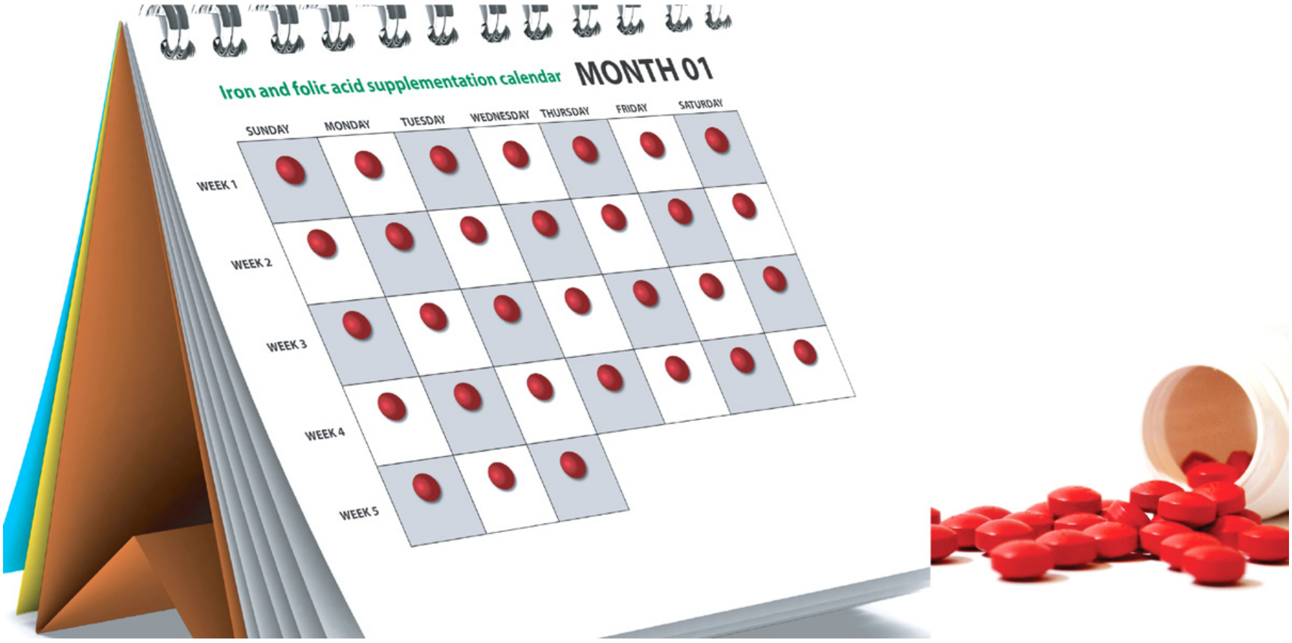

2.4. IFAS PEOPLE ANALYSIS AND AUDIENCE SEGMENTS

This section defines the people most affected (primary audience) by maternal mortality due to anaemia in Kenya. It also defines those directly influencing them (secondary audience) either positively or negatively. Finally, it highlights those who indirectly influence the primary audience by shaping social norms, influencing policy, or offering financial and logistical support. A further analysis has been conducted to segment the primary audience to provide a clearer picture in terms of the demographic, geographical, socio-cultural and psychosocial factors.

According to the most recent micronutrient survey in Kenya, the prevalence of anaemia among pregnant women is 55% and 46% among non-pregnant women. This means that pregnant women are most vulnerable to anaemia and subsequent maternal mortality and hence they form the primary target audience for this strategy. Further segmentation was conducted of the primary audience in order to identify the most appropriate and effective way to communicate with the various groups. The key segments selected as priority audiences for interventions are pregnant mothers 25-36 years because they fall within the segment of women of reproductive age with high fertility rates according to KDHS 2008-09. Other women of reproductive age that will be targeted with IFAS interventions include; pregnant mothers 15-19 years and potential mothers[20] 15-25 years. These are the population segments at the centre of the socio ecological model.

The people analysis further reveals that the sexual partner/husband, peers, mother and health providers directly influence the behaviour of the pregnant mothers and hence they are referred to as the secondary audience.

The table below provides the analysis of the population groups that are most affected, andthose who directly and indirectly influence the primary audience. In the socio ecological model, these are people at the Interpersonal, community and enabling environment levels.

Table 1: IFAS People analysis

PEOPLE ANALYSIS			
**People most Affected (Primary audience)**	**Pregnant women Ages 25 – 36 years**<br>Peril-urban, Married<br>Primary school level<br>Small scale farmer<br>Earns about 5000 a month	**Pregnant women Ages 15 - 19 years**<br>Peri-urban<br>Primary school dropout<br>Married, Unemployed / Casual worker	**Potential mothers Ages 15 - 25 years**<br>Urban<br>In college<br>Rural /urban slums
**People directly Influencing (Secondary audience)**	• Partners /husbands<br>• Peers<br>• Health care providers CHWs (TBAs)<br>• Immediate family e.g. mother, mother-in laws	• Mother,<br>• Partners/husbands<br>• Peers/age mates<br>• Mother in-law<br>• Family<br>• Health care providers	• Peers,<br>• Family Members such as Mother, Father, Siblings,<br>• Teachers<br>• Media & Role Models<br>• Sexual partners / husband<br>• Opinion leaders<br>• Health care providers
**Indirectly Influencing (Tertiary audience)**	• Policy makers (MOH)<br>• NGOs/Partners<br>• Chiefs, local leaders at the lower levels,<br>• Religious leaders<br>• Cultural leaders	• Spiritual leaders,<br>• CHWS (TBAs<br>• Policy makers	• Teachers /lecturers in learning institutions<br>• Policy makers<br>• Religious leaders<br>• Opinion leaders

20 The Potential mother is the woman in the reproductive age that is not pregnant but will be planning to get pregnant in the future.

Figure 4: Malnutrition during pregnancy endangers the health and life of the mother and child

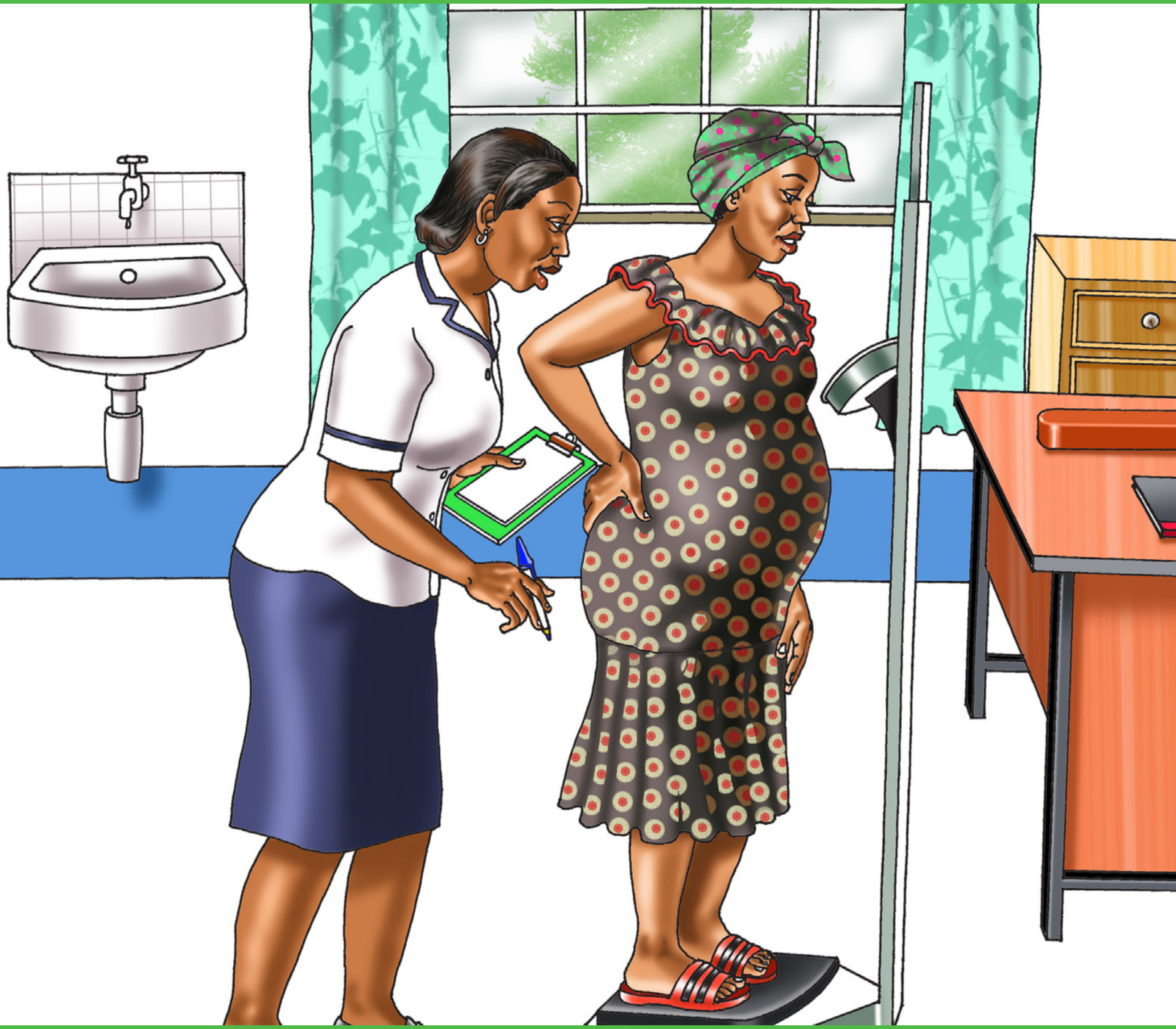

2.5. IFAS CONTEXT ANALYSIS

This segment is an analysis of IFAS contextual issues that affect the behaviour of the individuals. Key issues analysed include: the information that target groups receive on IFAS, the ability for the target groups to act on the information, and how the audiences are affected by policies, systems, services, economics, and social realities. It also explores value systems in terms of social and cultural norms that affect pregnant women and women of reproductive age attitudes, beliefs and behaviour. These issues are illustrated within the socio ecological model as cross-cutting factors.

The purpose of the context analysis is to assist programmers to shift their conceptual thinking to a more holistic level. The factors explored below help in addressing not only knowledge and motivations on IFAS as it relates to the primary target audience but also skills and enabling environment. The analysis is important in understanding the context of the pregnant women.

a. Information needs of pregnant women about IFAs

The information that the pregnant women already know about IFAS is provided under this section. This information is derived from KDHS 2008-09 and FA MOH 2013. It furtheroffers additional information needs that will require to be addressed through communication.

- The pregnant mothers know about ANC and importance of attending ANC but not the importance of initiating ANC early and frequently.
- They are aware of IFA tablets but lack knowledge on all the benefits, dosage, frequency and management of side effects. To address the gaps in knowledge, the following information requirements needs to be given to pregnant mothers:
    - o IFAS dosage, frequency and duration according to the current policy. The policy states that the pregnant should take IFAS daily during the pregnancy
    - o Benefits of IFAs and importance for her pregnancy outcome
    - o Benefits of new combined IFAS information
    - o Side effects of IFA tablets and their management
    - o Causes, symptoms and effects of Anemia
    - o Consequences of Anemia in pregnancy if left untreated
    - o General nutritional needs during pregnancy and consequences of malnutrition during pregnancy
    - o Food sources of iron and folic acid
    - o Other strategies of anaemia prevention like malaria control and deworming
    - o Importance of using the MCH booklet to monitor her health and to carry it during each visit
    - o Importance of joining a mother to mother support group

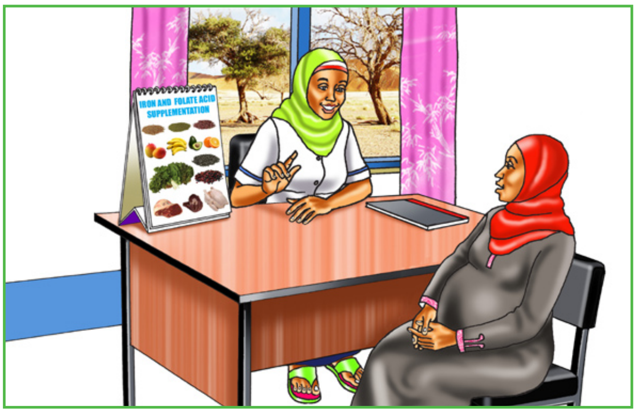

Figure 5: Nurse counselling a mother on nutrition and micronutrients

b. Enabling Environment for IFAs

The environmental issues indirectly affect the behaviour of pregnant women. The key issues addressed here are policies that affect pregnant women, availability and accessibility of services as well as religious or cultural pressures that affect their behaviour. It seeks to address both the existing policies and services and existing gaps because it either becomes a barrier or facilitator of desired IFAS actions. This analysis defines other areas of interventions besides individual behaviour.

- ANC services and IFA supplements are available at no cost in public health facilities.
- Availability of supportive IFAS policies and ANC services package.
- Different cadres of health workers have received pre-service and in-service training and are engaged in IFA supplementation through multiple service delivery points.
- IFA supplementation for pregnant women is routinely delivered in all public health facilities through Maternal and Child Health (MCH) clinics as part of the Antenatal Care [FANC].
- IFA supplementation is often interrupted due to stock-outs and lack of supply.
- Lack of a proper system to monitor distribution of IFA supplements and indicators.
- The current policy only allows professional health workers to prescribe and dispense IFA supplements to pregnant women but not community health workers (CHWs).
- No specific policies targeting the 15-19 year old teenagers; they have limited maternal care services.
- Minimal support in the urban poor unlike in rural areas.

c. Ability to act on IFAS information

The purpose of this is analysis to establish if the pregnant mothers are able to act on what they know about IFAS. It helps to determine additional approaches to use to reach out to the pregnant mothers that will enhance their confidence and skills to achieve the desired behavior.

- The pregnant mothers have inadequate information on IFAS to enable them to take appropriate action. This is specifically on dosage, frequency, duration, side effects and their management.
- They have limited information on when to initiate ANC visits and recommended number of visits for optimal care. This leads to inconsistency in ANC care results and missed opportunities for IFAS.
- They rely on health provider's advice and can act if adequate information on IFAS and ANC is provided.
- They have limited information and resources to implement dietary diversification.
- Availability of new combined IFA tablets which are more palatable as they are enteric coated and make it easy to take since they will need fewer tablets hence they are expected to improve adherence.
- Poor dissemination of the IFA policy will negatively affect IFA uptake.

d. Values, Norms, Attitudes, beliefs

Behaviour of individuals is influenced greatly either positively or negatively by values, social norms, attitudes and beliefs. The BCC approaches will be designed to address or build on the positive issues.

- Mothers value having a safe pregnancy, delivery and healthy baby.
- They value community and family support systems, especially the husband/partner, mother and peers.
- They value the health system support like availability of ANC services and health provider support.
- Majority initiate ANC in the second trimester because of perceived good health and fear of HIV testing among other reasons.

Figure 6: Examples of Fruits, Vegetables, Legumes and Meats for Promoting Healthy Weight Gain”

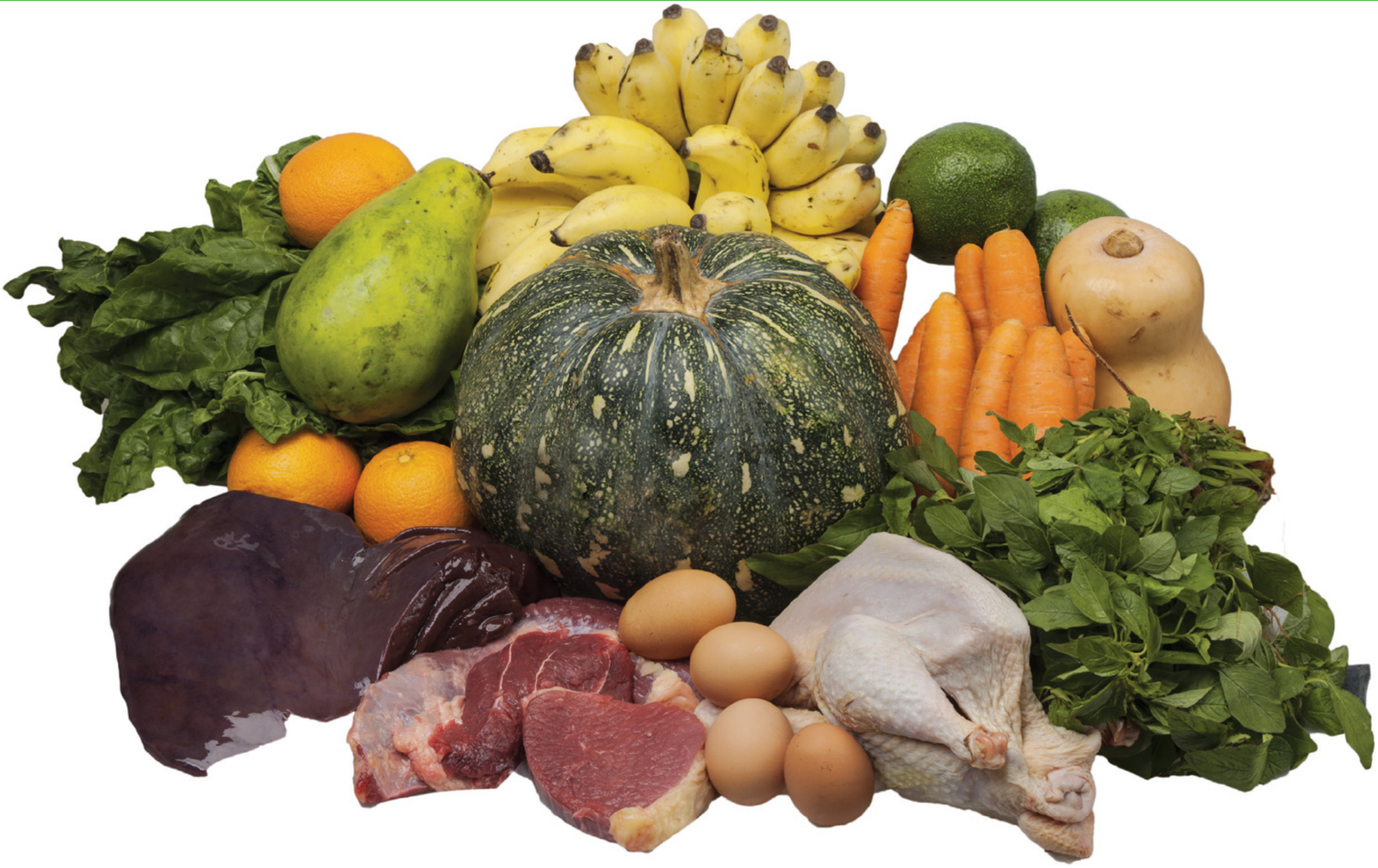

2.6. BEHAVIOUR CHANGE ANALYSIS FOR PRIORITY TARGET AUDIENCES

After understanding the problem, people affected and contextual issues, desired behaviours are identified for each target audience that will aim at contributing to improvement in coverage and utilization of IFAS. An analysis has been provided of the various issues from desk review findings and the recently conducted IFAS formative research on the changes that the target groups need to make regarding their attitudes, behaviour, values, perceptions, skills and any other changes. It highlights obstacles or barriers to the adoption of the desired changes as well as facilitating factors. Communication objectives have been developed to address the obstacles and barriers identified for each target audience.

2.6.1. CHANGE ANALYSIS FOR PREGNANT MOTHERS

An analysis of the desired IFAS changes, the obstacles, communication objectives and the facilitating factors for pregnant mothers has been provided to guide in identifying specific areas of focus in the interventions and messaging.

a. Desired changes

- The pregnant mother needs to know the importance and benefits of IFAs which include; sustaining strength during pregnancy, ensures enough blood stores in the body hence preventing anemia, and prevents low birth weight.
- Knowledge on IFA supplements dosage and duration and where to get them.
- Take complete dose of combined IFAS, which is taken daily during pregnancy.
- Knowledge on how to manage the side effects should they occur and importance of IFAS adherence
- Knowledge on causes, symptoms and effects of anaemia and seeking early care.
- knowledge on the foods rich in iron and folic acid.
- Consume a variety of locally available foods particularly those rich in iron and folic acid.
- Those in malaria endemic areas should take intermittent preventive treatment (IPTp) and sleep under Long Lasting Insecticide Treated Nets (LLIN).
- Deworm regularly to prevent worm infestation.
- Plan and space her next pregnancy.
- Take folic acid before conception and within first month of pregnancy to prevent neural tube defects.
- Attend ANC as soon as she knows she is pregnant and conduct monthly visits during pregnancy.

b. Obstacles and facilitating factors

Some of the barriers and obstacles pregnant mothers face in achieving the desired changes with regard to IFAS include; lack of knowledge on importance of initiating ANC early and frequent ANC attendance which may be attributed to fear of HIV testing and perception of good health, lack of time and money, distance to the clinic among other reasons. Other barriers include; inadequate information on IFA supplements, side effects and their management, as well as dosage, frequency and duration, pregnant mothers lack of knowledge on importance and effects of folic acid deficiency. They also lack knowledge on optimal nutrition practices during pregnancy & feeding practices predisposing IFAS deficiency.

Some of the health facility barriers include: inadequate counseling on IFAS by health service providers especially on duration of intake, side effects and reasons for using IFAS, low knowledge level among health service providers on IFAS and erratic supplies of IFA supplements leading to stock outs.

Some of the facilitating factors for desired behavior are availability of IFA supplements in health facilities at no cost to the mother, availability of combined IFAs formulation, accessibility of ANC (services and commodities), high awareness of ANC, positive attitude on ANC, supportive social system (family, peers), supportive policy and environment, functioning national IFAS distribution system and IFAS is integrated in the community strategy package for CHWs.

c. Communication objectives

The goal of the strategy is to increase the utilization of IFAS among pregnant mothers as per the current policy by 2017 resulting to:

- Increased number of pregnant mothers who know the benefits of IFAS, dosage, timing, duration, side effects and their management.
- Increased number of pregnant mothers taking a complete dose of IFAS as per the guidelines
- Increased number of women who understand the effects of anaemia and are taking preventive measures.
- Increased number of pregnant mothers initiating ANC early in the first trimester regardless of their nutritional/health status.
- Increased number of pregnant mothers who know how to manage side effects when they occur.
- Increase number of pregnant mothers who know good dietary practices (diversity, frequency, amount) during pregnancy.
- Increased risk perception among the potential mothers.
- Increase numbers of potential mothers aware of folic acid deficiency and prevention measures.

Figure 7: IFAS supplements ensure a healthy pregnancy and healthy baby

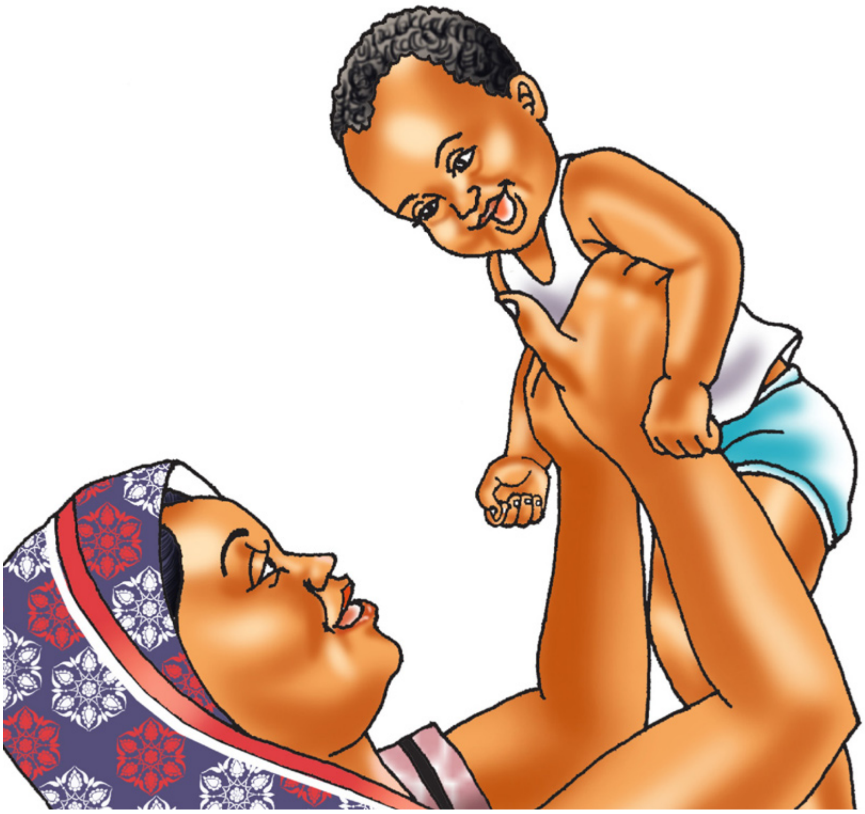

2.6.2. CHANGE ANALYSIS FOR HEALTH CARE PROVIDERS ON IFAS

a). Desired behaviours
The same analysis of desired changes, barriers to desired change, facilitating factors and communication objectives has been done for frontline health care service providers who have contact with pregnant mothers.

- o Counsel pregnant mothers on importance of early and frequent ANC visits.
- o Know the rationale for prescribing IFAS to all mothers and prescribe IFAS to all women regardless of the HB/anaemia status (WHO guideline for countries where anaemia is>40%).
- o Counsel mothers on importance of IFAS, howand when to take the IFAS.
- o Prescribe complete dosage to be taken daily during pregnancy as per the policy.
- o Know about the new combined IFAS formulation and current IFAS policy; 60mg iron/400Ug folic acid to be given throughout pregnancy.
- o Counsel pregnant mothers how to manage any side effects when taking IFAS.
- o Provide IFAS within a package of integrated health services.
- o Administer and monitor IFAS correctly.
- o Counsel on diet diversification.
- o Provide deworming tablets regularly.
- o Provide IPTp and nets to pregnant women in malaria endemic areas.

b). Obstacles and facilitating factors

Some of the obstacles facing health providers in fully providing IFAS services are; inadequate knowledge on IFAS policy guidelines, erratic supply of IFAS commodities, inadequate staffing leading to high workload and low quality service e.g. individualized counseling, inadequate IFAS BCC materials, lack of IFAS summary tool, current policy of screening for anaemia before prescribing IFAS,IFAS not part of continuous medical education (CMEs), and limited focus of IFAs on the job training (OJTs).

Some of the facilitating factors are; existing IFAS policy, GOK currently procuring combined IFAS, established supply chain system for IFAS, functional community units in existence and functional health care system.

c). Communication objectives
The goal is to increase the utilization of IFAS among pregnant mothers as per the current policy by 2017 resulting to:

- o Increased number of health care providers who prescribe IFAS to all women regardless of HB status per the policy.
- o Increased number of health care providers who counsel on; importance of IFAs, duration and dosage.
- o Increased number of health care providers who counsel pregnant mothers on management of side effects.
- o Increased number of health care providers who provide information on importance of anaemia prevention and effects of anaemia during pregnancy.
- o Increased number of health care providers who provide information on good dietary practices (diversity, frequency, amount) during pregnancy.
- o Improved capacity of health care providers to administer and monitor IFA commodities use.

Be a champion;

give pregnant women the chance to have a healthy pregnancy.

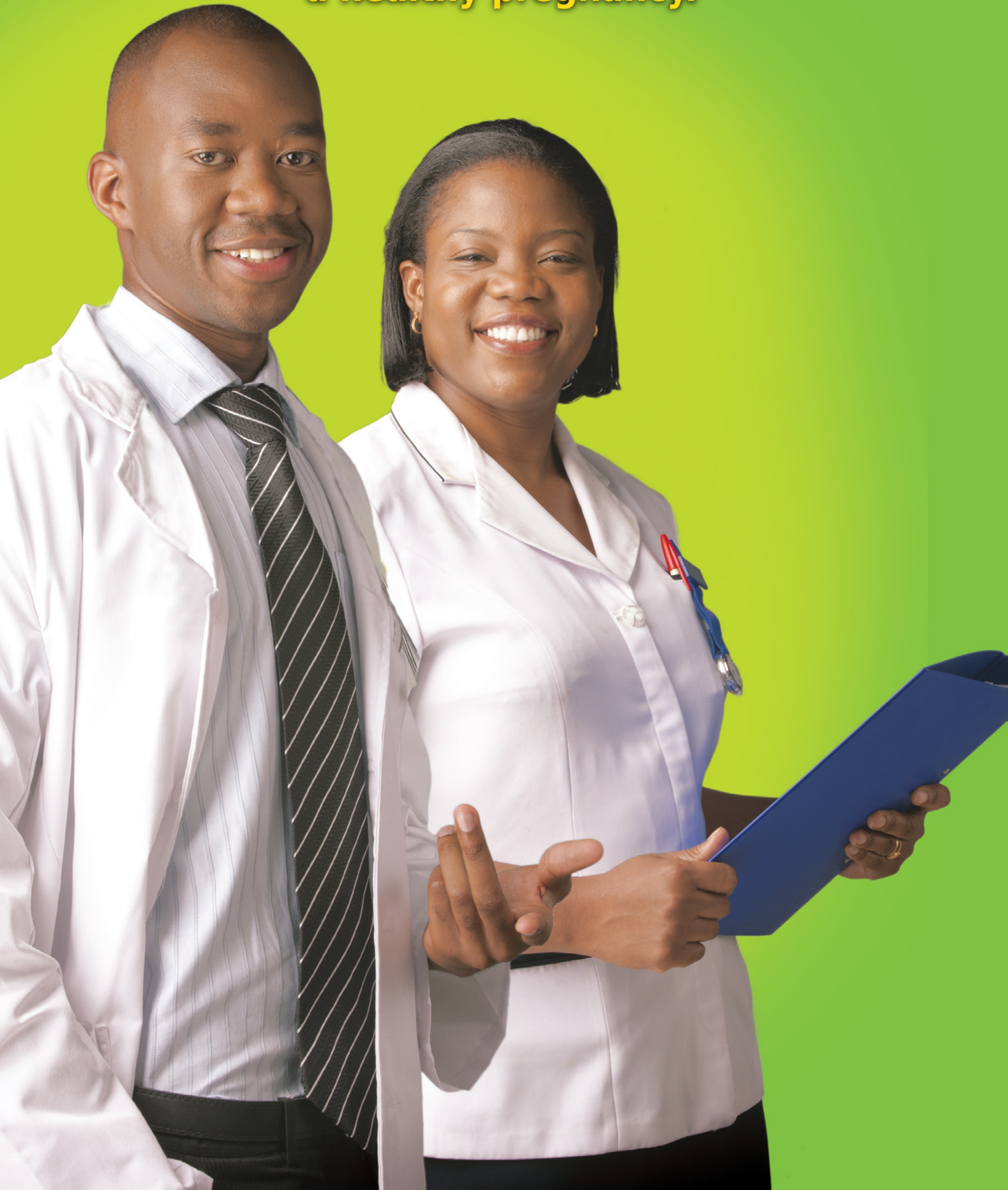

2.6.3. CHANGE ANALYSIS FOR COMMUNITY HEALTH WORKERS

a) Desired behaviour

- The CHWs are expected to encourage pregnant mothers to seek ANC services early and conduct monthly visits so that they can access IFAS in addition to other services
- Encourage pregnant mothers to access IFAS from health facility
- Talk to pregnant mothers about importance of IFAS for healthy pregnancy outcome
- Conduct dialogue sessions with pregnant mothers on the following:
    - Nutrition demands during pregnancy
    - Causes, symptoms and effects of anaemia during pregnancy
    - Benefits of combined IFAS
    - Side effects of IFAS and their management
    - Prevention of malaria and chronic worm infestation
    - Talk to mothers about good dietary practices (diversity, frequency, amount) during pregnancy
    - Monitor pregnant mothers taking IFAs to improve adherence.

b). Obstacles and facilitating factors

Some of the barriers that CHWs face in achieving desired IFAS behaviors are; limited knowledge on the IFAS policy, lack of messages and materials on IFAS, IFAS policy not disseminated to all health facilities, low motivation and increased workload/limited time

The facilitating factors for desired behavior on the part of the CHWs include; existence of community strategy structures, functional community units, availability of ANC services, referral system from community already established, availability of the services and commodities at the facility, and existence of guidelines and policies.

c). Communication objectives

- Increased number of CHWs who refer pregnant mothers for early ANC
- Increased number of CHWs who provide messages on IFAS to pregnant mothers
- Increased number of CHWs who monitor pregnant mothers taking IFAS to improve adherence.

2.6.4. CHANGE ANALYSIS FOR INFLUENCERS

The people who directly influence the pregnant mothers known here as influencers have been identified as husbands/partners, mothers and mothers in law. They have a critical role in supporting the pregnant mothers in working towards the IFAS desired behaviors.

a). Desired Changes

- They need to know about the importance of IFAS to the mother and unborn baby,
- Know about importance of early and frequent ANC visits for the mother and baby,
- Accompanying pregnant mothers to the ANC clinics and ask about IFAs.
- They also need to remind pregnant mother to take IFAS daily during pregnancy and provide support for enriched diet for the pregnant mother.

b). Obstacles and facilitating factors

The influencers have barriers to desired behavior such as; inadequate information on ANC services, inadequate information on IFAS, lack of known paternal role in MCH services, negative societal expectation on male involvement in ANC/MCH issues, cost associated with attending ANC servicesand inadequate knowledge on appropriate dietary practices during pregnancy. Some of the facilitating factors for influencers are availability of ANC services and IFAS at public health facilities and family support.

c). Communication objectives

- Increased awareness on their role in improving adherence of IFAS among the pregnant mothers.
- Increased awareness on their involvement in ANC service delivery.
- Increased knowledge on importance of pregnant mothers attending ANC early and taking IFAS daily during pregnancy.

2.6.5. CHANGE ANALYSIS FOR POLICY MAKERS

a). Desired behaviours

The policy makers are people who indirectly influence the affected individuals. They are expected to have adequate knowledge on IFAS. For sustainability purposes, they need to invest resources for full dissemination of IFAS policy, invest resources to sustain availability of IFAS supplies and allocate resources to improve structures to support IFAS implementation and M & E systems. Other desired actions include; prioritization of IFAS both at national and regional agenda in terms of human resources, and finances.

The obstacles that policy makers face in attaining desired actions includes lack of adequate information on importance of IFAS and Inadequate resources as well as competing priorities. The facilitating factors for the achievement of desired actions includes availability of research and evidence, commitment and leadership at MOH, availability of resources to procure IFAS and there is knowledge on global emerging issues and priorities on IFAS.

b). Communication objectives

The communication objectives related to policy makers on IFAS are:

- Improved resource allocation for IFAS at national and county levels to sustain IFAs supplies.
- Improved resource allocation for full dissemination of IFAS policy to all health facilities.

2.7 IFAS KEY MESSAGE THEMES

IFAS Behavior Change Message themes

This strategy has systematically addressed various components which build onto each other. The messages are based on analysis, strategic design and understanding of the problem, people, context and barriers discussed in preceding sections of this document. The message themes are derived from the communication objectives developed for each target audience. The messages provide the most compelling solutions which at least address the obstacles the audiences face. The messages need to be communicated at multiple levels to multiple audiences where focus is not only on individual change but on creating or reinforcing social conditions and social relationships that facilitate and encourage healthy behaviour.

The table below provides message themes for the five target audiences prioritised in this strategy. These are pregnant mothers, healthcare providers, policy makers, influencers and community health workers. The shaded sections indicate the message themes that are relevant to that target audience, while the un-shaded sections indicate the messages do not apply to them.

The purpose of developing message themes is to help standardize the IFAS information disseminated in all the counties to ensure consistency and accuracy of messaging. The different regions can contextualise the messages to suit the different cultural settings but the accuracy of the messages should be maintained.

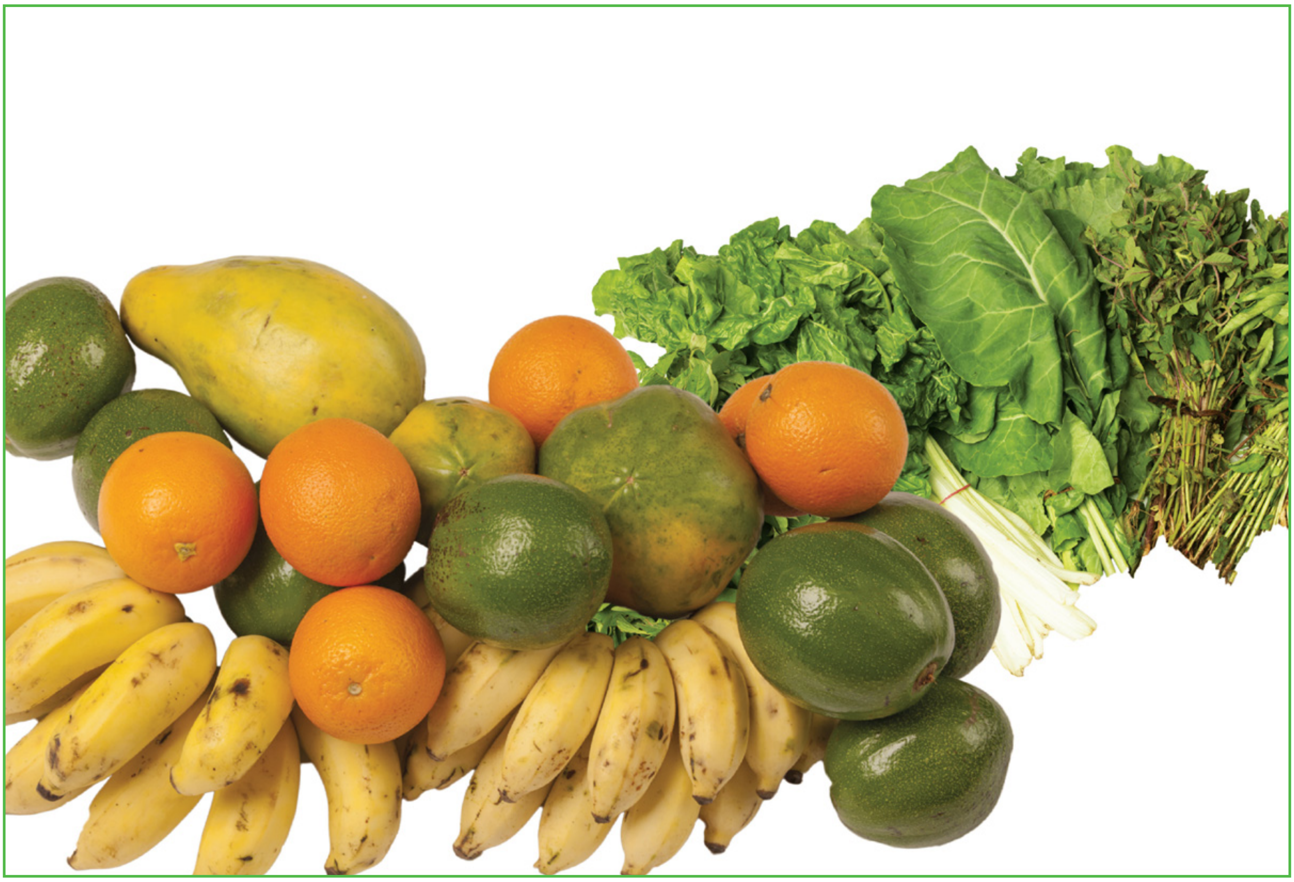

Table 2: IFAS messages themes

Message themes	Pregnant mothers	Health providers	Policy makers	Influencers	Community health workers
**Knowledge and actions on ANC:** Early ANC attendance and continue with monthly visits					
**Knowledge and Actions on IFAS:** Importance and benefits of combined IFAS, dosage, frequency and duration of IFAs and where to get the supplements, possible side effects and their management					
**Knowledge and Actions on anaemia:** causes ,symptoms and effects of anemia					
**Knowledge and actions on nutrition during pregnancy:** nutrition demands in pregnancy, consequences of malnutrition during pregnancy for both the mother, fetal and infant health. Food diversification and foods sources rich in iron and folic acid					
**Knowledge and Actions on other strategies for anaemia prevention:** all pregnant mothers in malaria endemic areas should sleep under an LLIN, take at least two doses of intermittent preventive treatment					
**Knowledge and Actions on IFAS practice for healthcare providers:**<br>• Counselling pregnant mothers on: Importance and benefits, dosage and duration of IFAs and where to get the supplements, managing side effects.<br>• Counsel on nutrition demands during pregnancy, consequences of malnutrition for mother, fetal and infant, IFAS food sources					

Table 2: IFAS messages themes

Message themes	Pregnant mothers	Health providers	Policy makers	Influencers	Community health workers
• Rationale for prescribing IFAs to all pregnant mothers rregardless of the HB/anaemia status (WHO guideline for countries where anaemia is>40%<br>• Know about the new combined IFA formulation and current IFA policy; 60mg iron/400Ug folic acid to be given throughout pregnancy<br>• Provide IFAs within a package of integrated health services<br>• Administer and monitor IFA correctly					
**Knowledge and actions for community health workers:**<br>• Educate mothers on nutrition demands in pregnancy, causes, symptoms and effects of anaemia during pregnancy, benefits of IFAS, side effects of IFAS and their management, Malaria and chronic worm infestation prevention.<br>• Accompanying pregnant mothers to the ANC clinics and ask about IFAs<br>• Reminding pregnant mother to take IFAs daily during pregnancy					
**Knowledge and Actions for policy makers**<br>• Invest resources for full dissemination of IFAs policy<br>• Invest resources to sustain availability IFA supplies.<br>• Allocate resources to improve structures to support IFA implementation and M & E<br>• Prioritization of IFAs both at national and regional agenda in terms of human resources, and finances					

2.8. COMMUNICATION CHANNELS AND MATERIAL IDEAS

This strategy will utilise three basic channels to create the desired changes at multiple levels in order to achieve the outlined IFAS outcomes. A mix of mutually reinforcing channels has been creatively put together to increase effectiveness of the interventions. These channels are Interpersonal communication, community based channels and mass media.

The interpersonal communication channels will enable one on one communication as well as small group interactions. They are more interactive and will assist in unpacking ANC and IFAS underlying information; it can build behavioural skills and increase self-efficacy and intentions to act. The healthcare providers and community health workers will play a primary role in facilitating interpersonal communication with the pregnant mothers.

The community based channels planned for IFAS interventions have been designed to reach communities and will be dealing with community-wide mobilization for support on IFAS issues. The intention of these channels is to stimulate dialogue on IFAS, motivate collective solutions, and provide social support and feedback to broader community. The CHWs, partners and stakeholders will be instrumental in facilitating these interventions.

The mass media both print, social media and electronic media will be used to reach a large audience on IFAS messages in a short period of time. New media and social media can be used to reach those in urban areas.Women in rural and low income urban areas generally have limited access to print media due to cost and availability; they do not purchase daily newspapers and may only get exposure on the few occasions when their male partners do or bring a copy home from work. Magazines that appeal to this target (e.g. Parents) are limited, expensive and mostly available in urban areas. BCC campaigns have demonstrated that radio and TV deliver the highest reach for populations in rural and low income urban areas

Summary of key channels and materials for each target group

a) **Channels for pregnant mothers:** Interpersonal communication and community based media through focussed community dialogue days on IFAS, door to door IFAS education, IFAS health talks, facility based groups like mother support groups, and community women groups. Mass media campaign will be conducted through national and regional Radio and TV stations, out of home media like wall branding in markets, shopping centres, water collection points and buses, motor bikes branding, advertising in screens at the health facilities, women magazines and mobile phone reminders with IFAS messages. Social media (face book, twitter) can be used to reach out to pregnant mothers in urban areas.

   - BCC materials to use for pregnant mothers includes posters, leaflets, mothers' journal, flip charts, promotional materials like bags, lessos, wrist bands, umbrellas, IFAS video documentary.

b) **Channels for healthcare service providers and health managers:** Capacity strengthening of health managers and frontline service providers at the MCH. This includes training, supportive supervision, continuous medical education and integrated outreach.

   - BCC materials to use includes IFAS training guide, IFAS policy guidelines, job aids, supervision checklist, monitoring tools, badges and pens.

c) **Channels for Influencers (husbands, mothers and mother in laws[20])**:Interpersonal communication through focussed dialogue on IFAS with men, interactive sessions with men on radio and community groups, road show vans or tracks at shopping centres, mobile cinemas, video documentaries in video dens.

- BCC materials for this group includes fact sheets, posters, video and CDs, promotional materials like T-shirts and caps.

d) **Channels for community health workers:** Interpersonal communication through IFAS training, sensitization and follow up. Testimonials for success stories with CHWs.

- BCC materials for CHWs are CHW dialogue cards, promotional materials like T-shirts, bags, lessos, fliers/brochures, badges.

e) **Channels for policy makers:** Interpersonal communication through policy dialogue on IFAS at different forums like conferences, symposiums, meetings. IFAS Social Mobilization through stakeholder forums at National, County and Sub-County level. Mass media will also be used through both print and electronic media.

- BCC materials to use for policy makers includes fact sheets, IFAS policy guidelines, research findings, talking points, policy documents and statements.

20 Mothers and mother in-laws with be reached through the same approaches designed for pregnant mothers

2.9. BEST PRACTICES ON IFAS UTILIZATION AND COVERAGE

This section highlights some of the case studies from three different countries that demonstrate the success stories with regards to anaemia control, and improved coverage and utilization of IFAS. The interventions in this strategy have been informed by some of these case studies successes highlighted below.

Figure 9: Case study: Nepal

CASE STUDY: PANDEY-NEPAL

Nepal adapted a community-based integrated intervention to improve coverage of and compliance with iron supplementation in Nepali women. The Introduction of a program with new delivery mechanisms was launched involving training sessions for all the stakeholders, health workers and Community Health Volunteers (CHVs). In the implementation strategy, the CHV were supposed to identify pregnant women and distributed the tablets to them. Women could also get the tables from the health facility. A mechanism to ensure that women do not obtain the tablets from two sources at once was also put put into place. The use of the CHV helped in improving access.

As per the strategy, the CHV repacked tablets in small 30-tablet plastic containers and gave them to pregnant women asking them to bring the containers for a recount and refill each month. Continuous counselling of the mothers of the importance of compliance was done by the CHVs and the health workers. This supported in improving compliance. Standardized registers were maintained for all enrolled clients thereby facilitating effective monitoring and follow up. The outcome of this strategy was an increased coverage in IFA supplementation.

Figure 10: Case Study: Thailand

CASE STUDY: THAILAND

Over the past several decades, Thailand has dramatically reduced the prevalence of under nutrition in children under 5 years old. Thailand made an early commitment to anaemia control, citing it as a major nutrition problem as early as 1982 and establishing anaemia reduction as a national goal. Although Thailand has never had a permanent national committee for anaemia control, it has used ad hoc committees of scientists and program managers to examine and discuss issues related to anaemia prevention and control. To reduce anaemia among pregnant women, Thailand took the following actions:

- Increased the early use of antenatal care (ANC) services. With the help of 500,000 village health volunteers, 98 per cent of women receive ANC and 84 per cent make at least four ANC visits. The volunteers identify pregnant women and encourage them to obtain ANC services immediately.
- Provided iron-folic acid (IFA) supplements to all pregnant women, regardless of their haemoglobin levels.
- Used qualitative research findings to improve communication and help health workers counsel women taking IFA supplements
- Decentralized the supply and logistics of IFA supplements by permitting provincial offices to estimate their own needs and provided easily accessible back-up supplies
- Monitored anaemia prevalence in young children and women at public health service facilities

Figure 11: Case study: Indonesia

CASE STUDY: INDONESIA

Anaemia prevalence is high in a number of groups throughout Indonesia. After initiating efforts to reduce anaemia in pregnant women in 1985, Indonesia has since expanded its anaemia prevention and control strategies and is addressing anaemia prevalence in other vulnerable groups through a number of measures.

National survey data show that anaemia prevalence in pregnant women decreased from 73.7 percent in 1985 to 50.9 per cent in 1995, a 31 per cent decline. This suggests that progress had indeed occurred by the mid-1990s. The change in anaemia prevalence in pregnant women accompanied increased attention to anaemia prevention and control in antenatal care (ANC) services. Several good practices were identified as responsible for the improvements in this program:

- High commitment from the Indonesian government to control anaemia
- Monitoring systems for anaemia and iron-folic acid (IFA) supplement use Improved packaging to protect IFA supplements from humidity and make them more attractive to consumers
- A change to a red, film-coated supplement that did not have the fishy taste of the previous supplements
- Messages about when and how to take IFA supplements to mitigate side effects
- Increased supply and availability of IFA supplements at each level of the health system, including distribution by community health workers (village midwives and traditional birth attendants) and private sector sales by drug vendors and small shops
- Availability of program guidelines and protocols on how many IFA supplements to give

These improvements have increased the number of pregnant women receiving and taking IFA supplements.

CHAPTER 3: IFAS IMPLEMENTATION PLAN

This is a multi-year implementation plan designed to address all the interventions in this strategy in the next five years. The strategy uses multiple and mutually reinforcing interventions to create impact. The emphasis is on strengthening the existing systems and structures to ensure continuous improvement in coverage and utilization of IFAS.

- The interpersonal communication and community based interventions will be implemented in phases in different regions starting with regions with a high volume of pregnancies to improve coverage. The regions with high anaemia prevalence will also be prioritised, especially those with iron deficiency related anaemia. With dedicated resources, scale up will be achieved in a period of five years.
- However the mass media interventions will be national, implemented both at the national and regional level with focussed IFAS campaigns to create awareness and build demand for appropriate actions. In the span of five years, it is advisable to conduct two high impact campaigns.
- Capacity strengthening interventions for health managers, both County Health Management Teams and District Health Management Teams, will be national and implemented in the initial phases of the program so that they can cascade the training to the frontline service providers and CHEWs and CHWs.

Summary of the broad strategies in the implementation plan
The activities in the implementation plan defines how the five broad strategies will be implemented and the proposed timelines.

- **Strategy One:** Advocate for Increased Resources for IFAS at the National and County level
- **Strategy Two:** Empower Pregnant Mothers to Adopt Appropriate Health Practices and Health Seeking Behaviour on ANC and IFAs Through Behaviour Change Communication Interventions.
- **Strategy Three:** Create an Environment that Supports Adoption of Positive ANC and IFAS Practices through Social Mobilization.
- **Strategy Four:** Capacity Strengthening for Health Providers on IFA Supplementation To Increase Utilization Rates.
- **Strategy Five:** Empower Community Health Workers on IFA Supplementation to Increase Coverage and Utilization Rates.

Figure 12: IFAS IMPLEMENTATION PLAN

ACTIVITIES	TIME SCHEDULE																			
	2013 Q1 to Q4				2014 Q1 to Q4				2015 Q1 to Q4				2016 Q1 to Q4				2017 Q1 to Q4			
	I	II	III	IV	I	II	III	IV	I	II	III	IV	I	II	III	IV	I	II	III	IV
**Strategy One: Advocate for Increased Resources For IFAS at the National and County level**																				
Advocacy to policy and decision makers at the National and County levels to increase resources and build institutional commitment for IFAS through conferences, symposiums, breakfast meetings																				
Media engagement at National and county level to increase quantity and quality of reporting on IFA supplementation: Sensitize journalists on IFAS , Monitor IFAs reporting, Conduct TV and Radio talks shows with subject matter experts																				
**Strategy Two: Empower Pregnant Mothers To Adopt Appropriate Health Practices And Health Seeking Behaviour on ANC and IFAs Through Behaviour Change Communication Interventions**																				
Mass media campaigns: Conduct a robust IFAs mass media campaign through both print, electronic and social media to increase demand for the services and facilitate social and behaviour change.<br>o Advertising in TV screens placed at health facilities<br>o Radio and TV:- National and local FM stations<br>o Radio/TV spots, Live discussions, testimonial sound bites<br>o Mobile phone communication<br>o Video documentary<br>o Magazines- in salons<br>o Talking walls and buses: Public service vehicles, bus stops/ terminus, markets, shopping centers, water collection points																				
Interpersonal communication channels: Conduct one-on one interactive communication interventions to increase dialogue on IFA supplementation and ANC.<br>• Mainstream IFAS Health talks at health facility<br>• Work with existing facility based group's e.g. Mother to Mother support groups or mentor group																				

Figure 12: IFAS IMPLEMENTATION PLAN

ACTIVITIES	TIME SCHEDULE																			
	2013 Q1 to Q4				2014 Q1 to Q4				2015 Q1 to Q4				2016 Q1 to Q4				2017 Q1 to Q4			
	I	II	III	IV	I	II	III	IV	I	II	III	IV	I	II	III	IV	I	II	III	IV
**Community basedchannels for pregnant mothers:** community dialogue, community media, to motivate collective solutions, provide social support and feedback to broader community<br>• Conduct Focused Community dialogue days on IFAs with; Community Women groups and church groups<br>• Skits and poems with IFAS messages on market days<br>• Use of role models as positive deviants<br>• Use of IFAS brand ambassadors<br>• College & community storms/ activations for potential mothers<br>• Road show vans or tracks stopping at market centres & high traffic venues<br>• Promotions with incentives<br>• Giveaways (Scarfs/caps/t-shirts, re-usable shopping bags, wrist bands, lessos, umbrellas, cups for feeding children, stickers-bumper and baby on board)																				
**Community based channels for influencers (husbands)**<br>• Conduct focused dialogue with men on IFAs in male support groups, men groups in churches<br>• Conduct Interactive sessions with men i.e Radio or TV breakfast shows<br>• Video documentaries in video dens																				
**Strategy Three: Create an Environment That Supports Adoption Of Positive ANC And IFAS Practices Through Social Mobilization**																				

Figure 12: IFAS IMPLEMENTATION PLAN

ACTIVITIES	TIME SCHEDULE																			
	2013 Q1 to Q4				2014 Q1 to Q4				2015 Q1 to Q4				2016 Q1 to Q4				2017 Q1 to Q4			
	I	II	III	IV	I	II	III	IV	I	II	III	IV	I	II	III	IV	I	II	III	IV
**Mobilize and coordinate ANC and IFA partners and stakeholders at National and County levels to create wider participation, coalition and ownership of IFAs interventions**<br>• Conduct IFAs stakeholders forums at all levels (National, County, Sub-County level)																				
Conduct community mobilization activities through existing structures at the County and Sub-county level to create an environment that supports appropriate social and behaviour change actions that lead to sustainable change.<br>• Community IFAs sensitization meetings through the CHCs, CHEWs, CHWs																				
**Strategy Four: Capacity Strengthening For Health Providers On IFA Supplementation To Increase Utilization Rates**																				
• Summarize the IFAs policy, print and disseminate at all levels<br>• Build capacity of health providers on IFAs, conduct OJTs, and CMEs<br>• Develop IFAs supportive supervision checklist and conduct regular support supervision<br>• Develop and advocate for integration ofIFAs summary monitoring tools																				
**Strategy Five: Empower Community Health Workers On IFA Supplementation To Increase Coverage And Utilization Rates**																				
• Conduct IFAs orientation meetings for CHWs<br>• Facilitate CHW IFAs activities at community level, including Household visits<br>• Facilitate focused Community dialogue on IFAs with; Community Women groups and church groups<br>• Testimonials of success stories with CHWs																				

Figure 12: IFAS IMPLEMENTATION PLAN

**ACTIVITIES**	**TIME SCHEDULE**																			
	2013 Q1 to Q4				2014 Q1 to Q4				2015 Q1 to Q4				2016 Q1 to Q4				2017 Q1 to Q4			
	I	II	III	IV	I	II	III	IV	I	II	III	IV	I	II	III	IV	I	II	III	IV
**Materials and products to be developed for different pregnant mothers, influencers, health providers, community health workers, policy makers**																				
**Develop, produce and print the following materials to support the activities above;**<br>**Pregnant mothers**<br>• Posters, leaflets, mothers journal, flip charts, promotional materials like bags, lessos, wrist bands, umbrellas, IFAS video documentary																				
**Health providers**<br>• IFAS training guide, IFAS policy guidelines, job aids, supervision checklist, monitoring tools, badges and pens																				
**Influencers**<br>• Leaflets , Posters , Video/ Audio tapes/CDs																				
**Policy makers**<br>**Fact sheets, IFAS policy guidelines, research findings, talking points, policy documents and statements**																				
Community Health Workers<br>CHW dialogue cards, promotional materials like T-shirts, bags, lessos, fliers/brochures, badges																				
Conduct monitoring and evaluation of the interventions																				

CHAPTER 4: SUMMARY BUDGET

This section provides a summary estimated budget of activities for a period of one year. These are activities to be conducted nationally in all counties. A total of KES 77,000,000 is needed to conduct all the activities including mass media campaign for one year. A similar amount can be allocated for each subsequent year.

Figure 13: IFAS BCC Annual Budget

**Strategy**	
Strategy One: Enhance Increased Resources, Political And Social Commitment For ANC And IFA Through Advocacy	
• Policy dialogue: conferences, symposiums, breakfast meetings<br>• Media engagement: journalist sensitization meetings, TV/Radio talk shows.	3,000,000
Strategy Two: Empower Pregnant Mothers To Adopt Appropriate Health Practices And Health Seeking Behaviour on ANC and IFAs Through Behaviour Change Communication Interventions	
Mass media campaign:<br>• Advertising TV screens (healthcare channels) placed at health facilities,<br>• Radio and TV:– National and local FM stations, Radio/TV spots, Live discussions, testimonial sound bites<br>• Magazines- in salons,<br>• Wall branding: Talking walls and buses, motor cycle branding	30,000,000
Interpersonal communication:<br>• Health talks at health facility and<br>• Use of existing facility based group's e.g. Mother to Mother support groups or mentor groups	7,000,000
Community based channels:<br>• Focused Community dialogue days on IFAs with; Community Women groups and church groups<br>• Skits and poems with IFA messages on market days<br>• Use of role models as positive deviants and Use of IFA brand ambassadors<br>• College & community storms/activations<br>• Road show vans or tracks stopping at market centers& high traffic venues<br>• Promotions with incentives for pregnant mothers<br>• Focused dialogue with men on IFAs in male support groups, men groups in churches	
Strategy Three: Enhance Environments That Support Adoption Of Positive ANC And IFA Practices Through Social Mobilization	
• Stakeholders forums at all levels (National, County, Ward)<br>• Community mobilization activities through existing structures at the County and Sub-county level<br>• Community dissemination meetings through the CHCs, CHEWs, CHW	5,000,000

Strategy Four: Capacity Strengthening For Health Providers On IFA Supplementation To Increase Utilization Rates –CHMTS/DHMTS, Frontline service providers	
• Training on IFAs, including OJT<br>• Include IFAs in CMEs training curriculum<br>• Supportive supervision<br>• Support counseling supervision	10,000,000
Strategy Five: Empower Community Health Workers On IFA Supplementation To Increase Coverage And Utilization Rates	
• IFAs orientation<br>• Household visits<br>• Focused Community dialogue on IFAs with; Community Women groups and church groups<br>• Testimonials of success stories with CHWs	7,000,000
IEC materials: Materials and products to be developed for different pregnant mothers, influencers, health providers, community health workers, policy makers	10,000,000
Pre-campaign and post campaign survey	4,000,000
Total KSh	76,000,000

Bibliography

Central Bureau of Statistics. 2010. Economic Survey. Central Bureau of Statistics, Ministry of Planning and National Development. Nairobi.
FAO, 2005. Nutrition Country profile, Republic of Kenya, Nairobi.

Harriet Birungi & Onyango-Ouma, 2006. Acceptability and Sustainability of the WHO Focused Antenatal Care Package in Kenya, Population Council, Nairobi.

Kenya National Bureau of Statistics (KNBS) and ICF Macro, 2010.
Kenya Demographic and Health Survey 2008-09.Calverton, Maryland: KNBS and ICF Macro.

MNP, 2011. Iron Deficiency Anaemia –Kenya, Nairobi.

Republic of Kenya (2012): National Nutrition Action Plan, 2012 -2017.

Republic of Kenya (2007): Draft Food Security and Nutrition Policy.

Republic of Kenya (2008): Draft Food Security and Nutrition Strategy 2008.

Ministry of Health, UNICEF, WHO, MI, and New ERA, 1998. Nepal Micronutrient Status Survey,Nepal.

MI, 2002. Community-based Integrated Interventions improve Coverage of and Compliance with Iron Supplementation in Nepali Women, Nepal.

Central Bureau of Statistics (CBS) [Kenya], Ministry of Health (MOH) [Kenya], and ORC Macro. 2004. Kenya Demographic and Health Survey 2003.Calverton, Maryland: CBS, MOH, and ORC Macro.

Population Council, 2008. Adapting Focused Antenatal Care: lessons from 3 countries, Washington DC.

UNICEF, 2005.
Assessment of Health Based Strategies for National Iron/Folate Supplementation Programme report, Kenya

Stoltzfus Rebecca J. and Dreyfuss Michele L. ,2008. Guidelines for the Use of Iron Supplements to Prevent and Treat Iron Deficiency Anaemia.ILSI PRESS, United States of America.

Government of Kenya (2010).The Constitution of Kenya. Nairobi: The Government of the Republic of Kenya

Republic of Kenya (2008).
Kenya Vision 2030.A globally competitive and prosperous Kenya. Nairobi: Ministry of Planning and National Development and the National Economic and Social Council (NESC).

MOPHS. (2012). Kenya Health Strategic Plan 2012-2017 (Draft). Nairobi: Ministry of Public Health and Sanitation.

MOMS, & MOPHS. (2012).Kenya Health Policy 2012-2030. Nairobi: Ministry of Medical Services and Ministry of Public Health and Sanitation.

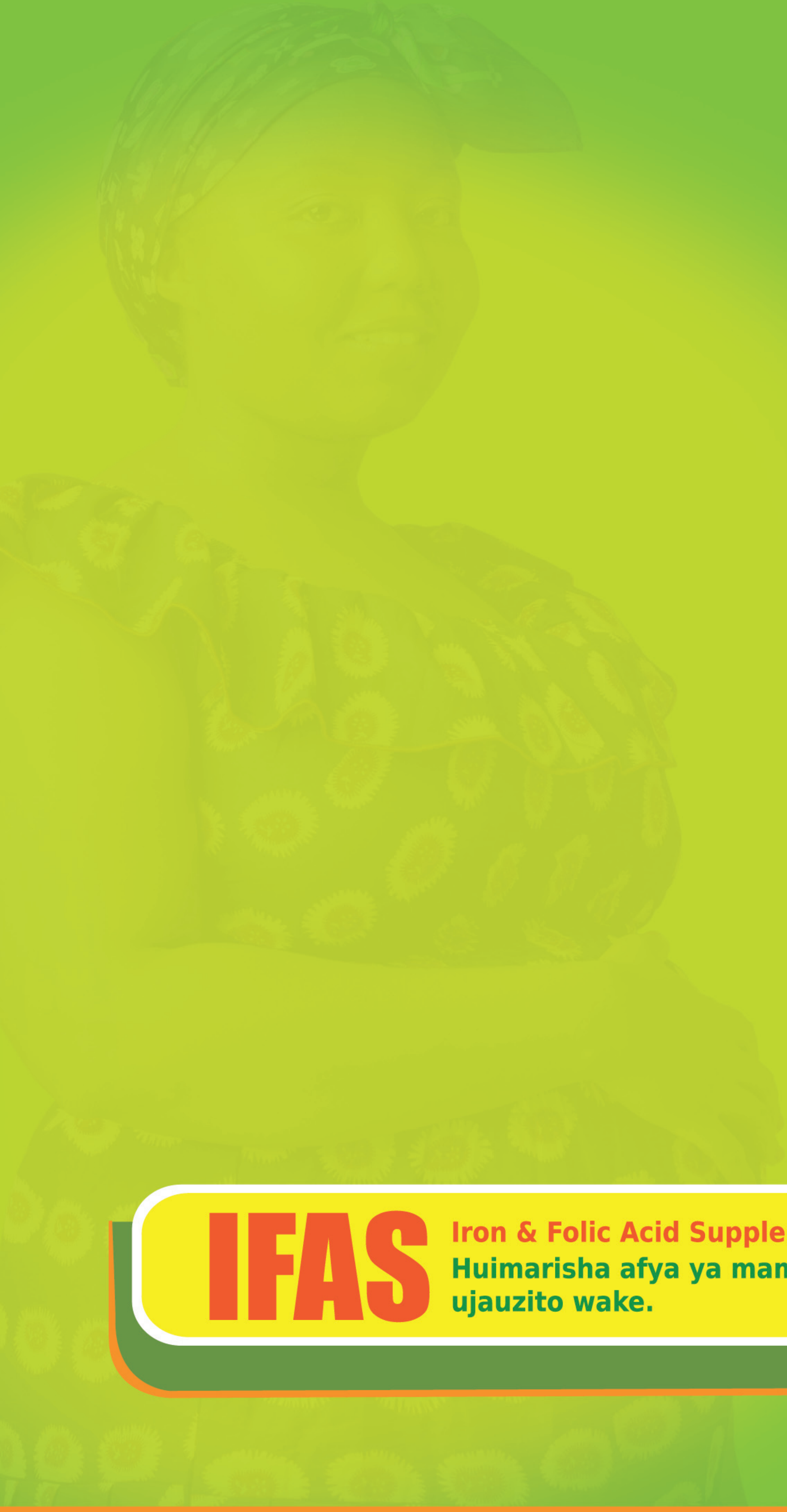

IFAS

Iron & Folic Acid Supplements

Huimarisha afya ya mama na ujauzito wake.
