# Supplementary material for: Time for change is now: Experiences of participants in a community-based approach for iron and folic acid supplementation in a rural county in Kenya, a qualitative study
Source: PLoS One. 2020 Jan 16;15(1):e0227332. doi: 10.1371/journal.pone.0227332 (PMC6964883; doi:10.1371/journal.pone.0227332)
Supplement: S5 File — This is the link where more of the supplementary materials used for IFAS training can be found. (DOCX) [file pone.0227332.s005.docx]

More Information, Education and Communication (IEC) materials on IFAS that were used during the study and IFAS trainings can be accessed on this link:

**Link.**[**https://www.k4health.org/search/site/sites%20default%20files%202013%20kenya%20signed%20ifa%20policy**](https://www.k4health.org/search/site/sites%20default%20files%202013%20kenya%20signed%20ifa%20policy)**.**
